# Genome-Wide Identification and Expression Profiling of Sugar Transport Protein Response to Fusarium Head Blight in Wheat (*Triticum aestivum* L.)

**DOI:** 10.3390/plants14192976

**Published:** 2025-09-25

**Authors:** Yongjiang Liu, Jianfeng Sha, Suhong Zhang, Yawen Sun, Zhiruo Hu, Haigang Ma, Hongxiang Ma

**Affiliations:** 1 Jiangsu Key Laboratory of Crop Genomics and Molecular Breeding/Zhongshan Biological Breeding Laboratory/Key Laboratory of Plant Functional Genomics of the Ministry of Education, Agricultural College, Yangzhou University, Yangzhou 225000, China; dx120210122@stu.yzu.edu.cn (Y.L.); mx120230795@stu.yzu.edu.cn (J.S.); mz120221359@stu.yzu.edu.cn (S.Z.); mz120231380@stu.yzu.edu.cn (Y.S.); mz120241383@stu.yzu.edu.cn (Z.H.); 2 Jiangsu Co-Innovation Center for Modern Production Technology of Grain Crops, Jiangsu Key Laboratory of Crop Genetics and Physiology, Yangzhou University, Yangzhou 225000, China; 3 Yangzhou Modern Seed Innovation Institute, Gaoyou 225000, China

**Keywords:** genome-wide identification, *Triticum aestivum* L., sugar transport proteins, gene expression, fusarium head blight

## Abstract

Fusarium head blight (FHB) negatively affects wheat yield and quality worldwide. As wheat varieties differ in terms of their resistance to FHB, the identification of FHB-resistant genes is of great importance for the genetic improvement for FHB resistance in wheat breeding. Although sugar transporter proteins (STPs) play vital roles in plant–pathogen interactions, the functions of *STP* genes in wheat FHB resistance remain poorly understood. In this study, bioinformatics analyses were conducted to identify novel STP genes and characterize their expression profiles in wheat. We confirmed the presence of the 81 TaSTP genes previously reported and identified one additional member, designated as *TaSTP6-2D*. Based on RNA-seq profiles, 50 TaSTP genes that showed differential expression under biotic or abiotic stress were selected to explore the potential function in the resistance to Fusarium head blight. RT-qPCR analysis revealed that 11 TaSTP genes (*TaSTP1-2D*, *TaSTP3-2A*, *TaSTP3-2B*, *TaSTP6-2A*, *TaSTP6-2B*, *TaSTP13-4B*, *TaSTP13-4D*, *TaSTP19-4A*, *TaSTP26-5A*, *TaSTP28-3A* and *TaSTP28-3D*) were differential expressed following the treatment with chitin, *Fusarium graminearum* or deoxynivalenol. Among them, *TaSTP26-5A* showed a 28-fold upregulation to chitin in “Yangmai 158” compared to a 6-fold change in “Fielder”. These findings establish a foundation for understanding the function of TaSTP genes in FHB resistance and provide potential genetic targets for improving disease resistance in wheat.

## 1. Introduction

Wheat (*Triticum aestivum* L.) is one of the world’s most crucial cereal crops, serving as a primary staple food for over a third of the global population. The expanding global population requires an increase in wheat production. However, wheat growth is continuously threatened by various biotic and abiotic stresses. In particular, Fusarium head blight (FHB) caused by the *Fusarium graminearum* complex species is a devastating disease in wheat worldwide. It not only severely reduces the yield and quality of grains but also produces mycotoxins such as deoxynivalenol (DON) and zearalenone (ZEN), which pose serious threats to human and animal health [1,2,3]. In China, the FHB epidemic area has been expanding since 2000 due to a combination of factors, including climate change, crop rotation practices, improper straw management, and the increasing fungicide resistance of Fusarium pathogens [4,5]. Among all control strategies, developing and applying FHB resistant varieties is considered the most effective and environmentally friendly approach for managing the disease [4,5].

FHB resistance is a typical quantitative trait controlled by multiple genes and highly influenced by environmental conditions. Although no immune wheat varieties have been found, significant differences in resistance levels exist, highlighting the potential for genetic improvement. Identifying the quantitative trait locus (QTL) or genes associated with FHB resistance is crucial for improving the efficiency in wheat breeding. Since the first major QTL associated with FHB resistance was characterized on chromosome 3BS [6], over 600 QTL have been reported across all chromosomes in wheat, while only a few of QTL have been used for marker-assisted breeding [7,8]. *Fhb1* is the most effective QTL and widely used in wheat breeding, and its candidate gene has been isolated and functionally validated [9,10,11]. Another notable QTL, *Fhb7*, which encodes a glutathione S-transferase that detoxifies trichothecenes, was cloned from *Thinopyrum elongatum* [12]. However, the large and complex hexaploid wheat genome makes map-based cloning extremely challenging [13,14]. Consequently, there remains an urgent need to identify and characterize novel genes contributing to FHB resistance through alternative approaches

Sugar serves as an essential carbon and energy source for both plants and pathogens, playing a vital role in plant growth, development, and response to biotic and abiotic stresses [15,16,17]. Pathogen proliferation depends on successful sugar acquisition from the host, making sugar transport a key battleground in plant-pathogen interactions [18,19,20]. Plant sugar transporter proteins, including monosaccharide transporter proteins (MSTs), sucrose transporter proteins (SUTs), and sugar efflux transporter proteins (SWEETs), are central mediators in this process [19,20]. MSTs can be further subdivided into six distinct subfamilies: glucose transporter, hexose transporter (STP), ERD six-like transporter, plastid glucose transporter, vesicular glucose transporter, and tonoplast membrane transporter proteins. Of particular interest are the function of STP, which are primarily involved in sugar transport during the plants-pathogens interactions [21,22]. Other sugar transport proteins such as OsSWEET4, AtSWEET11, AtSWEET12, and AtSWEET15 influence seed development; AtSWEET1, AtSWEET5, AtSWEET8, AtSWEET13, OsSWEET11, OsSWEET12, and OsSWEET22 regulate pollen development; and OsSWEET15 and AtSWEET15 affect leaf senescence [23,24,25,26]. STPs, which are affected by biotic and abiotic stresses, are increasingly reported to be involved in sugar competition, partitioning, and translocation in plant–pathogen interactions. For instance, in *Arabidopsis thaliana*, *AtSTP1*, *AtSTP3*, *AtSTP4*, and *AtSTP13* are induced by the bacterial exotoxin flg22 or infection by *Pseudomonas syringae* [27,28,29]. Overexpression of *AtSTP13* in *Arabidopsis* enhances resistance to *Botrytis cinerea* [30]. *AtSTP13* is significantly induced by *P. syringae* and is activated by phosphorylation interactions between *AtSTP13* and *P. syringae*, which enhances its hexose uptake activity and competes with bacteria for the de-competition of monosaccharides [31,32]. Similarly, *AtSTP4* has been shown to play a role in synergistic expression in the interactions between *P. albicans* and its host [33]. Studies on *TaSTP* in wheat have demonstrated that mutations in *TaSTP13* (*lr67*) can result in resistance to a broad spectrum of biotrophic pathogens [34,35]. The expression of *TaSTP3* was regulated by transcription factors *TaWRKY61* and *TaWRKY82*, and the overexpression of *TaSTP3* promotes the growth and development of stripe rust, thus, *TaSTP3* increases the susceptibility to stripe rust in wheat [36]. Similarly, the upregulated expression of *TaSTP6* promoted the infection of wheat with stripe rust [37].

Despite the identification of 81 TaSTP genes in wheat and their established roles in seedling abiotic stress [38], their specific functions and mechanisms in the response to the hemibiotrophic fungus *F. graminearum* and DON toxicity remain almost entirely unknown, representing a critical knowledge gap. To address this gap and identify novel players in FHB resistance, this study conducted a comprehensive genome-wide analysis of TaSTP gene family using the genome database (IWGSCv2.1) to investigate their phylogenetic relationships, chromosomal locations, and evolutionary history. Furthermore, we analyzed their expression profiles using RNA-seq data across tissues and in response to variousbiotic or abiotic stress. Most importantly, to directly investigate their role in FHB resistance, we systematically characterized the expression patterns of selected TaSTP genes in FHB-resistant and susceptible wheat varieties following treatment with chitin, *F. graminearum* and DON treatments using real-time quantitative polymerase chain reaction (RT-qPCR). Our work provides crucial foundational insights and identifies key candidate TaSTP genes for future functional studies aimed at elucidating their roles in FHB resistance and DON detoxification, ultimately contributing to the development of resistant wheat varieties.

## 2. Results

### 2.1. Identification and Phylogenetic Analysis of TaSTP Genes in Wheat

The conserved domains of sugar transporter proteins (PF00083) were searched against the WheatOmics 1.0 database, yielding 307 protein sequences. Additionally, *STPs* from model plants—14 AtSTPs from *Arabidopsis thaliana* and 28 OsSTPs from rice (*Oryza sativa*)—were used as queries in a BLAST search to identify wheat STPs. Through comprehensive comparison, 81 TaSTP*s* (including alternatively spliced variants) were identified. After integrating the sequences obtained from both methods and removing duplicates, 312 sequences were retained. A phylogenetic tree was constructed using the protein sequences of 312 putative TaSTPs, 14 AtSTPs, and 28 OsSTPs (Figure S1). The phylogenetic tree was divided into four major subgroups, with Group IV containing *AtSTP1*–*AtSTP14* from *Arabidopsis*, *OsSTP1*–*OsSTP28* from rice, and 87 genes from wheat. To further refine the analysis, the 87 genes from wheat in Group IV were subjected to conserved domain detection using the NCBI Batch CD-Search Tool (https://www.ncbi.nlm.nih.gov/Structure/bwrpsb/bwrpsb.cgi (accessed on 1 February 2024)). Five genes lacking completely conserved domains (MFS-STP) were removed. Consequently, a final set of 82 genes with intact conserved domains was retained for subsequent analysis (Table S1). These sugar transporter TaSTP family members exhibited the coding sequence (CDS) lengths ranging from 1080 to 1848 bp, protein length ranging from 359 to 615 amino acids(aa), molecular weights ranging from 39.55 to 67.04 kDa, and isoelectric points (pI) ranging from 8.42 to 10.5 (Table S2).

**Figure 1:**
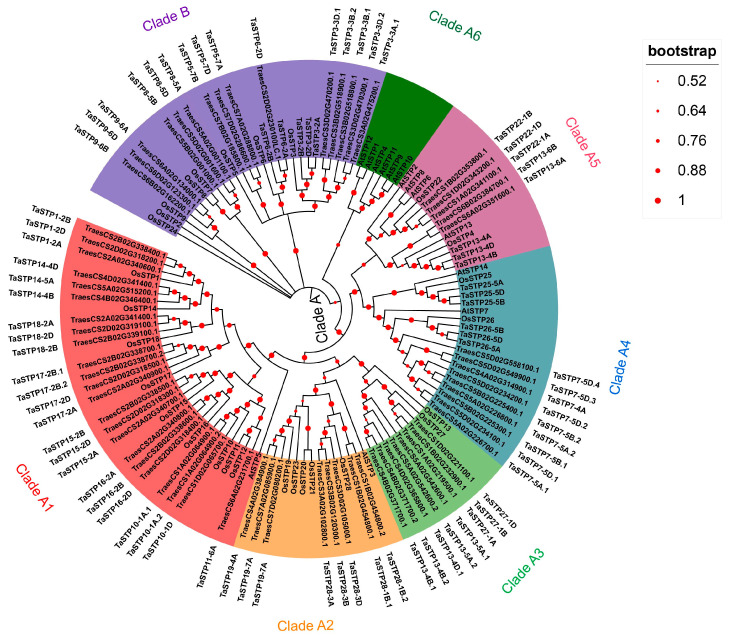
Phylogenetic tree of the STPs from wheat, rice and *Arabidopsis thaliana*. An evolutionary tree was formed by the phylogenetic relationships of 82 predicted TaSTPs, Rice (OsSTP1-OsSTP28) and *Arabidopsis thaliana* (AtSTP1-AtSTP14) proteins with 1000 bootstrap replicates by MEGA-X. There are genes in wheat in the same branch as that of rice and *Arabidopsis thaliana* are marked with the same color. The outermost black portion of the text denotes the gene nomenclature for the TaSTP genes in this study. The tree is divided into two main clades, A and B, indicated by the colored bars. Main clades are further subdivided into subclades (e.g., A1, A2), which are highlighted by different background shades.

The phylogenetic tree in Figure 1 divides wheat STPs into two main clades (A and B). Within Clade A, we identified six robust subclades: Subclade A1 included AtSTP5 from Arabidopsis, nine STPs from rice (OsSTP1, OsSTP10, OsSTP11, OsSTP12, OsSTP14, OsSTP15, OsSTP16, OsSTP17, and OsSTP18), and 23 TaSTPs in wheat. Subclade A2 consisted of AtSTP3 from Arabidopsis; OsSTP19, OsSTP20, OsSTP21, OsSTP23, and OsSTP28 from rice; and eight TaSTPs in wheat. Subclade A3 contained two rice STPs (OsSTP13 and OsSTP27) and eight wheat TaSTPs. Subclade A4 included AtSTP7, AtSTP14, OsSTP25, OsSTP26, and fifteen wheat TaSTPs. Subclade A5 contained four *Arabidopsis* STPs (AtSTP2, AtSTP6, AtSTP8, and AtSTP13), two rice STPs (OsSTP4 and OsSTP22) and eight wheat TaSTPs. Subclade A6 contained six *Arabidopsis* STPs (AtSTP1, AtSTP4, AtSTP9, AtSTP10, AtSTP11, and AtSTP12). Clade B included seven rice STPs and 20 wheat TaSTPs. The nomenclature of previously unnamed TaSTP genes was standardized in this study (Table S3).

Notably, the phylogenetic clustering revealed that several TaSTP genes grouped within clades known to contain members involved in plant stress responses. For instance, the clade containing *TaSTP13-4A*, *TaSTP13-4B*, and *TaSTP13-4D* also includes *Arabidopsis AtSTP13* (Figure 1), which has been implicated in biotic stress responses [30]. This evolutionary conservation suggests a potential parallel role for these wheat STP genes, such as *TaSTP13-4A*, *TaSTP13-4B*, and *TaSTP13-4D*, in the defense response against pathogens such as *F. graminearum*. The expression patterns of these phylogenetically informed candidate genes in response to FHB-related stresses were further investigated.

**Figure 2:**
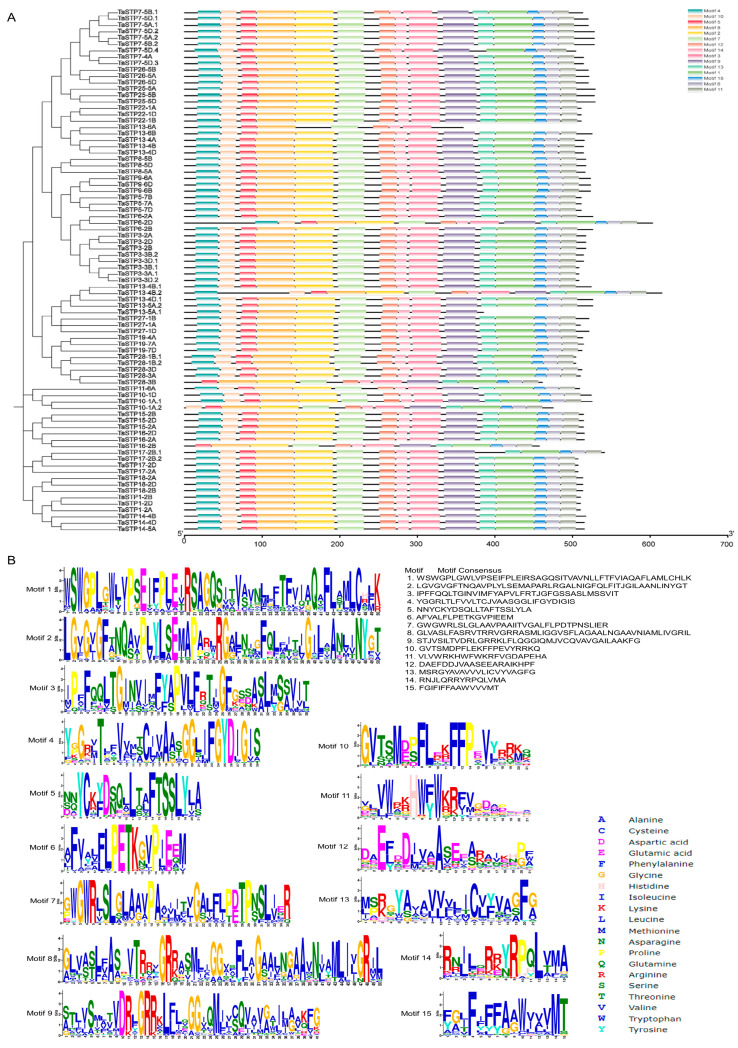
MEME motif and conservative domains results of the predicted TaSTPs. (**A**) MEME motif search by MEME suite 5.5.7 and conservative domains search by CDD Tools of NCBI. The non-conserved sequences are shown by black lines, and the different motifs are represented by different colored boxes numbered at the center of the box and bottom of the figure. The lengths of the motifs in each protein are proportional. The phylogenetic tree is the same as that in Figure 1. In addition, the non-conserved sequences of conservative domains are shown by gray boxes, and the same conservative domains are represented by same colored boxes of the figure. (**B**) Motif logos and the amino acid compositions of the TaSTPs. The *x*-axis represents the amino acid type and position. The *y*-axis shows the overall height of the amino acid stacks, which indicates the sequence conservation at a given position, while the height of the individual symbols within a stack indicates the relative frequency of a nucleotide base at that position.

**Figure 3:**
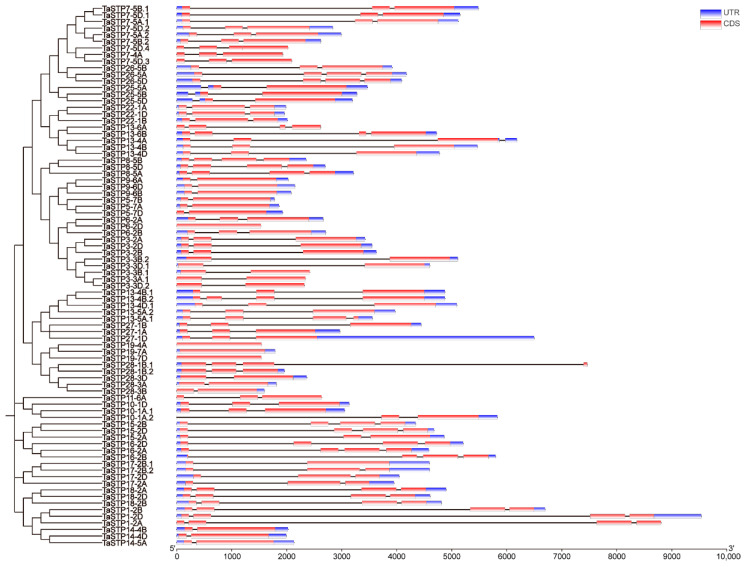
The structure of TaSTP genes family members. The exon–intron structures of the 82 TaSTP genes were generated by comparing the coding sequences optimized using TBtools (version 2.345). The phylogenetic tree is the same as that in Figure 1.

**Figure 4:**
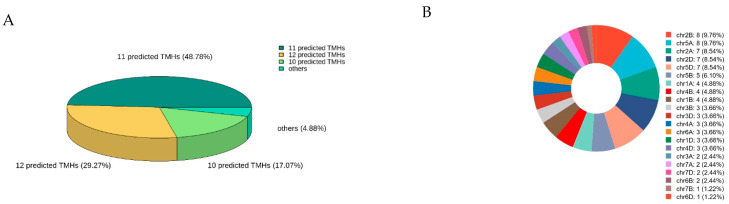
Distribution of the TaSTP genes on the wheat chromosomes and 3D pie chart of predicted TMHs with TMHMM2.0. (**A**) 3D pie chart of predicted TMHs in TaSTP protein. The different colors in the graph represent different categories, and the percentages represent the categories percentage calculated with 82 TaSTPs. (**B**) TaSTP genes were differentially distributed on 21 wheat chromosomes.

The MEME Suite 5.5.7 software was used to analyze the 82 identified TaSTPs in wheat. A total of fifteen motifs were identified, with lengths ranging from 15 to 50 amino acids. Among these, Motif2, Motif3, Motif5, Motif7, Motif8, Motif12, and Motif14 were highly conserved across the members of the wheat TaSTP family (Figure 2). Further analysis of the conserved STP structures in wheat revealed that the major facilitator superfamily (MFS), D-xylose transporter superfamily (XylE), and arabinose proton symporter (AraE) were highly conserved in all 82 TaSTP*s* (Figure S2). The TaSTP gene structures commonly had two, three, or four exons divided by one, two, or three introns, except for TaSTP*19-4A*, *TaSTP19-7A*, and *TaSTP19-7D*, which lacked introns. In general, TaSTP genes within the same phylogenetic subgroup exhibited similar gene structures (Figure 3). An online prediction tool that allows for the subcellular localization prediction of the 82 TaSTP*s* predicted that 25 genes were localized in the vacuolar membrane, while 57 genes were localized to the plasma membrane (Table S4). Transmembrane helix (TMH) prediction using TMHMM 2.0 revealed that 6 to 13 TMHs were present in wheat. Specifically, 48.78% of the TaSTP*s* contained 11 TMHs, 29.27% contained 12 TMHs, and 17.07% contained 10 TMHs (Figure 4A and Figure S3 and Table S5).

### 2.2. Genome Distribution of Wheat TaSTP Genes

A total of 82 TaSTPs were identified in wheat, a number notably higher than the 14 *AtSTPs* in Arabidopsis and 28 *OsSTPs* in rice. These TaSTP*s* were distributed across all 21 chromosomes of wheat. The chromosomal pattern of the 82 TaSTP genes was uneven. The highest density of genes was observed on Chr 2B and 3A, each containing 8 TaSTP genes (accounting for 9.76% of total each). This was followed by Chr 2A, 2D, and 4D, each harboring 7 genes (8.54% each). Conversely, Chr 7B and 6D contained only a single TaSTP gene each (1.22% each), indicating the lowest gene density on these chromosomes. This heterogeneous distribution pattern suggests possible chromosome-specific evolutionary pressures or functional specializations within the STP gene family in wheat (Figure 4B).

The uneven distribution of TaSTP genes across the wheat subgenomes is likely a attributed to evolutionary processes such as tandem duplication events, which may lead to local gene clusters, and/or larger-scale segmental duplications followed by selective gene loss or retention over time. This non-random distribution may also reflect functional specialization, implying that chromosomes rich in TaSTP genes could be hotspots for regulating sugar transport during development and stress responses. Figure S4 illustrates the chromosomal distribution of TaSTP family members. Among them, 18 members are located on the short arms (p arms), whereas 64 members are located on the long arms (q arms) (Figure S4 and Table S6). In addition, 46 members (including *TaSTP8-5A*, *TaSTP8-5B*, and *TaSTP8-5D*) are positioned near the telomeres, and 36 members (including *TaSTP3-2A*, *TaSTP3-2B*, and *TaSTP3-2D*) are located near the centromeres (Figure S4 and Table S6).

### 2.3. Expression Profiles of TaSTP Genes

Publicly available RNA-seq data was used to analyze the expression profiles of TaSTP genes across different tissues and developmental stages of wheat, as well as under abiotic and biotic stressors and phytohormone treatments. Since four genes with identical transcripts were excluded, only the expression profiles of the remaining 77 TaSTP genes are presented in the following sections. Expression data were obtained from public databases and are represented as normalized mean values. This analysis served as preliminary candidate gene screening based on expression trends and was not intended for drawing definitive statistical conclusions.

#### 2.3.1. Gene Expression in Wheat Tissues

The expression of 77 TaSTP genes (excluding splice variants) were varied across root, stem, leaf, spike, and grain in wheat (Figure S5). The expression levels of *TaSTP8-5B*, *TaSTP28-3D*, *TaSTP14-4B*, *TaSTP18-2B*, *TaSTP19-7D*, *TaSTP19-4A*, *TaSTP13-6B*, *TaSTP7-5A.1*, *TaSTP9-6B*, and *TaSTP28-3A* were very low or undetectable in all of the examined root, stem, leaf, spike, and grain tissues, whereas *TaSTP25-5B*, *TaSTP6-2D*, *TaSTP13-4A*, *TaSTP26-5A*, *TaSTP6-2A*, *TaSTP16-2D*, *TaSTP26-5D*, *TaSTP25-5A*, *TaSTP25-5D*, *TaSTP3-2B*, *TaSTP3-2D*, *TaSTP13-4D*, *TaSTP6-2B*, *TaSTP3-2A*, and *TaSTP13-4B* exhibited consistently high expression across all tested tissues, with particularly strong expression in roots. *TaSTP26-5B* was predominantly expressed in the grains. *TaSTP27-5A.1*, *TaSTP7-5A.2*, *TaSTP17-2A*, *TaSTP17-2D*, *TaSTP17-2B.1*, *TaSTP7-5B.2*, and *TaSTP7-5D.2* showed higher expression in stems. *TaSTP13-4B.1* and *TaSTP13-4D.1* exhibited elevated expression in spikes (Figure S5). These findings suggest that TaSTP genes play diverse functional roles in different wheat tissues.

#### 2.3.2. Gene Expression in Wheat Developmental Stages

The expression profiles of 77 TaSTP genes across wheat developmental stages including seedling, three-leaf, tillering, flag-leaf, spike, anthesis, milk grain, and ripening are shown in Figures S6–S13. Several strong, stage-specific expression patterns were identified:

Root-Preferential Expression: A core set of genes, including *TaSTP3-2A*, *TaSTP3-2B*, *TaSTP3-2D*, and *TaSTP25-5A*, displayed consistently high expression in root tissues across multiple stages (e.g., seedling, three-leaf, flag leaf, and spike stages; Figures S6, S7, S9 and S10), suggesting a conserved role in root development and nutrient acquisition.

Photosynthetic and Vascular Tissue Expression: During later vegetative stages, genes such as *TaSTP13-4D* showed remarkably high expression in the flag leaf blade (TPM > 100 at anthesis, Figure S11), implying a potential role in sugar loading or transport within photosynthetic tissues. Similarly, *TaSTP17-2D* was highly expressed in the peduncle (Figure S12), a key vascular structure.

Reproductive Organ Expression: Several genes exhibited specific expressions in reproductive organs. For instance, *TaSTP9-6D* showed its highest expression in anthers, while *TaSTP13-5A.1* was highly expressed in the stigma and ovaries during anthesis (Figure S11). This implicates them in sugar partitioning critical for pollination and early grain development.

Grain and Senescence-Associated Expression: Although most TaSTP genes showed low expression in ripening grains, *TaSTP25-5A* was a notable exception, with high expression in milk grains (Figure S12), suggesting a role in sugar import during grain filling. Furthermore, genes like *TaSTP3-2B*, *TaSTP3-2D*, and *TaSTP13-4D* maintained high expression in flag leaves during ripening (Figure S13), potentially facilitating carbon remobilization from senescing leaves to the developing grains.

This developmental expression atlas served as a foundation for candidate gene selection. We specifically prioritized genes with high expression in reproductive tissues (e.g., spikes and flowers), as these are the primary infection sites for *F. graminearum.* For instance, *TaSTP22-1A*, *TaSTP22-1B*, *TaSTP22-1D*, *TaSTP25-5D*, and *TaSTP26-5A* were ranked among the top 20 most highly expressed genes in these critical organs (Supplementary Figure S14). Their pronounced upregulation during reproductive development stages underscores their potential importance in sugar allocation to the spike and grains. Given that the spike is the primary infection site for *F. graminearum*, these genes were selected as high-priority candidates for further functional characterization of their roles in the FHB response

#### 2.3.3. Gene Expression Under Abiotic, Biotic, and Hormonal Stress Conditions

The expression profiles of 77 TaSTP genes under abiotic (low temperature, drought, heat, and salt) (Figure S15) and biotic stress (stripe rust, powdery mildew, and *F. graminearum*) were analyzed using an online database (Figures S16 and S17). Additionally, the expression profiles in response to phytohormone treatments—including gibberellic acid (GA), jasmonic acid (JA), abscisic acid (ABA), salicylic acid (SA), and 6-benzylaminopurine (6-BA)—were also retrieved from the database (Figure S18).

To identify TaSTP genes potentially involved in broad-spectrum stress responses, we analyzed their expression profiles under various abiotic, biotic, and hormonal treatments using public database resources. This bioinformatic screening aimed to prioritize candidates based on their responsiveness, providing a foundation for subsequent functional investigation in FHB resistance. Notably, several genes exhibited coordinated expression patterns across multiple stress types, suggesting they may function as integrative hubs in stress signaling networks. For instance, the *TaSTP3-2A*, *TaSTP3-2B*, *TaSTP3-2D*, *TaSTP25-5A*, *TaSTP25-5B*, and *TaSTP25-5D* genes were strongly induced by diverse abiotic stresses (e.g., drought, salt) (Figure S15), which often activate ABA-dependent signaling pathways. This consistent upregulation suggests a potential role for these genes in ABA-mediated abiotic stress adaptation. The same group of genes (*TaSTP3-2A*, *TaSTP3-2B*, *TaSTP3-2D*, *TaSTP25-5A*, *TaSTP25-5B*) was also highly induced by biotic stresses such as powdery mildew and stripe rust (Figure S16), implying a potential convergence point of abiotic and biotic signaling pathways in regulating these transporters.

Most importantly, in the context of this study, analysis of *Fhb1* near-isogenic lines revealed that key candidates, including *TaSTP3-2A*, *TaSTP3-2B*, *TaSTP3-2D*, *TaSTP13-4A*, *TaSTP13-4B*, *TaSTP13-4D*, *TaSTP28-3A*, and *TaSTP28-3D*, were significantly upregulated as early as 12 h post-inoculation with *F. graminearum* or DON treatment, independent of the *Fhb1* status (Figure S17). This rapid and strong induction upon direct exposure to the pathogen and its key toxin strongly supports their potential direct involvement in the wheat-FHB interaction, making them high-priority candidates for validation. Furthermore, interrogation of expression data under phytohormone treatments (Figure S18) provided initial insights into potential regulatory mechanisms. The downregulation of *TaSTP3* homoeologs by ABA and SA aligns with the complex crosstalk between stress hormones, while their induction by the cytokinin 6-BA suggests a potential link to growth-defense trade-offs. Although these database-derived patterns are preliminary, they generate valuable hypotheses regarding the hormonal control of TaSTP genes for future experimental validation. A conceptual model integrating these expression patterns and their potential regulatory pathways is proposed in Supplementary Figure S19. This model summarizes how key TaSTP candidates may function as hubs at the convergence of multiple stress signaling networks to potentially influence FHB resistance (Figure S19).

### 2.4. FHB Resistance of the Fielder and Yangmai 158

**Figure 5:**
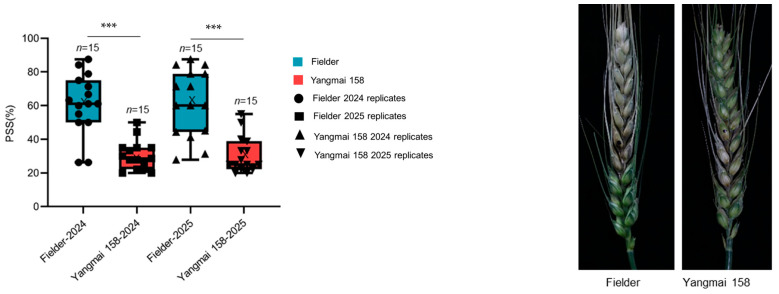
Phenotypic characterization of Fielder and Yangmai 158 after infection with *F. graminearum* at 21 days. For box plots, the year and cultivars in which FHB was evaluated are shown on the *x* axis, the percentage of symptomatic spikelets (PSS) is shown on the y axis, boxes indicate the 20th–85th percentile, whiskers indicate the full data range, center lines indicate the median, crosses indicate the mean (n  =  15). Significant differences between varieties are indicated by * above the box plot, ns *p* > 0.05; * *p* < 0.05; ** *p* < 0.01; *** *p* < 0.001 (*p* values are from an unpaired two-sided Student’s *t* test). Right, inoculated spikes from Fielder and Yangmai 158.

The FHB resistance levels of the wheat cultivars “Fielder” and “Yangmai 158” were rigorously evaluated through field experiments conducted over two consecutive growing seasons (2024 and 2025). Clear phenotypic differences were observed between the two cultivars following inoculation with *F. graminearum*. Quantitative assessment of disease severity, measured by the percentage of symptomatic spikelets (PSS), consistently and conclusively identified “Yangmai 158” as a highly resistant genotype. In both years, “Fielder” exhibited high susceptibility, with mean PSS values of 61.1% (2024) and 61.7% (2025). In contrast, “Yangmai 158” demonstrated significantly stronger resistance, with mean PSS values of only 30.5% (2024) and 30.9% (2025). The highly statistically significant, with an adjusted *p*-value < 0.001 for both seasons, confirming the extreme reproducibility and reliability of the phenotypic data (Figure 5 and Table S7). This consistent contrast in FHB resistance—where “Yangmai 158” showed approximately 50% lower disease severity than “Fielder”—provided a reliable experimental system for subsequent investigation into the molecular mechanisms of resistance. The pronounced difference in their susceptibility makes this cultivar pair well-suited for examining differential gene expression and physiological responses associated with FHB resistance in wheat.

### 2.5. Validation of the Expression of Wheat TaSTP Genes by RT-qPCR

After a comprehensive analysis of the TaSTP genes expression across different tissues and developmental stages of wheat, abiotic and biotic stress conditions, and phytohormone treatments, a set of 50 TaSTP genes was selected to explore the potential function in the resistance to Fusarium head blight (Table S8). Their expressions profiles were analyzed by RT-qPCR in response to the treatments of chitin, *F. graminearum* and DON, respectively, in wheat varieties “Fielder” (susceptible) and “Yangmai 158” (resistant).

#### 2.5.1. Chitin Treatment

**Figure 6:**
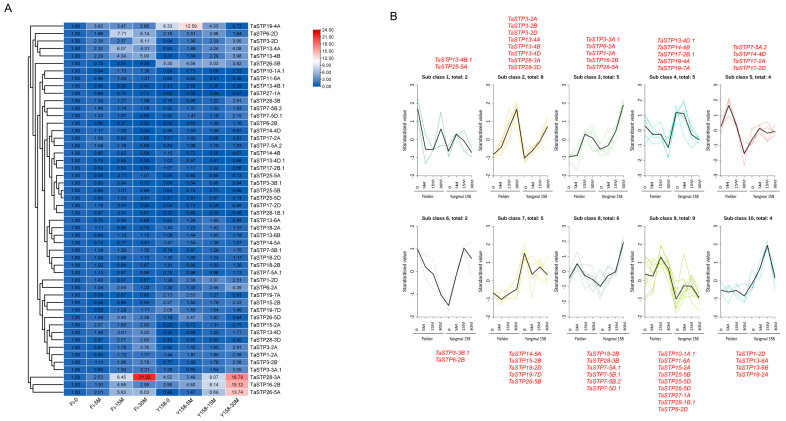
Expression profiles of TaSTP genes after chitin treatment. (**A**) Heatmap of TaSTP genes. The horizontal axis shows the sample name, and the vertical axis shows TaSTP genes, and the different colors are the values obtained after standardization in horizontal rows of the relative expression level. Red represents high expression; blue represents low expression. The numbers in the heatmap represent the original relative expression levels of the Fi-0 of samples as a control. The same color represents consistent expression patterns in the sample. (**B**) Differential TaSTP genes K-means diagram. The x-coordinate represents the sample and the y-coordinate represents the relative expression of the standardized TaSTP genes. As indicated by the red font, the genes shown beside the sub class represent its constituent members.

RT-qPCR analysis of 50 TaSTP genes under chitin treatment was performed, using untreated samples of “Fielder” (Fi-0) as the control. Gene expression was assessed at 5, 15, and 30 min after chitin treatment in both the “Fielder” and “Yangmai 158” wheat. The heatmap displays the expression patterns of TaSTP genes in both “Fielder” and “Yangmai 158” after chitin treatment (Figure 6A). Based on differential gene expression analysis (Table 1 and Table S9; Figure S20), we found that 26 genes (15 genes upregulated and 11 genes downregulated) were significantly differentially expressed (adjusted *p* < 0.05) at 5 min after chitin treatment compared to the control in “Fielder”, while 27 genes (24 TaSTP genes upregulated and three TaSTP genes downregulated) responded in “Yangmai 158”. At 15 min post-treatment, 30 TaSTP genes (21 genes upregulated, nine genes downregulated) responded in “Fielder” compared to control, and 34 TaSTP genes (28 genes upregulated, six genes downregulated) in “Yangmai 158” (Figure S20). At 30 min post-treatment, 33 TaSTP genes responded in “Fielder” (eighteen genes upregulated, fifteen genes downregulated) and 35 genes responded in “Yangmai 158” (26 genes upregulated, nine genes downregulated) (Figure S20).

Notably, a subset of genes exhibited consistent upregulation at all time points (5 min, 15 min, and 30 min). In “Fielder”, ten TaSTP genes (*TaSTP6-2D*, *TaSTP7-5B.1*, *TaSTP13-4A*, *TaSTP13-4B*, *TaSTP13-4D*, *TaSTP15-2A*, *TaSTP16-2B*, *TaSTP19-4A*, *TaSTP26-5A*, and *TaSTP28-3A*) were significantly induced by chitin treatment, among which *TaSTP28-3A* exhibited a 21-fold upregulation at 15 min. In “Yangmai 158”, 18 genes (*TaSTP1-2D*, *TaSTP3-2A*, *TaSTP3-2B*, *TaSTP3-2D*, *TaSTP3-3B.1*, *TaSTP6-2B*, *TaSTP7-5B.1*, *TaSTP7-5B.2*, *TaSTP7-5D.1*, *TaSTP13-4A*, *TaSTP13-4B*, *TaSTP13-4D*, *TaSTP15-2A*, *TaSTP25-5D*, *TaSTP26-5A*, *TaSTP27-1A*, *TaSTP28-1B.1*, and *TaSTP28-3B*) were induced and upregulated, with *TaSTP26-5A* showing a 28-fold upregulation at 30 min (Table 1 and Figure S20).

The K-means clustering analysis (Figure 6B) further elucidated the distinct transcriptional reprogramming dynamics between the susceptible and resistant varieties in response to chitin treatment. Subclass 3 (e.g., *TaSTP26-5A*) comprised genes that were rapidly and transiently upregulated in both varieties at 5 min post-treatment, but the induction was markedly stronger and more sustained in “Yangmai 158” than in “Fielder”. This pattern suggests a more robust and sensitive perception of the chitin signal in the resistant variety. Subclass 8 (e.g., *TaSTP28-3B*) contained genes that were specifically upregulated in “Yangmai 158” across multiple time points, showing little to no response in “Fielder”. This cluster represents a set of responses that may be uniquely deployed in the resistant genotype upon pathogen-associated molecular pattern (PAMP) perception. Subclass 6 (e.g., *TaSTP6-2B*) included genes that were downregulated in “Fielder” but remained unchanged or were slightly induced in “Yangmai 158”. This opposing regulation highlights a potential divergence in the signaling networks following chitin recognition between the two varieties.

In summary, the overall response in the resistant cultivar “Yangmai 158” was characterized by a stronger, more timely, and specific upregulation of key TaSTP genes. In contrast, the susceptible cultivar “Fielder” exhibited a weaker and less coordinated response, often featuring inappropriate downregulation of certain family members. This differential regulation of sugar transporters at the early immune signaling stage could contribute to the disparity in downstream disease resistance outcomes.

To further confirm the role of TaSTP genes in wheat immunity against FHB, 35 representative genes (including both chitin-responsive and non-responsive genes) were selected from Table 1 for subsequent analysis of their expression levels under *F. graminearum* infection.

#### 2.5.2. *F. graminearum* Infection

**Figure 7:**
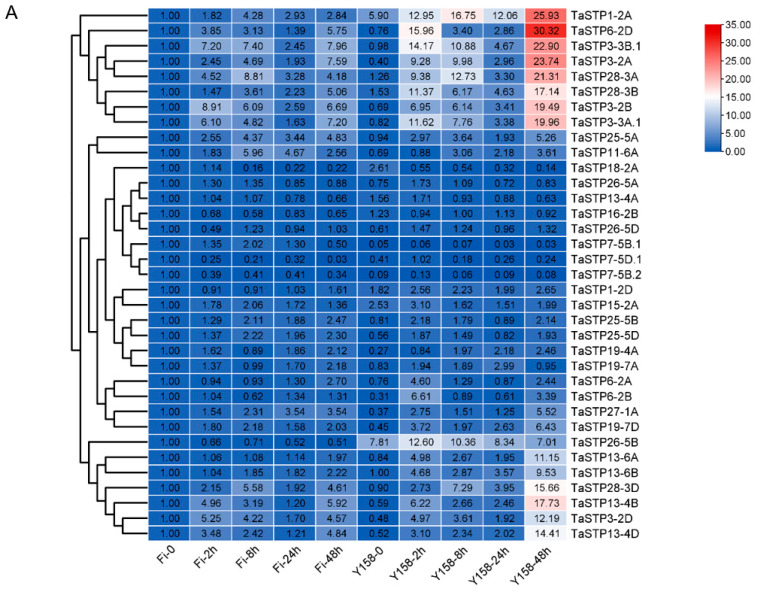

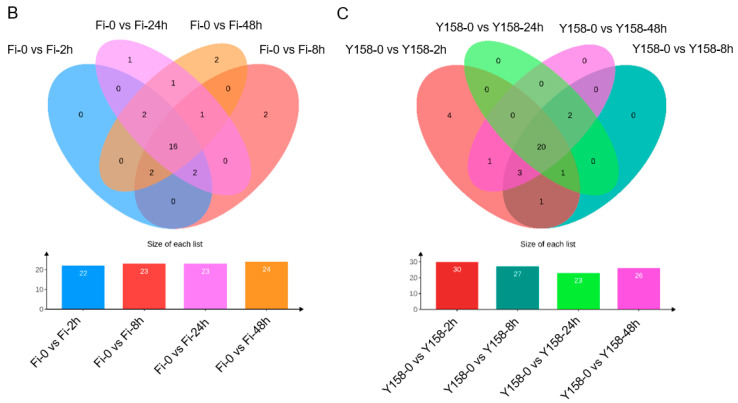
Expression profiles of TaSTP genes infested with *F. graminearum.* (**A**) The heatmap of TaSTP genes infested with *F.graminearum*. The horizontal axis shows the sample name, and the vertical axis shows TaSTP genes, and the different colors are the values obtained after standardization in horizontal rows of the relative expression level. Red represents high expression; blue represents low expression. The numbers in the heatmap represent the original relative expression levels of the Fi-0 of samples as a control. The same color represents consistent expression patterns in the sample. (**B**) Venn diagram of TaSTP genes upregulated in Fielder. (**C**) Venn diagram of TaSTP genes upregulated in Yangmai 158. The non-overlapping area of the Venn diagram represents the TaSTP genes specific to the subgroup comparison, and the overlapping area represents the TaSTP genes common to the several subgroup comparisons. The bar chart depicts the size of TaSTP genes within each subgroup of the Venn diagram.

The expression of 35 TaSTP genes (from Table 1) was further analyzed by RT-qPCR after inoculation of *F. graminearum* in FHB susceptible variety “Fielder” and resistant variety “Yangmai 158”. Untreated “Fielder” (Fi-0) served as the control, gene expression was assessed at 2, 8, 24, and 48 h post-inoculation (hpi). The heatmap illustrates the expression patterns of TaSTP genes in wheat cultivars “Fielder” and “Yangmai 158” following *F. graminearum* infection, highlighting pathogen-induced upregulated genes (Figure 7A).

In “Fielder”, 22 TaSTP genes were upregulated at 2 hpi, 23 at 8 hpi, 23 at 24 hpi, and 24 at 48 hpi (Figure 7B; Table 2 and Table S10). Notably, sixteen genes (*TaSTP1-2A*, *TaSTP3-2A*, *TaSTP3-2B*, *TaSTP3-2D*, *TaSTP3-3B.1*, *TaSTP13-4B*, *TaSTP13-4D*, *TaSTP15-2A*, *TaSTP19-7D*, *TaSTP25-5A*, *TaSTP25-5B*, *TaSTP25-5D*, *TaSTP27-1A*, *TaSTP28-3A*, *TaSTP28-3B*, and *TaSTP28-3D*) exhibited persistent upregulation (2–48 hpi), with *TaSTP3-2B* showed the earliest induction, with a 9-fold upregulated at 2 hpi (Figure 7B and Table 2).

In “Yangmai 158”, 30 TaSTP genes upregulated at 2 hpi, 27 genes upregulated at 8 hpi, 23 genes upregulated at 24 hpi, 26 genes upregulated at 48 hpi. And twenty genes (*TaSTP1-2A*, *TaSTP3-2B*, *TaSTP3-2D*, *TaSTP3-3A.1*, *TaSTP3-3B.1*, *TaSTP6-2B*, *TaSTP6-2D*, *TaSTP13-4B*, *TaSTP13-4D*, *TaSTP13-6A*, *TaSTP13-6B*, *TaSTP19-4A*, *TaSTP19-7D*, *TaSTP25-5A*, *TaSTP25-5D*, *TaSTP26-5D*, *TaSTP27-1A*, *TaSTP28-3A*, *TaSTP28-3B*, and *TaSTP28-3D*) demonstrated sustained upregulation (2–48 hpi). Of particular note, *TaSTP3-2A* showing a 25-fold upregulation at 8 hpi, both *TaSTP6-2B* and *TaSTP6-2D* showing 21-fold upregulation at 2 hpi. (Figure 7C and Table 2). Analysis of downregulated TaSTP genes is presented in Table S10.

#### 2.5.3. DON Treatment

DON is a critical virulence factor produced by *F. graminearum* that facilitates the spread of disease symptoms within wheat spikes. To investigate the involvement of TaSTP genes in late-stage wheat-*F. graminearum* interactions, 22 genes were selected from Table 2 based on distinct response profiles: 11 TaSTP genes (*TaSTP3-2A*, *TaSTP3-2B*, *TaSTP3-2D*, *TaSTP6-2D*, *TaSTP13-4B*, *TaSTP13-4D*, *TaSTP15-2A*, *TaSTP19-7D*,*TaSTP27-1A*, *TaSTP28-3A*, and *TaSTP28-3D*) that were upregulated in both cultivars 2–48 hpi, 10 genes (*TaSTP1-2D*, *TaSTP6-2A*, *TaSTP6-2B*, *TaSTP13-6A*, *TaSTP13-6B*, *TaSTP19-4A*, *TaSTP19-7A*, *TaSTP26-5A*, *TaSTP26-5B*, *TaSTP26*-*5D*) that exhibited divergent response patterns to *F. graminearum* infection between “Fielder” and “Yangmai 158”, and *TaSTP13-4A* which showed no induction in either cultivar (Table S11).

**Figure 8:**
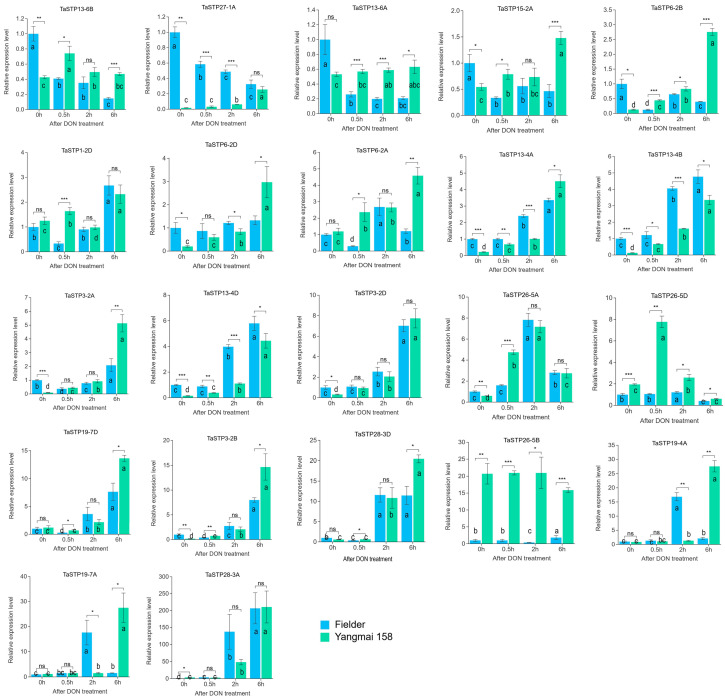
Comparison of the relative expression levels of 22 TaSTPs involved in different cultivars on DON treatments. The x-axes indicate the sample of DON treatments in the Fi and Y158; the y-axes indicate the relative gene expression levels. Capped lines indicate standard error. Intra-group differences are expressed by letters, and there are no differences between samples containing the same letters. Genes are grouped by expression amplitude. Note the different *Y*-axis scales. Differences between groups are indicated by *, ns *p* > 0.05; * *p* < 0.05; ** *p* < 0.01; *** *p* < 0.001.

Following 0.5 h DON treatment, 13 TaSTP genes were differentially expressed compared to the control in “Fielder”: *TaSTP1-2D*, *TaSTP3-2A*, *TaSTP3-2B*, *TaSTP6-2A*, *TaSTP6-2B*, *TaSTP13-6A*, *TaSTP13-6B*, *TaSTP15-2A*, *TaSTP19-7D*, *TaSTP27-1A*, and *TaSTP28-3D* were downregulated, while *TaSTP26-5A* was upregulated (Figure 8 and Table 3). In “Yangmai 158”, 15 TaSTP genes (*TaSTP1-2D*, *TaSTP3-2B*, *TaSTP3-2D*, *TaSTP6-2A*, *TaSTP6-2B*, *TaSTP6-2D*, *TaSTP13-4A*, *TaSTP13-4B*, *TaSTP13-4D*, *TaSTP13-6B*, *TaSTP15-2A*, *TaSTP19-7A*, *TaSTP26-5A*, *TaSTP26-5D*) were differentially expressed, all of which were upregulated.

At 2 h post-DON treatment, “Fielder” exhibited differential expression in 20 TaSTP genes. Among these, 13 TaSTP genes of them (*TaSTP3-2B*, *TaSTP3-2D*, *TaSTP6-2A, TaSTP13-4A*, *TaSTP13-4B*, *TaSTP13-4D*, *TaSTP19-4A*, *TaSTP19-7A*, *TaSTP19-7D*, *TaSTP26-5A*, *TaSTP26-5D*, *TaSTP28-3A*, and *TaSTP28-3D*) were upregulated, including *TaSTP28-3A,* which was upregulated by 142-fold. Seven genes (*TaSTP3-2A*, *TaSTP6-2B*, *TaSTP13-6A*, *TaSTP13-6B*, *TaSTP15-2A*, *TaSTP26-5*, and *TaSTP27-1*) were downregulated. “Yangmai 158” exhibited differential expression in 16 TaSTP genes, with fifteen genes (*TaSTP3-2A*, *TaSTP3-2B*, *TaSTP3-2D*, *TaSTP6-2A*, *TaSTP6-2B*, *TaSTP6-2D*, *TaSTP13-4B*, *TaSTP13-4D*, *TaSTP13-6A*, *TaSTP19-7D*, *TaSTP26-5A*, *TaSTP26-5D*, *TaSTP27-1A*, *TaSTP28-3A* and *TaSTP28-3D*) upregulated and *TaSTP1-2D* downregulated. 

At 6 h post-DON treatment, 14 TaSTP genes (*TaSTP1-2D*, *TaSTP3-2A*, *TaSTP3-2B*, *TaSTP3-2D*, *TaSTP6-2A*, *TaSTP13-4A*, *TaSTP13-4B*, *TaSTP13-4D*, *TaSTP19-4A*, *TaSTP19-7A*, *TaSTP19-7D*, *TaSTP26-5A*, *TaSTP28-3A*, and *TaSTP28-3D*) were upregulated, and six TaSTP genes (*TaSTP6-2B*, *TaSTP13-6A*, *TaSTP13-6B*, *TaSTP15-2A*, *TaSTP26-5D*, and *TaSTP27-1A*) were downregulated in “Fielder”. In “Yangmai 158”, 19 of the 22 TaSTP genes were upregulated (with *TaSTP26-5B* showing no significant change and *TaSTP26-5D* was downregulated); notably, *TaSTP3-2B* was upregulated by 95-fold.

A striking observation was that *TaSTP6-2B*, *TaSTP13-6A*, *TaSTP13-6B*, *TaSTP15-2A*, and *TaSTP27-1A* were consistently downregulated in “Fielder” but upregulated in “Yangmai 158” across all DON treatments. These findings indicate that TaSTP gene expression is fine-tuned by DON elicitation, with distinct molecular response mechanisms operating in resistant versus susceptible wheat cultivars.

## 3. Discussion

### 3.1. Expansion and Conservation of the TaSTP Gene Family in Wheat

The objectives of this study were to comprehensively identify the TaSTP gene family in wheat and to characterize their expression patterns to pinpoint candidates involved in FHB resistance. A recent study by Liu et al. [38] systematically identified 81 TaSTP genes in wheat and analyzed their expression under abiotic stress in seedlings. While our study confirmed the presence of the 81 TaSTP genes previously reported and identified one additional member, designated as *TaSTP6-2D*. Although *TaSTP6-2D* is located near *TaSTP6-2A* in the phylogenetic tree (Figure 1), a multiple sequence alignment confirmed that *TaSTP6-2A*, *TaSTP6-2B*, and *TaSTP6-2D* are distinct homoeologs with unique amino acid variations (Figure S21 and Table S12), supporting their designation as separate genes.

Our study, while confirming the vast majority of the findings by Liu et al. [38], extends this knowledge in several key aspects: (i) we report an additional gene, *TaSTP6-2D*; (ii) we utilize a more recent genome assembly (IWGSC RefSeq v2.1), which may enable more accurate gene modeling; and (iii) most significantly, we shift the focus to the response to Fusarium head blight and its key virulence factor, deoxynivalenol, across both susceptible and resistant cultivars. This provides crucial insights into the role of sugar transporters in an economically devastating disease, revealing potential candidate genes for future breeding efforts

The expansion of the STP family in wheat (82 members), compared to *A. thaliana* (14 members) [29] and *O. sativa* (28 members) [39], is consistent with patterns observed in other gene families following polyploidization events [40,41]. This genomic redundancy may provide the raw material for functional diversification and subfunctionalization among homoeologs [42]. The larger number of *STPs* in wheat can be attributed to its allohexaploid nature and highlights the substantial size and complexity of the wheat genome. However, the variation in the number of sugar transporter family members across wheat, *A. thaliana*, and *O. sativa* is not solely due to differences in genome size but may also reflect unidentified regulatory mechanisms or divergence in domain organization among these species.

Our analysis revealed an uneven distribution of TaSTP genes across subgenomes (30 in A, 27 in B, 25 in D) and chromosomes, with a notable density on homologous Chr 2 and 5 (Figure 4B and Table S6). This pattern suggests a potential evolutionary bias, with the A subgenome possibly retaining more functional responsibility for this family. The clustering of known regulators like *TaSTP3* and *TaSTP6* on Chr 2 [36,37] further highlights these genomic regions as hotspots for sugar transporter-mediated defense responses.

Comparative sequence analysis showed that all members of the sugar transporter family in wheat preserve three functionally important domains (MFS, XylE, and AraE) (Figure S2), suggesting these domains are necessary for maintaining their biochemical roles in sugar transport [43,44]. The STPs are classified as members of the MFS, which is capable of facilitating the transport of various monosaccharides, including glucose, fructose, galactose, and mannose [34,45,46]. Specific Arabidopsis STPs have been characterized, including defective transporter AtSTP5 [46], arabinose-specific AtSTP7 [46], glucose-specific AtSTP9 [47], and galactose-specific AtSTP14 [45].

### 3.2. Expression Patterns of TaSTP Genes Hint at Functional Diversification

The specific upregulation of *TaSTP9-6D* in anthers suggests a potential role in pollen development (Figure S11), a function analogous to *AtSTP9* and *AtSTP10* in Arabidopsis pollen [29,48]. However, functional similarity remains unconfirmed, as such correlations could arise from conserved regulatory mechanisms without guaranteeing equivalent biological roles.

In this study, we focused on TaSTPs involved in the response to *F. graminearum*, the causal agent of FHB. Previous studies demonstrated that wheat resistance to FHB is a quantitative trait controlled by multiple genes, with a complex regulatory mechanism influenced by factors such as the pathogen, wheat growth stage (most susceptible during heading to flowering), and environmental conditions (e.g., high temperature and humidity) [49,50]. The analysis of the expression patterns of STP family members during wheat development revealed dynamic transcriptional regulation (Figures S6–S14). For instance, *TaSTP3-2A* and *TaSTP3-2B* were highly expressed in the roots at the seedling stage (Figure S6) and the three-leaf stage (Figure S7), and in the first leaf blade at the tillering stage (Figure S8). During flowering, these genes exhibited elevated expression in the flag leaf, stigma, and ovary (Figure S11).

Hexose transport plays a pivotal role in diverse physiological processes, such as plant metabolic synthesis, sugar signaling, pollen development, and pollen tube elongation. Additionally, this transport mechanism responds to both external and internal factors and contributes to plant responses against biotic and abiotic stresses [15,16,17,29]. Previous studies also found that *TaSTP3* [36], *TaSTP6* [37], and *TaSTP13* [35] act as regulators of wheat stripe rust; the overexpression of these genes enhanced the susceptibility of wheat to stripe rust. However, no evidence currently links TaSTP genes to FHB regulation.

### 3.3. TaSTP Genes Are Potentially Pivotal in Wheat’s Response to FHB

The core of our study focused on leveraging expression profiling to identify TaSTPs involved in FHB response. The most striking response was observed for *TaSTP6-2D*, which exhibited a greater than 15-fold increase in expression upon DON treatment in the resistant variety “Yangmai 158” (Figure 8), suggesting it is a central player in the response. We found that a cohort of 11 genes, including *TaSTP1-2D*, *TaSTP3-2A*, *TaSTP3-2B*, *TaSTP6-2A*, *TaSTP6-2B*, *TaSTP13-4B*, *TaSTP13-4D*, *TaSTP19-4A*, *TaSTP26-5A*, *TaSTP28-3A*, and *TaSTP28-3D* were consistently upregulated by chitin, *F. graminearum* and DON treatment in both the “Fielder” and “Yangmai 158” samples (Figure 6A, Figure 7A and Figure 8; Table S13), indicating a core set of sugar transporters engaged in the general immune response.

However, a more nuanced pattern emerged when comparing the varieties. The resistant variety “Yangmai 158” often exhibited a stronger and more sustained upregulation of key TaSTPs. For instance, *TaSTP26-5A* showed a 28-fold upregulation in response to chitin in “Yangmai 158” compared to a 6-fold change in “Fielder” (Table 1 and Figure 6). The rapid, strong induction of *TaSTP26-5A* by chitin indicates a function in pattern-triggered immunity (PTI), a scenario analogous to Arabidopsis *AtSTP13*, which promotes resistance to *Botrytis cinerea* via accelerated glucose uptake upon pathogen perception [30]. The sustained upregulation of *TaSTP6-2D* in the resistant cultivar “Yangmai 158” following DON treatment is particularly intriguing. As DON is a mycotoxin primarily produced by *Fusarium graminearum* complex species, plays a critical role in the wheat-pathogen interaction [50,51]. Previous studies have shown that during *F. graminearum* infection, DON can activate *TaUGT6*, which converts it into the less phytotoxic DON-3-glucoside (D3G). This conjugation significantly reduces the capacity of D3G to inhibit protein synthesis and cause other damage, thereby influencing wheat resistance to FHB [52]”.

Concurrently, *TaSTP26-5A* expression peaked at 2 h after DON treatment in both varieties, followed by a subsequent decline (Figure 8). However, following pathogen inoculation, *TaSTP26-5A* showed only modest upregulation—merely 1.4-fold at 8 h in “Fielder” and 2.3-fold at 2 h in “Yangmai 158” (Table 2). This differential regulation suggests that the magnitude and timing of TaSTP activation may be a critical determinant of resistance outcomes, potentially by more efficiently competing for sugars or fueling defense responses.

### 3.4. Limitations and Future Perspectives

Previous studies have demonstrated that STPs modify plant resistance to pathogenic bacteria, primarily by engaging in the competition, distribution, and transport of sugars [18,19,20]. However, STP-mediated sugar partitioning poses significant regulatory challenges, and the precise function and mechanism of TaSTP*s* in response to FHB requires further experimental validation.

Despite these compelling correlations, several limitations and alternative interpretations of our data must be considered. Firstly, the correlative nature of our expression data cannot establish causality; functional validation through gene knockout or overexpression is essential to definitively prove the role of these TaSTPs in FHB resistance. Secondly, the observed downregulation of some genes (e.g., *TaSTP17-2A*) could be interpreted not as a failed defense but as a pathogen strategy to manipulate host sugar transport or a host tactic to compartmentalize resources away from the infection site. The precise function and mechanism of TaSTPs in this complex interaction remain a key question for future research.

In conclusion, building upon the work that identified 81 TaSTP genes [38], this study confirmed these and identified one additional novel gene, *TaSTP6-2D*. We provided the first comprehensive analysis of the expression patterns of TaSTP genes during wheat development and in response to FHB-related stresses. Our work establishes a solid foundation for elucidating the role of sugar transporters in wheat-*F. graminearum* interactions and paves the way for developing novel strategies to enhance FHB resistance through the genetic manipulation of sugar allocation.

## 4. Materials and Methods

### 4.1. Database Mining and Identification of TaSTP Genes in Wheat

The protein database Pfam (http://pfam-legacy.xfam.org/ (accessed on 1 January 2024)), the *Arabidopsis thaliana* database (https://www.arabidopsis.org/ (accessed on 6 January 2024)), and the rice genome database (http://plants.ensembl.org/Oryza_sativa/Info/Index (accessed on 8 January 2024)) were used to download and integrate the amino acid sequences of STPs. The STP sequences of *Arabidopsis* and rice were used as seed sequences to query the Chinese Spring database (IWGSCv2.1) via the online platform WheatOmics (http://wheatomics.sdau.edu.cn/ (accessed on 10 January 2024)). Since the IWGSCv1.1 annotation is currently recognized as the most comprehensive, subsequent analyses were primarily based on this version. Candidate wheat STP sequences were further validated by BLAST (accessed on 1 February 2024) searches and comparison with existing studies to obtain the complete set of wheat STP sequences [38].

### 4.2. Phylogenetic Analysis, Motif Prediction, and Conserved Domain Analysis

The protein sequences of 14 *Arabidopsis thaliana*, 28 *Oryza sativa*, and 82 *Triticum aestivum* L. STPs were utilized to perform a multi-sequence alignment using MEGA-X [53]. The alignment files were obtained using the default parameters of MUSCLE, and a phylogenetic tree was constructed using a 1000 bootstrap value with the neighbor-joining algorithm [54]. Finally, the data were visualized by TBtools (version 2.345). In this study, MEME Suite 5.5.7 (https://meme-suite.org/meme/tools/meme (accessed on 10 March 2024)) was used to predict STP motif in wheat, and Batch CD-Search (https://www.ncbi.nlm.nih.gov/Structure/bwrpsb/bwrpsb.cgi (accessed on 10 March 2024)) in NCBI was used to predict STPs Domain in wheat. Additionally, the subcellular localization prediction of the 82 TaSTP genes was predicted using WoLF PSORT (https://wolfpsort.hgc.jp (accessed on 1 May 2024)). The protein domain analysis of each protein sequence was conducted using InterPro (http://www.ebi.ac.uk/interpro/ (accessed on 1 May 2024)) and subsequently visualized using Tbtools (version 2.345).

### 4.3. Naming of TaSTP Genes

To resolve inconsistencies in previous naming systems, a uniform nomenclature was established based on phylogenetic relationships and chromosomal locations within the A, B, and D subgenomes. Each gene name begins with “Ta” for *Triticum aestivum* L., followed by “STP” and a number indicating phylogenetic clade affiliation (e.g., genes clustering with *OsSTP1* (*TraesCS2A02G340600*, *TraesCS2B02G338400*, and *TraesCS2D02G318200*) were named *TaSTP1*). Wheat genes contain A, B, and D genomes, and *TraesCS2A02G340600*, *TraesCS2B02G338400*, and *TraesCS2D02G318200* were designated *TaSTP1-2A*, *TaSTP1-2B*, and *TaSTP1-2D* (wherein the 2A in *TaSTP1-2A* signifies the gene on chromosome 2 of the A genome). It should be noted that multiple TaSTP genes are present in the same branch as Arabidopsis or rice. For instance, there are 12 TaSTP genes in the same branch as *AtSTP7*, and there are multiple TaSTP genes in the same genome. To distinguish between these, we will add “number order” to distinguish in this case. For example, the same branch as *AtSTP7* contains four TaSTP genes (in addition to the named *TaSTP26-5D*), all located on Chr 5 of the D genome. According to the established nomenclature, *TraesCS5D02G234100*, *TraesCS5D02G234200*, *TraesCS5D02G558100*, and *TraesCS5D02G549900* are named successively as *TaSTP7-5D.1*, *TaSTP7-5D.2*, *TaSTP7-5D.3* and *TaSTP7-5D.4*. The full naming details for all 82 TaSTP genes are provided in Table S2.

### 4.4. Plant Materials, Growth Conditions, and Stress Treatments

The study was conducted over two growing seasons (2023–2024 and 2024–2025). “Fielder” (susceptible) and “Yangmai 158” (resistant) wheat varieties were planted in the experimental field of Yangzhou University in Yangzhou City, Jiangsu Province, China (119°26′ E, 32°24′ N) under typical fertilizer and water management. The field experiments were conducted at the experimental station. Wheat plants that flowered on the same day were marked and selected for inoculation with *F*. *graminearum*.

For the *F. graminearum* inoculation, plants were inoculated at the flowering stage by injecting *PH-1* conidial suspension (10^5^ spores/mL) into a single floret in the central section of the spike. For the chitin and DON treatment, two-week-old seedlings were used with a solution of 25 μg/mL chitin and 50 μL/mL DON. The numbers of symptomatic and total spikelets of “Fielder” and “Yangmai 158” were calculated at 21 days after inoculation with *F. graminearum*, respectively. The disease severity is described in terms of the percentage of symptomatic spikelets (PSS). The samples of wheat spikelets with uninoculated (0), 2 h (2 h), 8 h (2 h), 24 h (24 h), and 48 h (48 h) after inoculation were collected and immediately placed in liquid nitrogen and stored at −80 °C. Three replicates of each sample were collected.

### 4.5. F. graminearum, Chitin, and DON Configuration

Activating *F. graminearum* (strain PH-1) from storage onto Potato Dextrose Agar (PDA) involves reviving the fungus to restore its viability and promote growth. Mycelium plugs were transferred to mung bean broth and incubated at 25 °C with shaking (180 rpm) for 2–3 days. Finally, the spore concentration was diluted to 10^5^ (spores/mL). Chitin (Sigma-Aldrich (St. Louis, MO, USA), C9752, BioReagent, suitable for analysis) was dissolved in sterilized double distilled water (250 ug/mL) and then lysed by ultrasonic waves until only a small amount of precipitate was obtained. Prior to use, the chitin solution was diluted to a working concentration of 25 μg/mL. The DON solution was prepared by adding 1 mg of DON powder (Sigma-Aldrich, D0156) to 20 mL of double-distilled water, yielding a working concentration of 50 μg/mL.

### 4.6. RNA Extraction, Reverse Transcriptase, and RT-qPCR

Total RNA was extracted from the samples using a TransZol Up kit (TransGen Biotech, Beijing, China). This kit is designed to extract total RNA from a variety of samples. The extracted RNA was then treated with DNase I to remove any DNA contamination. The concentration of RNA was then detected using a Nanodrop instrument (Thermo-Scientific™ Model), and the first strand of cDNA was synthesized using 5 μg RNA and M-MLV reverse transcriptase (Thermo-scientific (Waltham, MA, USA)). The sequences of TaSTPs were downloaded from the database (http://wheatomics.sdau.edu.cn/ (accessed on 1 March 2024)). The coding sequence (CDS) of TaSTPs were designed using BeaconDesign 7.9 to design selected gene-specific primers for TaSTP genes (Table S14). RT-qPCR was performed on a Bio-Rad CFx384-well instrument (Bio-Rad (Hercules, CA, USA)) using AceQ Universal SYBR qPCR Master Mix (Vazyme, Nanjing, China) according to the instructions. The reaction system comprised 12 µL, including 6 µL of 2× SYBR qPCR Supermix, 0.8 µL of nuclease-free water, 0.2 µL of each primer (10 mM), and 5 µL of cDNA (~100 µg/µL). The program was as follows: pre-denaturation at 95 °C (5 min) and 40 cycles at 95 °C (10 s) and 60 °C (30 s).

### 4.7. Data Analysis

The database (http://wheatomics.sdau.edu.cn/expression/index.html (accessed on 1 June 2024)) was utilized to query the RNA-seq data of TaSTP genes in wheat tissues (roots, stems, leaves, spikelets, and grains) and under biotic and abiotic stresses, and TBtools (version 2.345) generated the heat map. For RT-qPCR, *TaACTIN* was used as an internal reference gene. Relative expression levels were calculated using the 2^−∆∆Ct^ method [55], with untreated “Fielder” samples (Fi-0) as the control. Statistical significance was determined by Student’s *t*-test (*p* ≤ 0.05). Data analysis and K-means [56] were performed using the Metware Cloud, a free online platform for data analysis (https://cloud.metware.cn (accessed on 1 March 2025)). Heatmaps were generated with TBtools (version 2.345).

## 5. Conclusions

In this study, we confirmed the 81 TaSTP genes previously identified by Liu et al. [38] and identified one additional novel gene, *TaSTP6-2D*. In addition, an investigation of the expression patterns of TaSTP genes in response to chitin, F. graminearum, and DON treatments was conducted using RT-qPCR. The results demonstrated that *TaSTP1-2D*, *TaSTP3-2A*, *TaSTP3-2B*, *TaSTP6-2A*, *TaSTP6-2B*, *TaSTP13-4B*, *TaSTP13-4D*, *TaSTP19-4A*, *TaSTP26-5A*, *TaSTP28-3A*, and *TaSTP28-3D* may play significant roles in wheat FHB resistance.

However, it is important to acknowledge that the functional implications of these findings are largely correlative, based primarily on expression evidence. The precise mechanistic role of these candidate genes in FHB resistance requires further functional validation. Despite these limitations, this study provides a crucial foundation for future work. The highest priorities include the functional characterization of candidates through genetic approaches (e.g., gene knockout or overexpression) to establish causality, and the biochemical determination of their substrate specificity. Furthermore, elucidating the upstream regulatory networks that control their induction during pathogen infection will be essential for a complete understanding of their role in disease resistance.

In summary, this work systematically explores the potential of the TaSTP gene family in regulating FHB resistance. It not only lays the groundwork for a deeper understanding of their biological functions but also defines clear and specific targets for future genetic improvement of wheat against FHB.

## Figures and Tables

**Table 1:** The Fold Change (FC) list of TaSTP genes after chitin treatment.

Gene Name	Fielder			Yangmai158			Selected Genes
	0 vs. 5 M	0 vs. 15 M	0 vs. 30 M	0 vs. 5 M	0 vs. 15 M	0 vs. 30 M	
TaSTP1-2A	0.80	1.72 *	1.77 *	1.26	1.28	1.45 *	√
TaSTP1-2D	1.42 **	0.97	0.67 **	1.86 *	2.63 **	1.96 **	√
TaSTP3-2A	0.85	1.79 **	3.76 *	1.55 **	1.80 *	3.18 **	√
TaSTP3-2B	1.11	1.96 *	2.78 **	1.29 *	2.27 **	3.33 **	√
TaSTP3-2D	2.39	2.37 *	6.11 **	2.10 *	2.54 **	4.20 **	√
TaSTP3-3A.1	0.90	1.90 *	2.21 *	0.71 *	1.22 *	2.27 *	√
TaSTP3-3B.1	0.95	0.94	0.72 **	1.32 **	1.55 **	1.32 *	√
TaSTP6-2A	1.54	2.54 **	1.23	1.03	1.88 **	3.39 **	√
TaSTP6-2B	0.56 *	0.37 **	0.32 **	3.08 **	5.38 **	5.71 **	√
TaSTP6-2D	1.66 *	7.71 *	5.14 **	1.15	1.78 **	0.70	√
TaSTP7-5A.1	1.13	0.97	0.96	1.22	1.21	1.42 **	
TaSTP7-5A.2	1.58 **	1.16 *	0.69 *	1.27	1.32	1.23	
TaSTP7-5B.1	1.38 *	1.39 **	1.35 *	1.31 **	1.74 **	2.38 **	√
TaSTP7-5B.2	1.46 **	1.14 *	1.05	1.50 **	1.44 **	2.10 **	√
TaSTP7-5D.1	1.43 *	1.07	0.58 *	1.53 **	1.25 *	2.37 **	√
TaSTP10-1A.1	0.64	1.73	1.36	1.24	1.16	0.90	
TaSTP11-6A	0.96	1.34	1.21	1.05	0.58 *	0.41 **	√
TaSTP13-4A	2.32 **	6.07 **	4.31 **	2.02 *	3.47 **	4.37 *	√
TaSTP13-4B	2.29 **	4.34 **	5.00 **	3.34 **	7.35 **	12.76 **	√
TaSTP13-4B.1	0.56 *	0.39 **	0.43 *	1.35	0.84	0.67	
TaSTP13-4D	1.46 **	2.61 **	3.22 **	2.19 **	3.96 **	5.61 **	√
TaSTP13-4D.1	0.75	0.68	0.30 **	0.95	0.60 *	0.65 *	
TaSTP13-6A	0.75 *	0.96 *	0.69 *	1.55 **	2.28 **	1.01	√
TaSTP13-6B	0.89	1.12	1.15	1.32 **	1.51 **	1.08	√
TaSTP14-5A	0.74 *	0.77 **	0.81 *	1.05	0.94	0.73 **	
TaSTP14-4B	0.85	0.93	0.58 *	0.62	0.48	0.58	
TaSTP14-4D	1.17	1.02	0.34 **	1.07	1.12	0.90	
TaSTP15-2A	2.07 *	1.83 *	2.00 *	3.59 **	6.49 **	3.71 **	√
TaSTP15-2B	0.58 **	0.86 *	0.94	0.63 **	0.76 *	0.98	
TaSTP16-2B	1.91 **	4.95 **	2.95 *	1.62	2.75 **	5.10 **	√
TaSTP17-2A	1.59	0.93	0.66	1.68 *	1.12	1.34 *	
TaSTP17-2B.1	0.69 *	0.69 *	0.34 **	1.09	0.86	0.62 *	
TaSTP17-2D	1.19	0.91	0.36 **	1.23	1.00	1.50 *	
TaSTP18-2A	1.11	0.86	0.75	0.86	1.35	0.92	√
TaSTP18-2B	1.02	0.84 *	0.87	0.88	1.00	1.25	
TaSTP18-2D	1.04	1.06	1.12	0.77 *	0.95	0.85	
TaSTP19-4A	3.82 **	3.47 **	2.66 **	1.51 *	0.52 *	0.09 *	√
TaSTP19-7A	0.58*	0.67 *	0.85	1.19	0.60 *	0.39 **	√
TaSTP19-7D	0.59 **	1.29	1.71 *	0.60	0.53 *	0.47 *	√
TaSTP25-5A	0.58 *	0.71	1.07	1.32	1.39	1.13	√
TaSTP25-5B	0.89	1.01	0.89	1.16	1.21 *	1.03	√
TaSTP25-5D	0.89 *	1.03	0.91 *	1.30 **	1.86 **	1.57 **	√
TaSTP26-5A	2.01 **	5.83**	6.03 **	3.01 **	13.69 **	28.11 **	√
TaSTP26-5B	0.75	0.55**	0.39 **	0.86	0.95	0.72 **	√
TaSTP26-5D	1.48	3.40**	2.38 **	2.93 **	1.54 **	0.71 **	√
TaSTP27-1A	0.88	0.75	0.72	3.09 *	5.39 *	3.93 **	√
TaSTP28-1B.1	0.87 *	0.91	0.67	2.05 **	1.84 *	1.43 **	
TaSTP28-3A	2.53 *	8.45*	21.33 **	0.77	1.78 *	3.71 *	√
TaSTP28-3B	1.30 *	1.27*	1.06	1.27 **	1.60 **	3.44 **	√
TaSTP28-3D	0.97	2.07**	2.93 **	1.88 **	1.99 *	2.00	√

Note: “vs.” abbreviation for “versus” indicating the groups being compared; FC > 1: The gene is upregulated; FC < 1: The gene is downregulated; *\**: Typically used to mark statistically significant differences (* *p* < 0.05, ** *p* < 0.01); √: The gene will be selected to confirm induction levels under *F. graminearum* infection using RT-qPCR.

**Table 2:** The Fold Change (FC) list of TaSTP genes after *F. graminearum* infection.

Gene Name	Fielder				Yangmai158				Selected Genes
	0 vs. 2 h	0 vs. 8 h	0 vs. 24 h	0 vs. 48 h	0 vs. 2 h	0 vs. 8 h	0 vs. 24 h	0 vs. 48 h	
TaSTP1-2A	1.82 **	4.28 **	2.93 **	2.84 **	2.19 *	2.84 **	2.04 *	2.15 **	
TaSTP1-2D	0.91	0.91	1.03	1.61 *	1.41 *	1.22 *	1.09	1.33 *	√
TaSTP3-2A	2.45 *	4.69 *	1.93 *	7.59 *	23.10	24.85 *	7.37 **	8.02 *	√
TaSTP3-2B	8.91 **	6.09 **	2.59 **	6.69 **	10.01 **	8.85 **	4.91 **	5.72 **	√
TaSTP3-2D	5.25 **	4.22 **	1.70 **	4.57 **	10.43 **	7.58 **	4.03 **	6.34 **	√
TaSTP3-3A.1	6.10 **	4.82 *	1.63	7.20 **	14.20 **	9.49 *	4.13 *	5.90 **	
TaSTP3-3B.1	7.20 **	7.40 **	2.45 **	7.96 **	14.40 **	11.06 **	4.75 **	4.90 **	
TaSTP6-2A	0.94	0.93	1.30 **	2.70 **	6.05 **	1.70 **	1.14	2.82 **	√
TaSTP6-2B	1.04	0.62 **	1.34 **	1.31	21.37 **	2.88 **	1.97 **	5.56 **	√
TaSTP6-2D	3.85 **	3.13 **	1.39	5.75 **	20.91 **	4.45 **	3.75 **	10.61 *	√
TaSTP7-5B.1	1.35 *	2.02 **	1.30 *	0.50 **	1.27 *	1.43	0.65	0.85	
TaSTP7-5D.1	0.25 *	0.21 *	0.32 *	0.03 *	2.46 **	0.43 **	0.63 *	0.91 **	
TaSTP7-5B.2	0.39 *	0.41 *	0.41 *	0.34 **	1.41 **	0.66 **	1.02	0.87 *	
TaSTP11-6A	1.83 **	5.96 *	4.67 **	2.56	1.27	4.42 **	3.14 *	1.66 **	
TaSTP13-4A	1.04	1.07	0.78	0.66	1.10	0.60	0.56	0.72	√
TaSTP13-4B	4.96 **	3.19 **	1.20 *	5.92 **	10.47 **	4.47**	4.14 **	7.21 **	√
TaSTP13-4D	3.48 *	2.42 **	1.21 *	4.84 **	5.93 **	4.48 **	3.87 **	7.12 **	√
TaSTP13-6A	1.06	1.08	1.14	1.97 *	5.95 **	3.19 **	2.33 *	5.73 **	√
TaSTP13-6B	1.04	1.85 *	1.82 *	2.22 **	4.66 *	2.86 **	3.55 **	2.67 **	√
TaSTP15-2A	1.78 **	2.06 **	1.72 **	1.36 **	1.23 *	0.64 **	0.60 **	1.32 *	√
TaSTP16-2B	0.68 **	0.58 **	0.83 **	0.65 *	0.76 *	0.81 *	0.92	0.81 *	
TaSTP18-2A	1.14	0.16 **	0.22 **	0.22 **	0.21 **	0.21 **	0.12 **	0.42 **	
TaSTP19-4A	1.62 *	0.89	1.86 **	2.12 **	3.16 *	7.42 **	8.19 **	1.13 **	√
TaSTP19-7D	1.80 **	2.18 *	1.58 *	2.03 *	8.19 **	4.33 **	5.80 **	2.44 **	√
TaSTP19-7A	1.37 *	0.99	1.70 *	2.18 *	2.32 **	2.27 **	3.59 **	0.32 **	√
TaSTP25-5A	2.55 **	4.37 **	3.44 **	4.83 *	3.16 **	3.86 **	2.05 **	2.72 **	
TaSTP25-5B	1.29 *	2.11 **	1.88 **	2.47 **	2.68 *	2.21 **	1.09	2.42 *	
TaSTP25-5D	1.37 **	2.22 **	1.96 **	2.30**	3.35 **	2.66 **	1.47 **	2.34 **	
TaSTP26-5A	1.30	1.35 *	0.85	0.88	2.30 **	1.45 **	0.96	1.16	√
TaSTP26-5B	0.66 **	0.71 *	0.52 **	0.51 **	1.61 *	1.33	1.07	0.84	√
TaSTP26-5D	0.49 **	1.23 **	0.94 *	1.03	2.39 **	2.02 **	1.57 **	1.36 **	√
TaSTP27-1A	1.54 *	2.31 **	3.54 **	3.54 **	7.34 **	4.03 **	3.32 *	4.44 **	√
TaSTP28-3A	4.52 **	8.81 **	3.28 **	4.18 **	7.45 *	10.12 **	2.62 *	6.46 **	√
TaSTP28-3B	1.47*	3.61 **	2.23 *	5.06 **	7.43 **	4.04 *	3.03 *	3.70 **	
TaSTP28-3D	2.15 *	5.58 **	1.92 **	4.61 **	3.05 *	8.13 **	4.41 **	3.97 **	√

Note: “vs.” abbreviation for “versus,” indicating the groups being compared; FC > 1: The gene is upregulated; FC < 1: The gene is downregulated; *\**: Typically used to mark statistically significant differences (* *p* < 0.05, ** *p* < 0.01); √: The gene will be selected to confirm induction levels with DON treatment using RT-qPCR.

**Table 3:** The Fold Change (FC) list of TaSTP genes under DON treatment.

Gene Name	Fielder			Yangmai 15		
	0 vs. 0.5 h	0 vs. 2 h	0 vs. 6 h	0 vs. 0.5 h	0 vs. 2 h	0 vs. 6 h
TaSTP1-2D	0.34 **	0.91	2.71 **	1.31 *	0.79 *	1.85 *
TaSTP3-2A	0.38 **	0.78 **	2.10 *	4.66 **	9.71 **	53.65 **
TaSTP3-2B	0.41 **	2.76 *	8.17 **	4.76 **	12.85 **	94.78 **
TaSTP3-2D	1.09	2.65 **	7.21 **	2.99 **	6.59 *	24.64 **
TaSTP6-2A	0.30 **	2.68 *	1.21 *	2.02 *	2.28 **	3.93 **
TaSTP6-2B	0.13 **	0.66 *	0.40 *	3.40 **	6.36 **	21.23 **
TaSTP6-2D	0.85	1.25	1.38	2.93 *	4.20 **	14.85 **
TaSTP13-4A	1.00	2.41 **	3.37 **	3.09 **	4.62	20.61 **
TaSTP13-4B	1.23	4.07 **	4.80 **	5.15 **	12.43 **	26.07 **
TaSTP13-4D	0.88	3.98 **	5.82 **	2.53 **	7.36 **	29.16 **
TaSTP13-6A	0.27 *	0.20 **	0.22 **	1.07	1.11 *	1.19
TaSTP13-6B	0.41 **	0.36 **	0.15 **	1.74 *	1.16	1.10 *
TaSTP15-2A	0.34 *	0.56 **	0.49 *	1.47 *	1.26	2.76 **
TaSTP19-4A	1.39	17.39 **	2.13 *	1.37	1.67	36.28 **
TaSTP19-7A	1.59	18.13 *	1.57 **	1.28 *	1.27	23.24 **
TaSTP19-7D	0.28 *	3.99 *	7.75 **	0.6	2.01 *	12.23 **
TaSTP26-5A	1.60 **	7.82 **	2.82 **	7.95 **	12.01 **	4.59 **
TaSTP26-5B	1.12	0.40 *	1.88	1.03	1.01	0.77
TaSTP26-5D	1.06	1.23 *	0.41 **	4.00 **	1.33 *	0.31 **
TaSTP27-1A	0.59 **	0.49 **	0.32 **	1.85	3.69 **	15.27 **
TaSTP28-3A	5.68	141.87 *	206.36 **	0.85	14.34 **	59.54 **
TaSTP28-3D	0.38 *	11.95 **	11.67 **	1.1	18.55 **	34.79 **

Note: “vs.” abbreviation for “versus,” indicating the groups being compared; FC > 1: The gene is upregulated; FC < 1: The gene is downregulated; *\**: Typically used to mark statistically significant differences (* *p* < 0.05, ** *p* < 0.01).

## Data Availability

The original contributions presented in this study are included in the article/Supplementary Materials. Further inquiries can be directed to the corresponding author(s).

## Supplementary Materials

The following supporting information can be downloaded at: https://www.mdpi.com/article/10.3390/plants14192976/s1, Figure S1: Phylogenetic tree of the putative TaSTPs; Figure S2: Conserved domain analysis of TaSTP genes family members; Figure S3: Prediction of transmembrane helices in the TaSTP genes family; Figure S4: Chromosomal distribution of the sugar transporter gene family in wheat; Figure S5: Expression profiles of TaSTP genes in wheat tissues; Figure S6: Expression profiles of TaSTP genes in wheat during the seedling stage; Figure S7: Expression profiles of TaSTP genes in wheat during the three leaf stage; Figure S8: Expression profiles of TaSTP genes in wheat during the tillering stage; Figure S9: Expression profiles of TaSTP genes in wheat during the flag leaf stage; Figure S10: Expression profiles of TaSTP genes in wheat during the 30% spike stage; Figure S11: Expression profiles of TaSTP genes in wheat during the anthesis stage; Figure S12: Expression profiles of TaSTP genes in wheat during the milk grain stage; Figure S13: Expression profiles of TaSTP genes in wheat during ripening; Figure S14: Expression profiles of TaSTP genes under abiotic stress; Figure S15: Dynamic expression patterns of five key candidate TaSTP genes in different tissues throughout the entire growth period of wheat; Figure S16: Expression profiles of TaSTP genes under biotic stress; Figure S17: Expression profiles of TaSTP genes were infected by F. graminearum and treated with DON; Figure S18: Expression profiles of TaSTP genes of wheat leaf under phytohormone treatment; Figure S19: A conceptual model illustrating the potential role of TaSTP genes in integrating stress signals for FHB resistance; Figure S20: Venn diagram of TaSTP genes under chitin treatment; Figure S21: MUSCLE multiple sequence alignment of TaSTP6-2A and TaSTP6-2A; Table S1: The results of conserved domain detection using the NCBI Batch CD-Search Tool; Table S2: Information about the TaSTP genes in wheat; Table S3: Named of TaSTP genes in wheat; Table S4: The results of subcellular localization prediction of TaSTP genes; Table S5: The results of transmembrane helices prediction of TaSTP genes; Table S6: Chromosomal distribution and physical positions of TaSTP genes in wheat; Table S7. The PSSs (%) for the Fielder and Yangmai 158 tested in 2024 and 2025 field experiments; Table S8: List of TaSTP genes by chitin treatment using RT-qPCR; Table S9: The relative expression levels analysis of TaSTP genes under chitin treatment; Table S10: The relative expression levels analysis of TaSTP genes under *F. graminearum* infection; Table S11: The relative expression levels analysis of TaSTP genes under DON treatment; Table S12: The amino acid sequences of the TaSTP6-2A and TaSTP6-2D; Table S13: Summary table of TaSTP genes validation results using RT-qPCR; Table S14: The list of primer for RT-qPCR.
