# Supplementary material for: Genome-Wide Identification and Expression Profiling of Sugar Transport Protein Response to Fusarium Head Blight in Wheat (Triticum aestivum L.)
Source: Plants (Basel). 2025 Sep 25;14(19):2976. doi: 10.3390/plants14192976 (PMC12526077; doi:10.3390/plants14192976)
Supplement: Supplementary file 1 [file plants-14-02976-s001.zip › Figure S1-S18.pptx]

## Slide 1
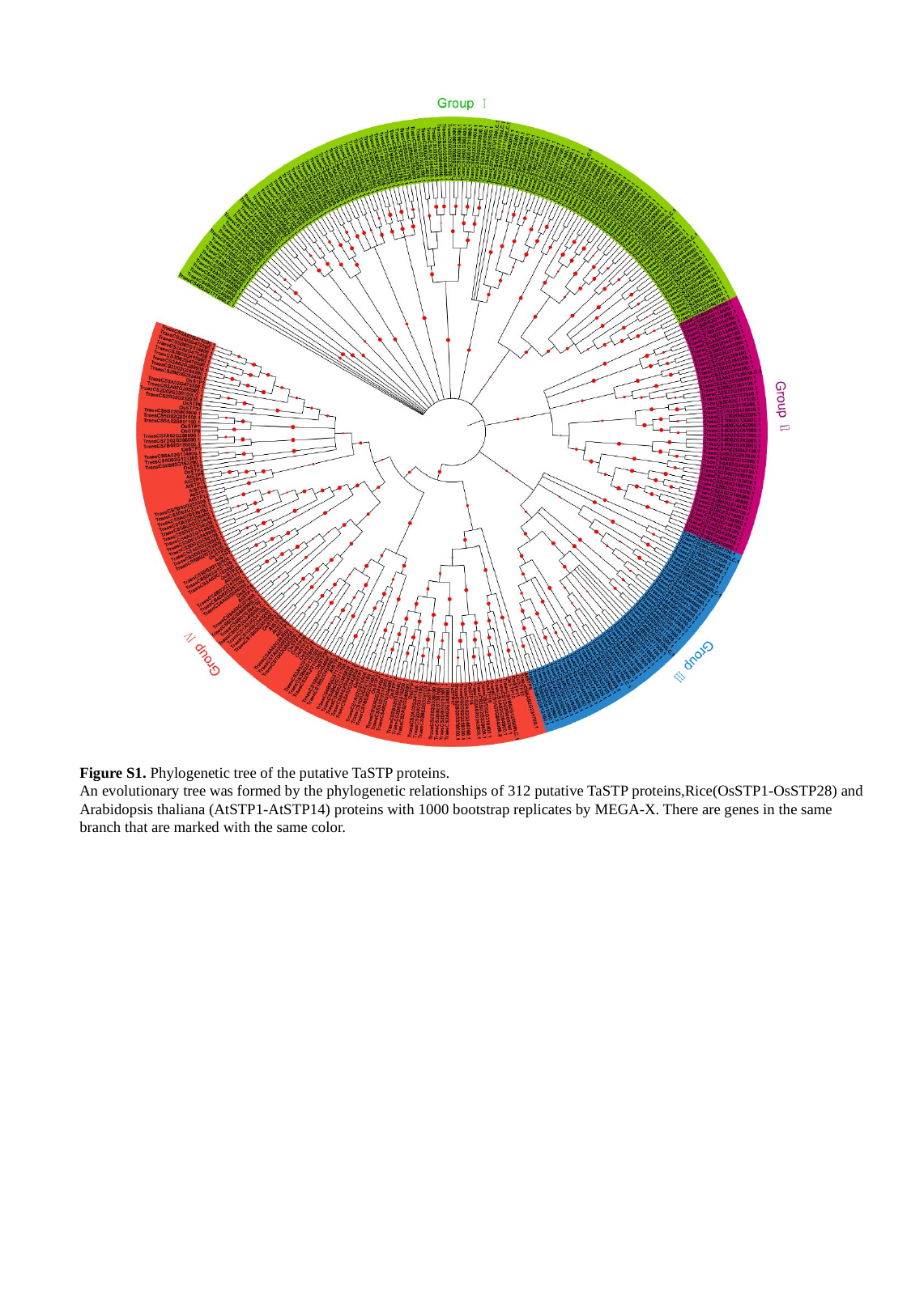

Figure S1. Phylogenetic tree of the putative TaSTP proteins.
An evolutionary tree was formed by the phylogenetic relationships of 312 putative TaSTP proteins,Rice(OsSTP1-OsSTP28) and Arabidopsis thaliana (AtSTP1-AtSTP14) proteins with 1000 bootstrap replicates by MEGA-X. There are genes in the same branch that are marked with the same color.

## Slide 2
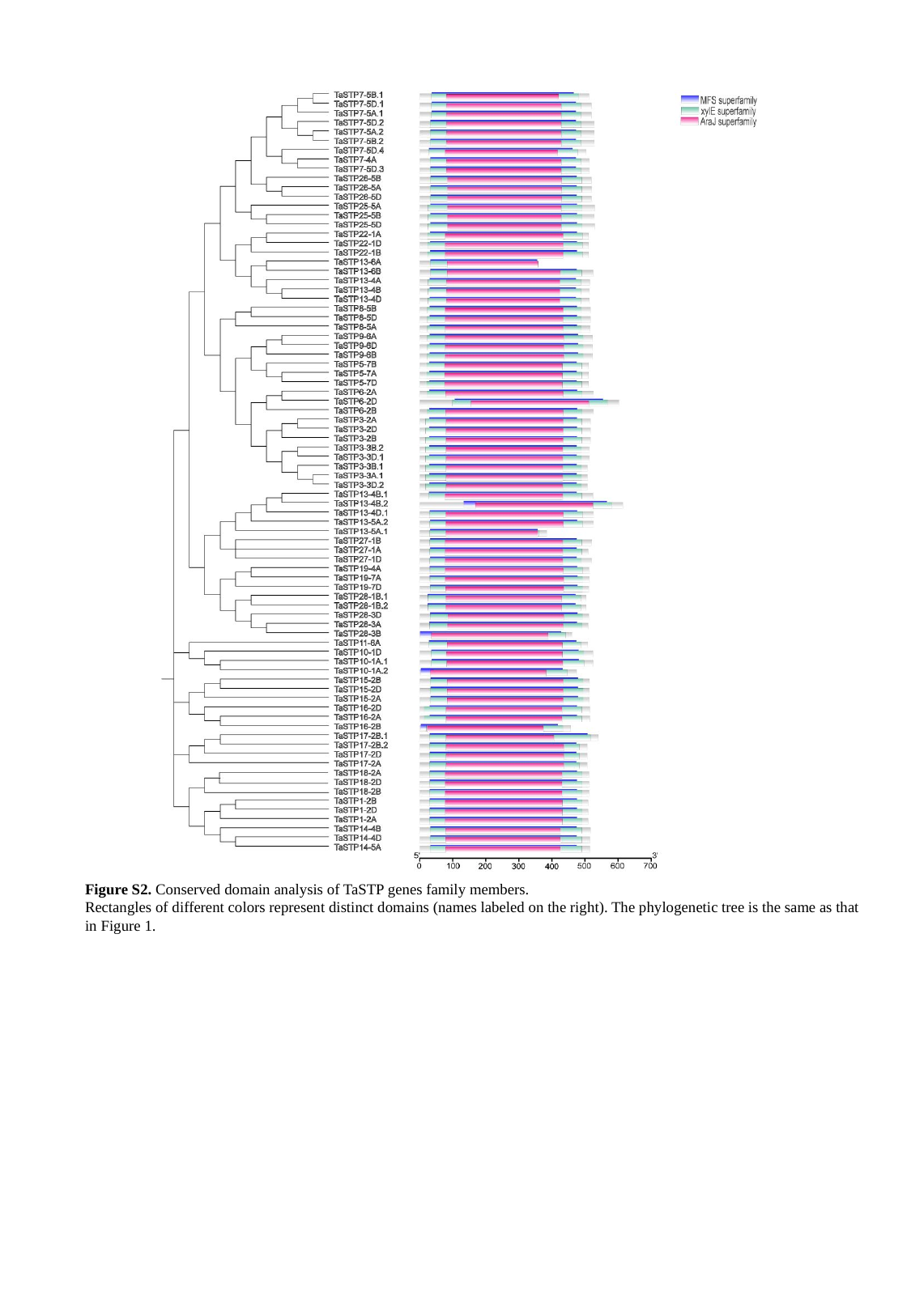

Figure S2. Conserved domain analysis of TaSTP genes family members.
Rectangles of different colors represent distinct domains (names labeled on the right). The phylogenetic tree is the same as that in Figure 1.

## Slide 3
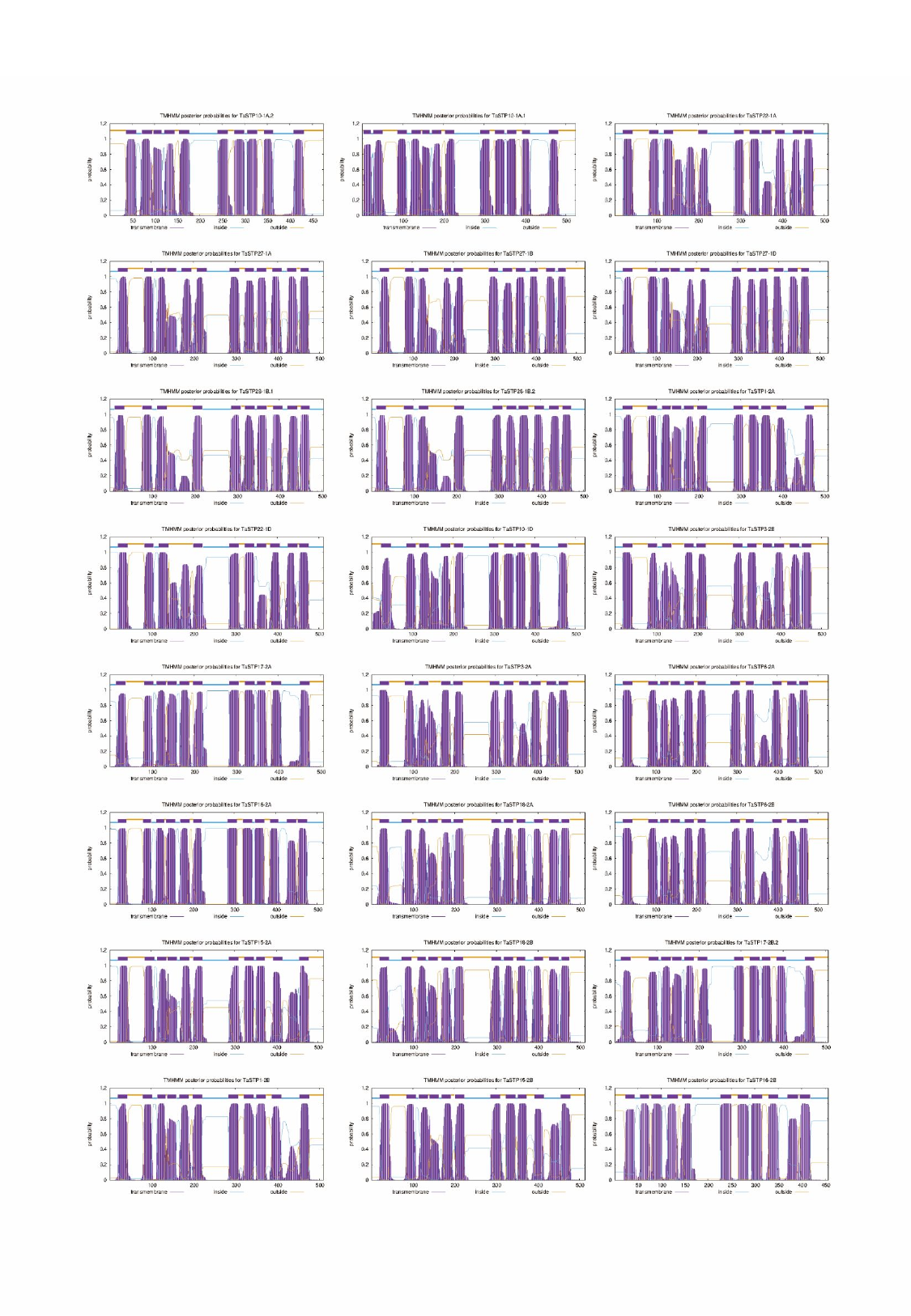

## Slide 4
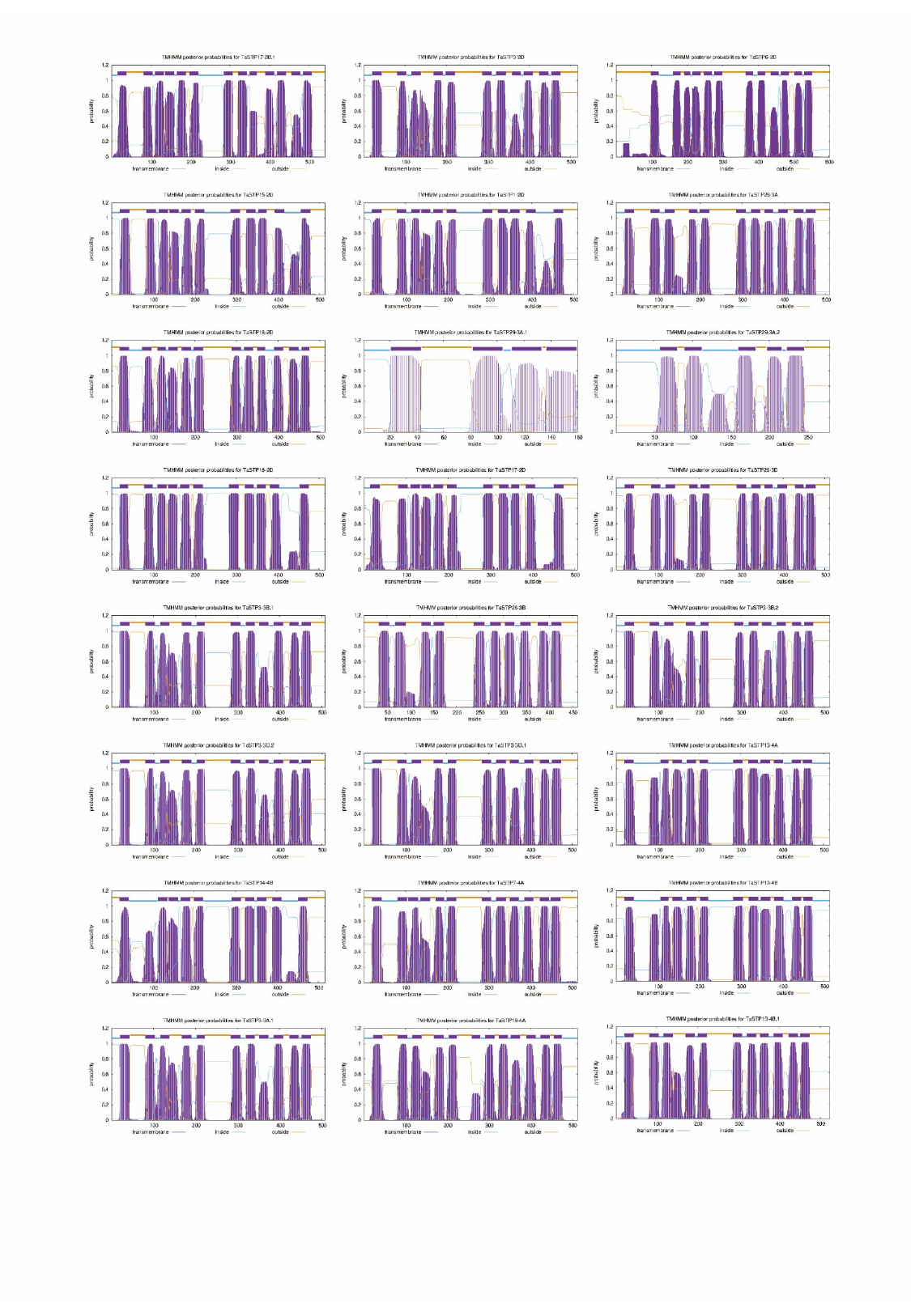

## Slide 5
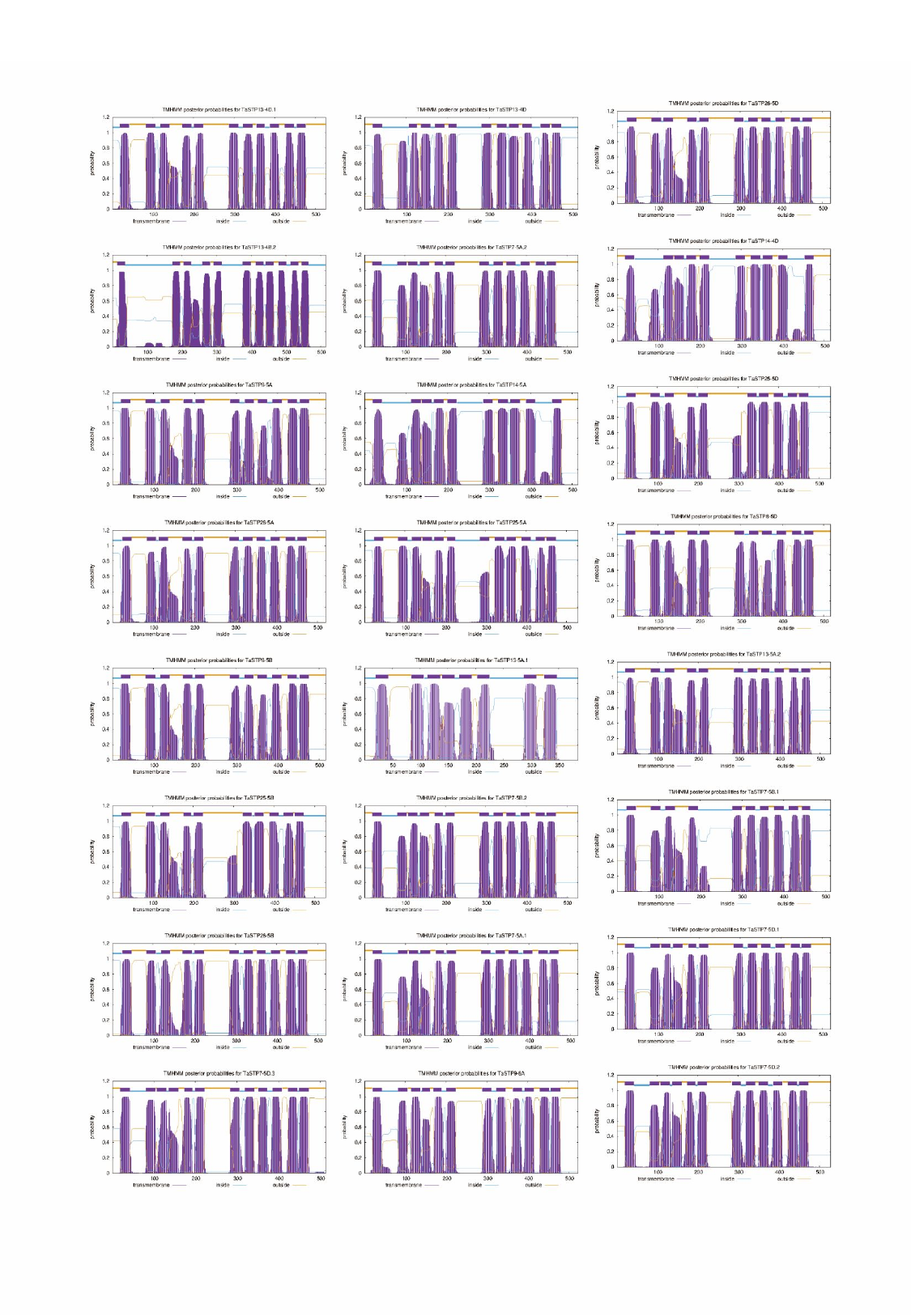

## Slide 6
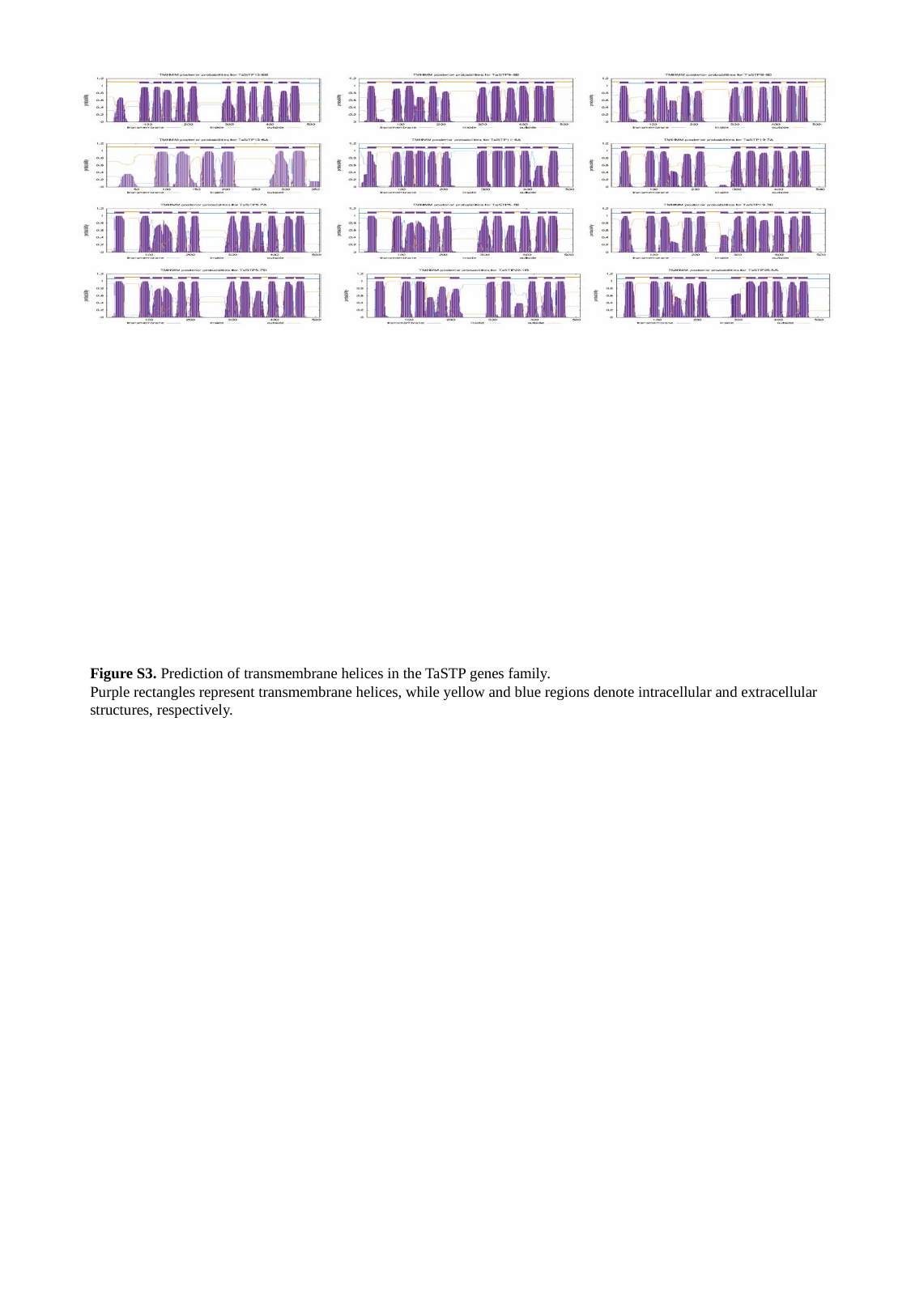

Figure S3. Prediction of transmembrane helices in the TaSTP genes family.
Purple rectangles represent transmembrane helices, while yellow and blue regions denote intracellular and extracellular structures, respectively.

## Slide 7
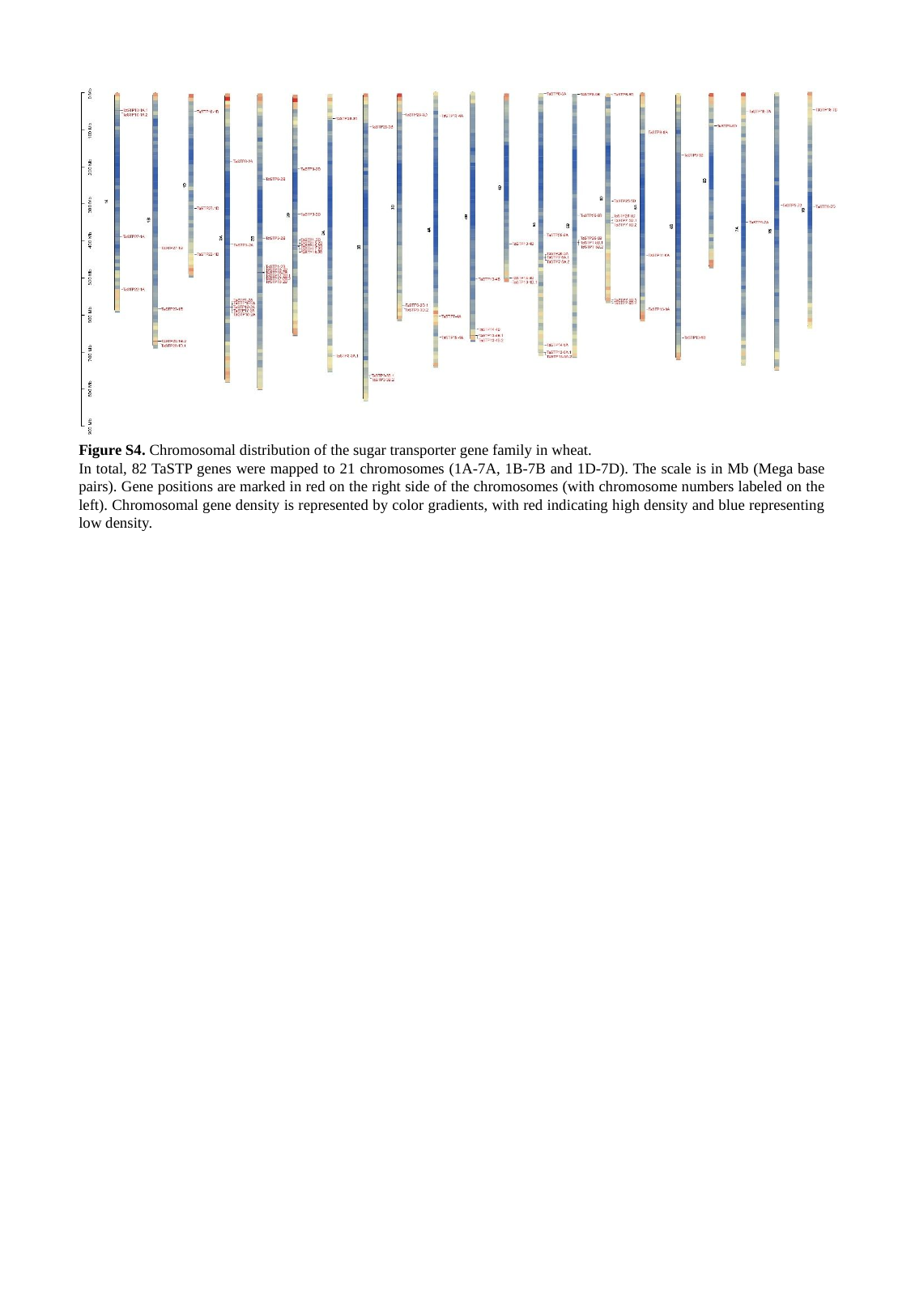

Figure S4. Chromosomal distribution of the sugar transporter gene family in wheat.
In total, 82 TaSTP genes were mapped to 21 chromosomes (1A-7A, 1B-7B and 1D-7D). The scale is in Mb (Mega base pairs). Gene positions are marked in red on the right side of the chromosomes (with chromosome numbers labeled on the left). Chromosomal gene density is represented by color gradients, with red indicating high density and blue representing low density.

## Slide 8
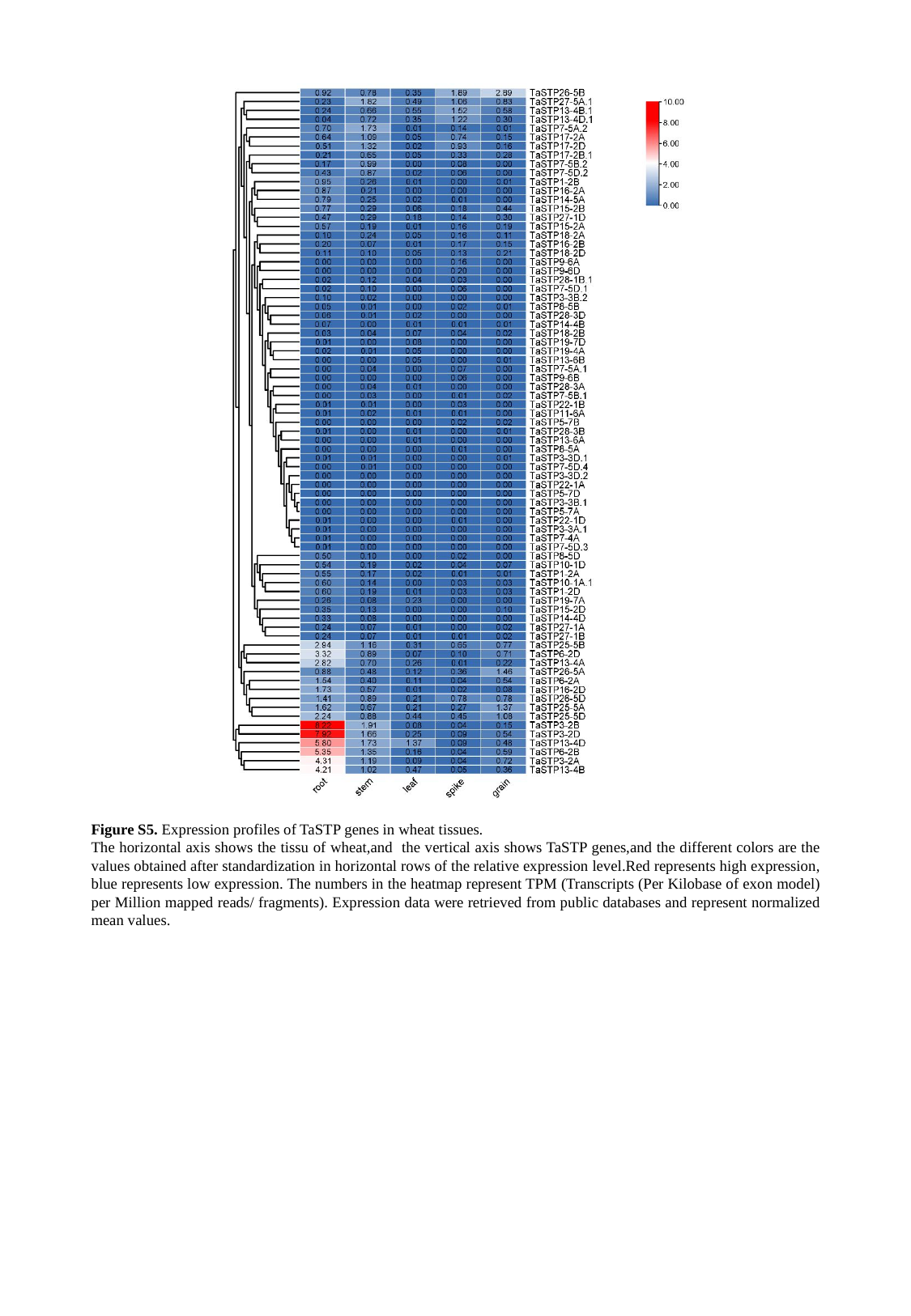

Figure S5. Expression profiles of TaSTP genes in wheat tissues.
The horizontal axis shows the tissu of wheat,and the vertical axis shows TaSTP genes,and the different colors are the values obtained after standardization in horizontal rows of the relative expression level.Red represents high expression, blue represents low expression. The numbers in the heatmap represent TPM (Transcripts (Per Kilobase of exon model) per Million mapped reads/ fragments). Expression data were retrieved from public databases and represent normalized mean values.

## Slide 9
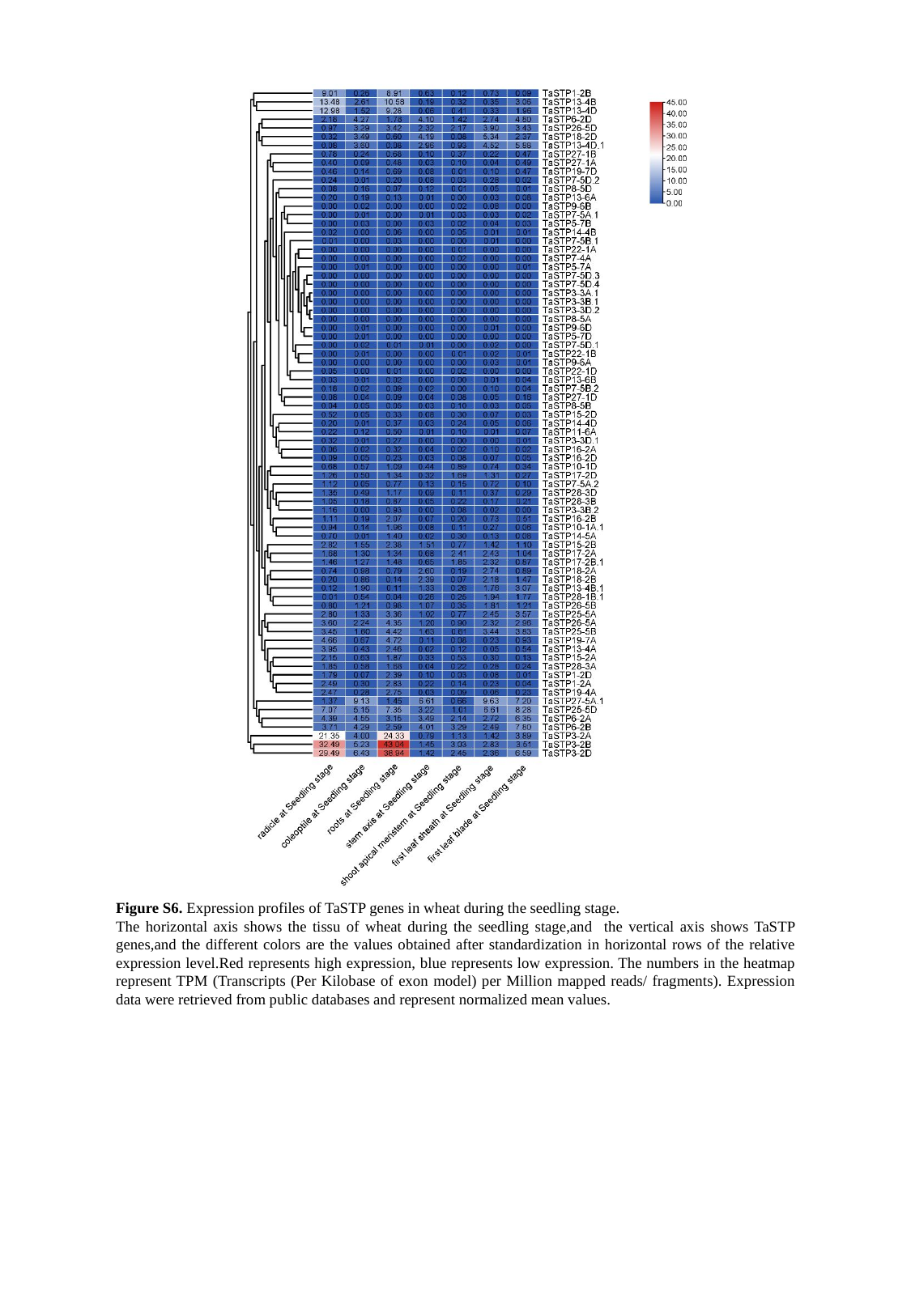

Figure S6. Expression profiles of TaSTP genes in wheat during the seedling stage.
The horizontal axis shows the tissu of wheat during the seedling stage,and the vertical axis shows TaSTP genes,and the different colors are the values obtained after standardization in horizontal rows of the relative expression level.Red represents high expression, blue represents low expression. The numbers in the heatmap represent TPM (Transcripts (Per Kilobase of exon model) per Million mapped reads/ fragments). Expression data were retrieved from public databases and represent normalized mean values.

## Slide 10
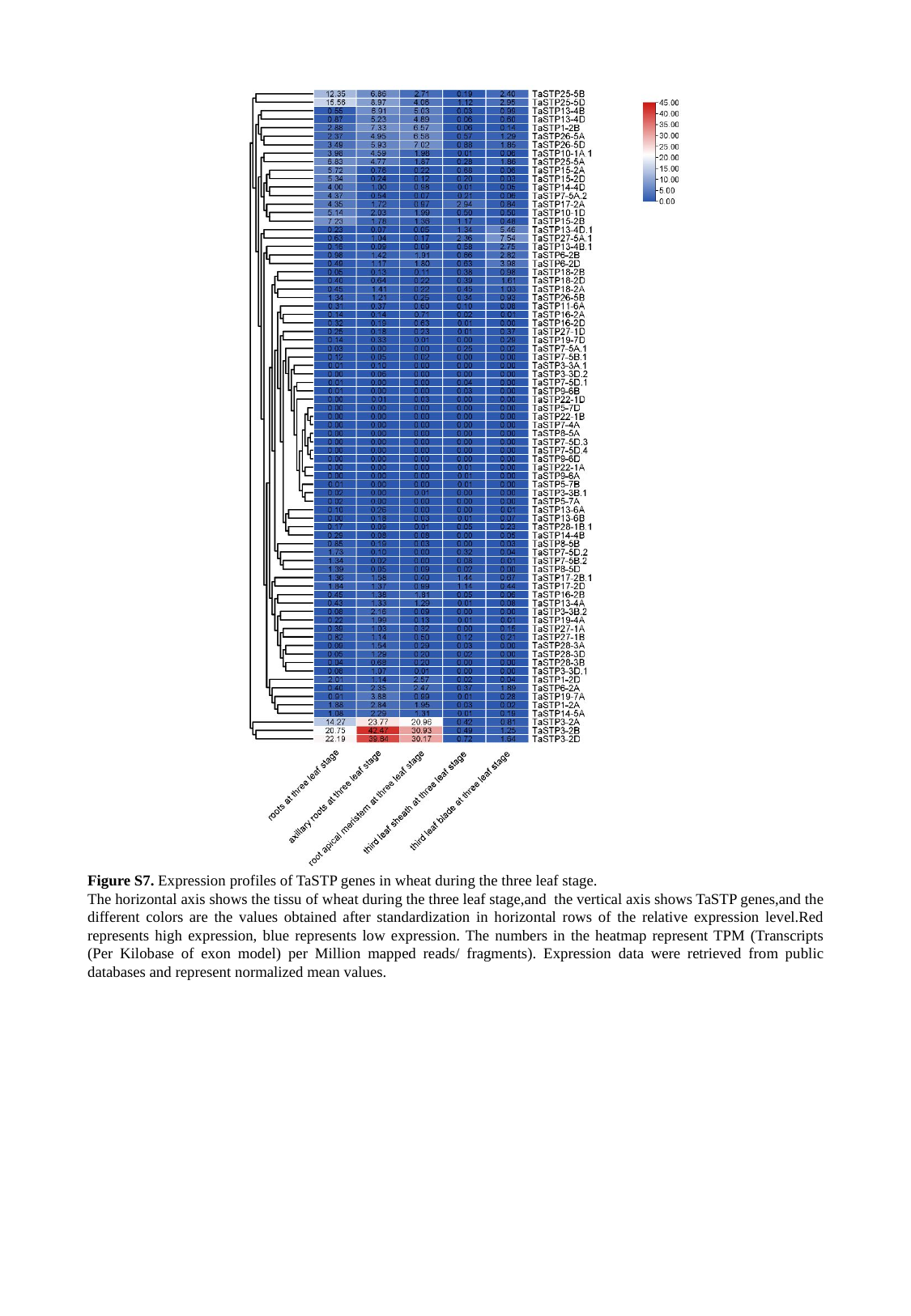

Figure S7. Expression profiles of TaSTP genes in wheat during the three leaf stage.
The horizontal axis shows the tissu of wheat during the three leaf stage,and the vertical axis shows TaSTP genes,and the different colors are the values obtained after standardization in horizontal rows of the relative expression level.Red represents high expression, blue represents low expression. The numbers in the heatmap represent TPM (Transcripts (Per Kilobase of exon model) per Million mapped reads/ fragments). Expression data were retrieved from public databases and represent normalized mean values.

## Slide 11
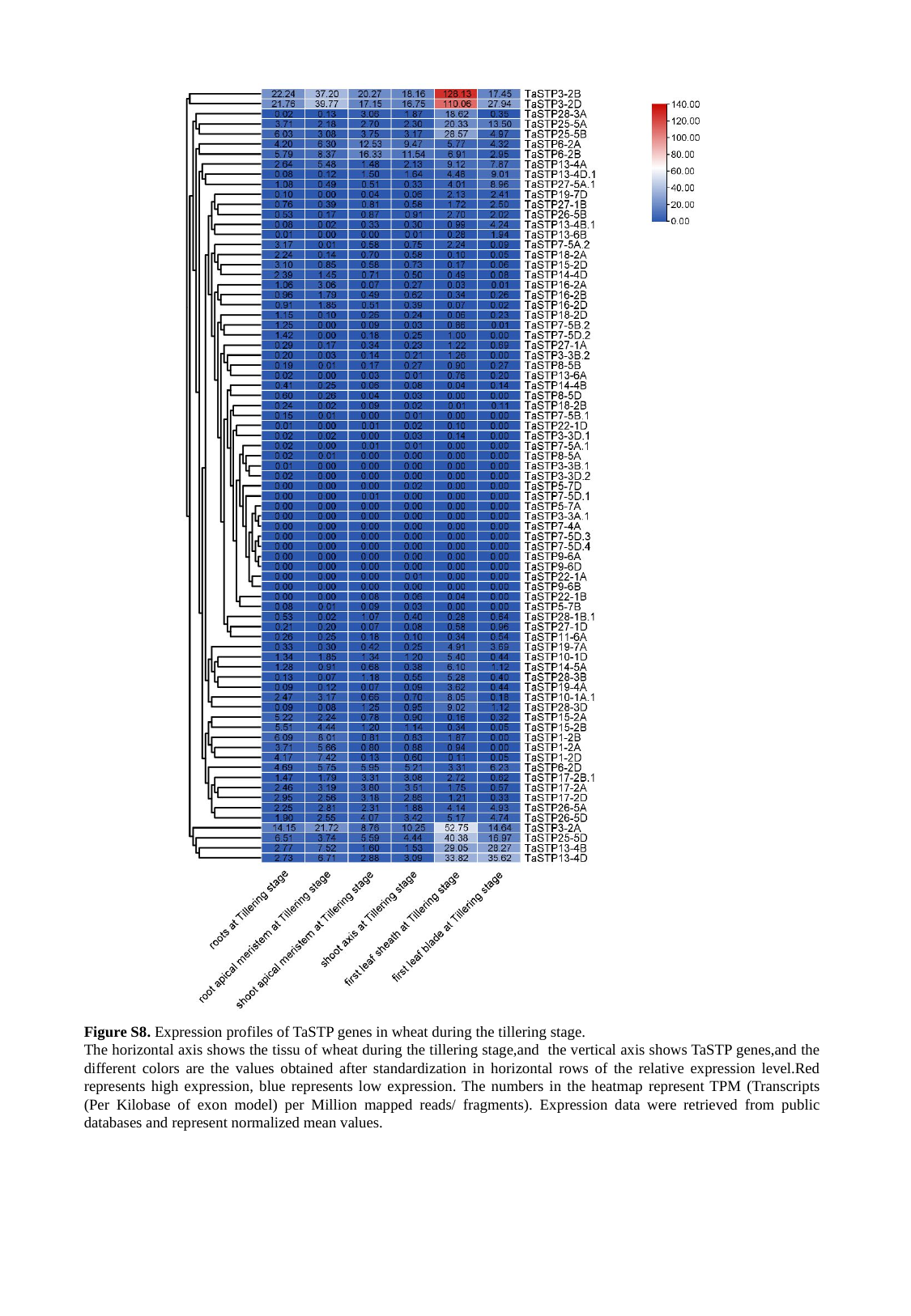

Figure S8. Expression profiles of TaSTP genes in wheat during the tillering stage.
The horizontal axis shows the tissu of wheat during the tillering stage,and the vertical axis shows TaSTP genes,and the different colors are the values obtained after standardization in horizontal rows of the relative expression level.Red represents high expression, blue represents low expression. The numbers in the heatmap represent TPM (Transcripts (Per Kilobase of exon model) per Million mapped reads/ fragments). Expression data were retrieved from public databases and represent normalized mean values.

## Slide 12
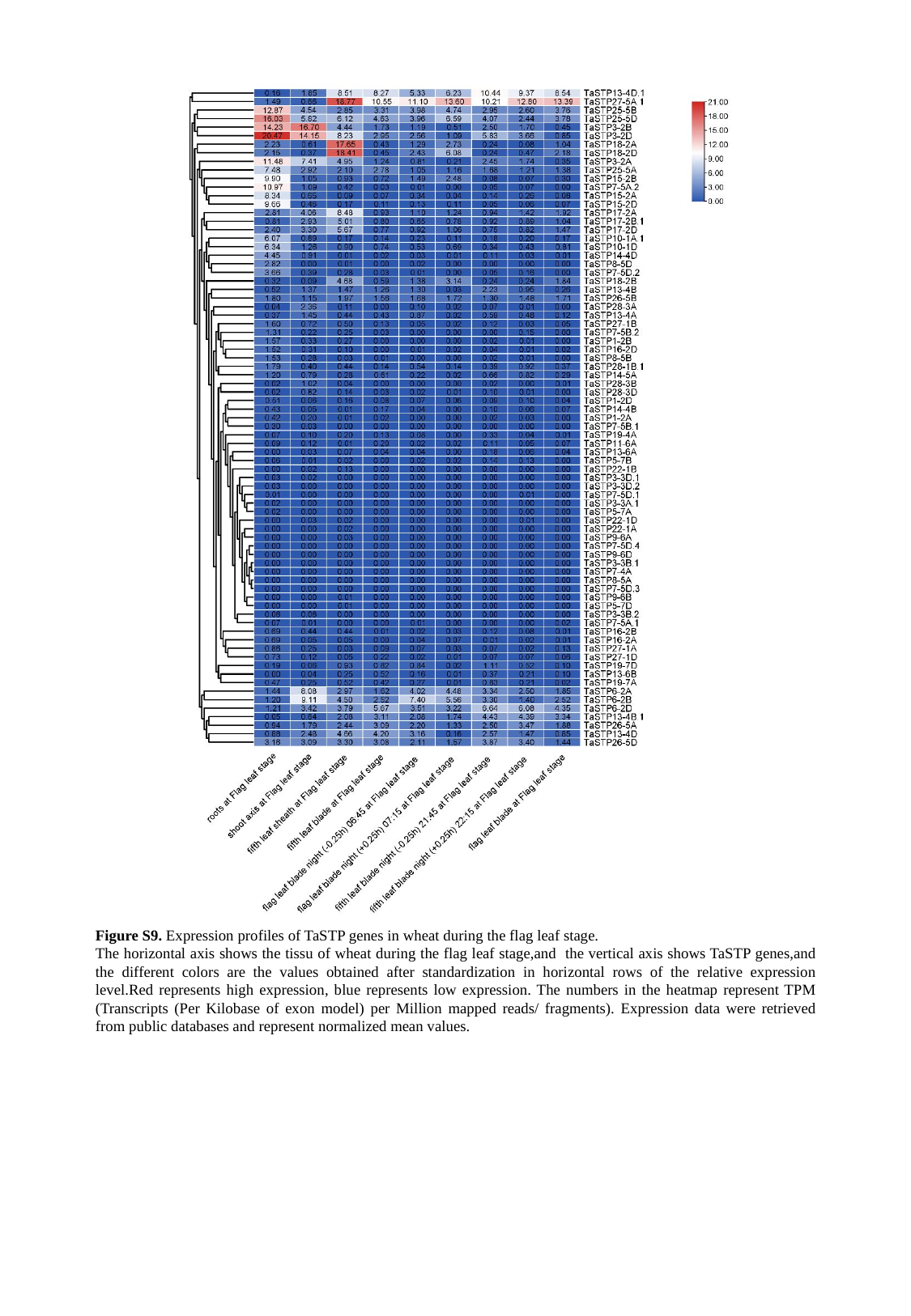

Figure S9. Expression profiles of TaSTP genes in wheat during the flag leaf stage.
The horizontal axis shows the tissu of wheat during the flag leaf stage,and the vertical axis shows TaSTP genes,and the different colors are the values obtained after standardization in horizontal rows of the relative expression level.Red represents high expression, blue represents low expression. The numbers in the heatmap represent TPM (Transcripts (Per Kilobase of exon model) per Million mapped reads/ fragments). Expression data were retrieved from public databases and represent normalized mean values.

## Slide 13
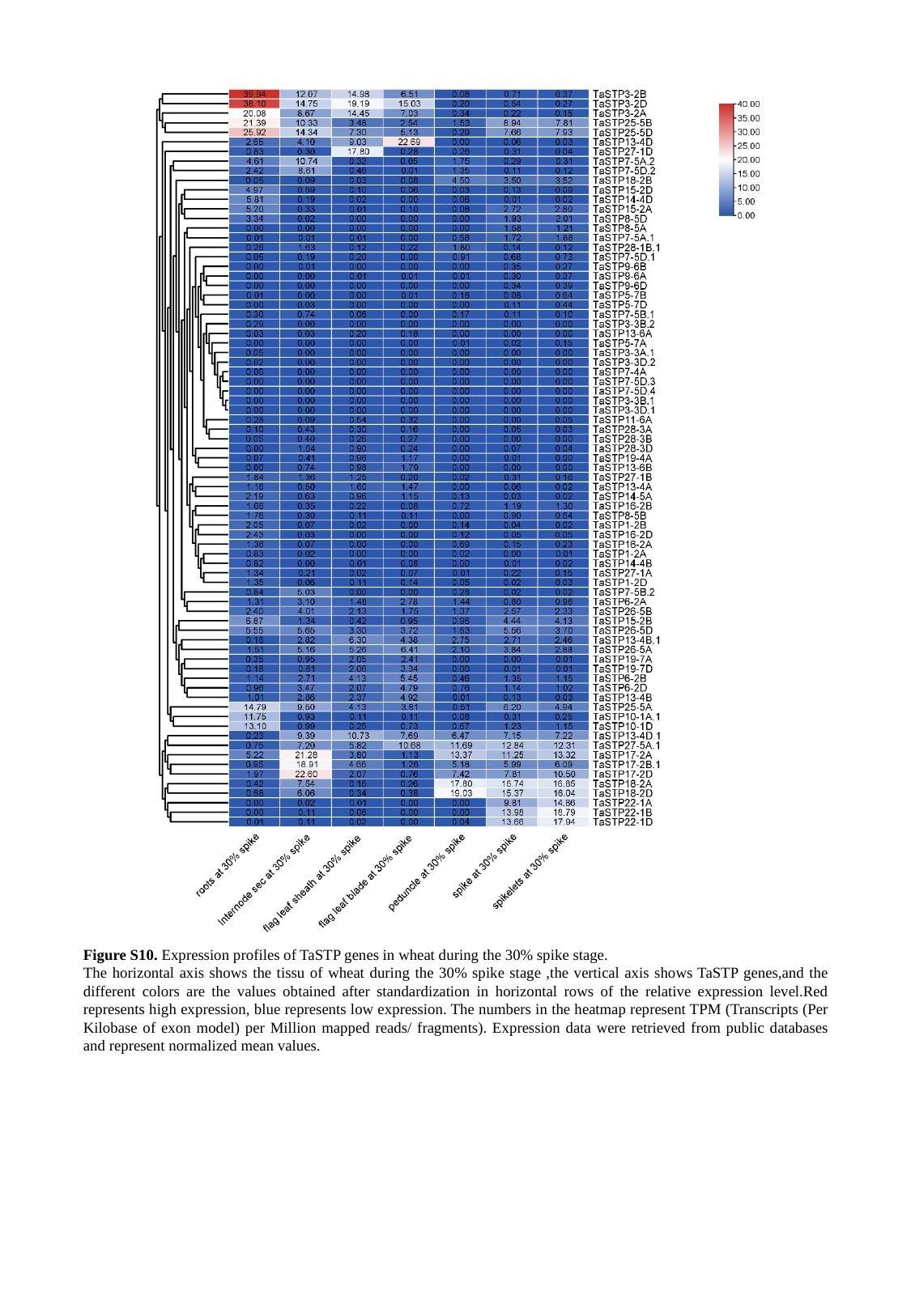

Figure S10. Expression profiles of TaSTP genes in wheat during the 30% spike stage.
The horizontal axis shows the tissu of wheat during the 30% spike stage ,the vertical axis shows TaSTP genes,and the different colors are the values obtained after standardization in horizontal rows of the relative expression level.Red represents high expression, blue represents low expression. The numbers in the heatmap represent TPM (Transcripts (Per Kilobase of exon model) per Million mapped reads/ fragments). Expression data were retrieved from public databases and represent normalized mean values.

## Slide 14
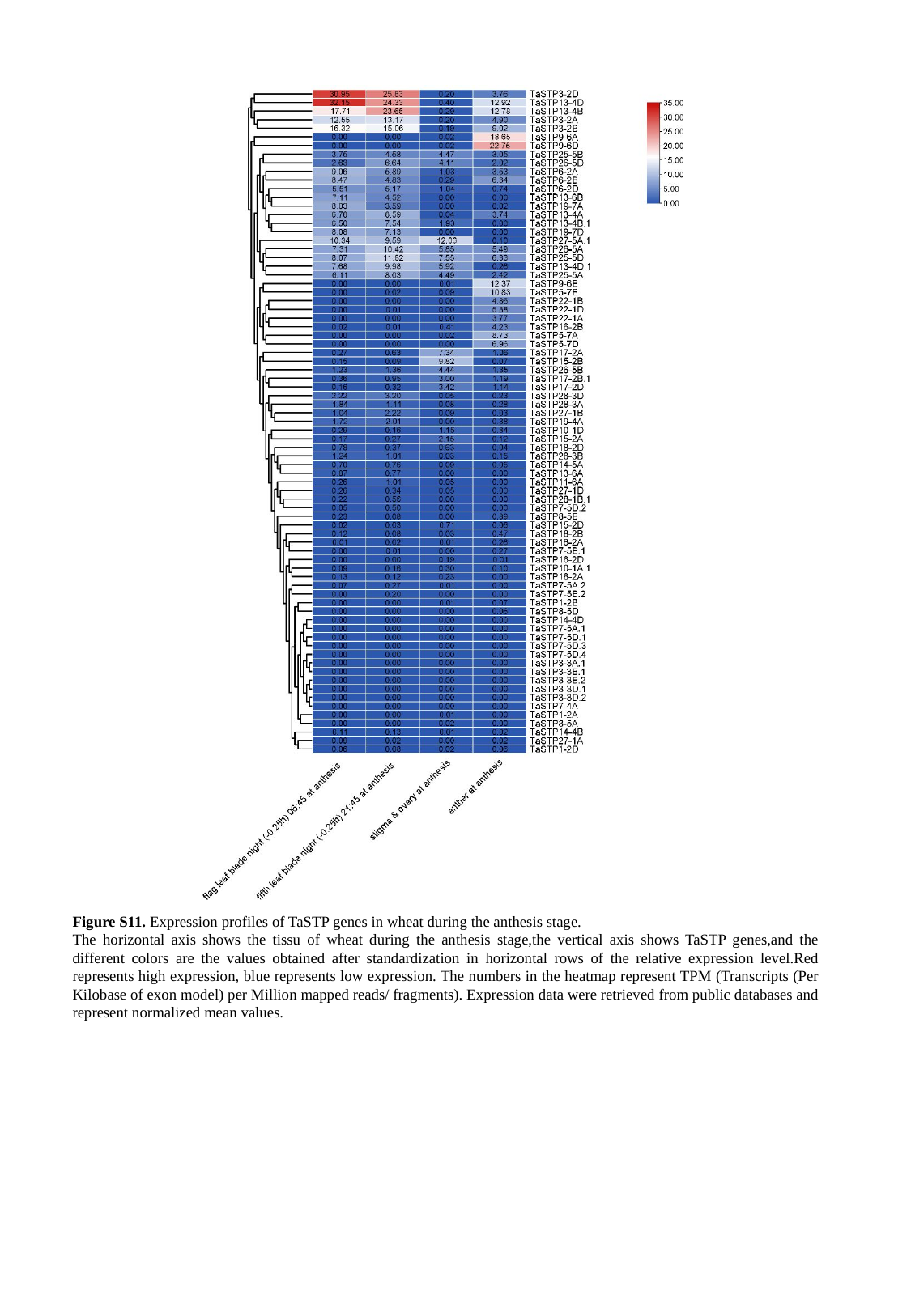

Figure S11. Expression profiles of TaSTP genes in wheat during the anthesis stage.
The horizontal axis shows the tissu of wheat during the anthesis stage,the vertical axis shows TaSTP genes,and the different colors are the values obtained after standardization in horizontal rows of the relative expression level.Red represents high expression, blue represents low expression. The numbers in the heatmap represent TPM (Transcripts (Per Kilobase of exon model) per Million mapped reads/ fragments). Expression data were retrieved from public databases and represent normalized mean values.

## Slide 15
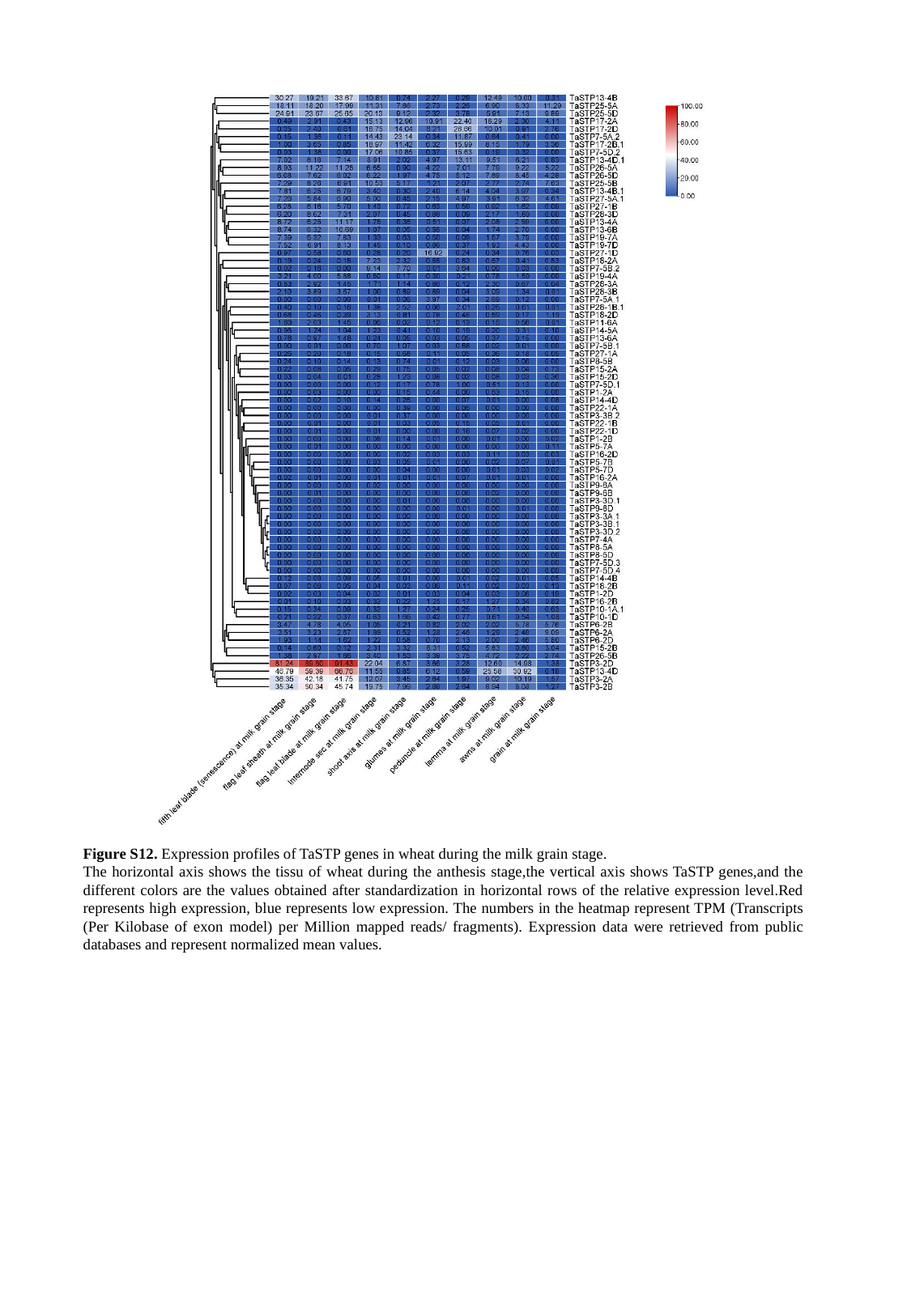

Figure S12. Expression profiles of TaSTP genes in wheat during the milk grain stage.
The horizontal axis shows the tissu of wheat during the anthesis stage,the vertical axis shows TaSTP genes,and the different colors are the values obtained after standardization in horizontal rows of the relative expression level.Red represents high expression, blue represents low expression. The numbers in the heatmap represent TPM (Transcripts (Per Kilobase of exon model) per Million mapped reads/ fragments). Expression data were retrieved from public databases and represent normalized mean values.

## Slide 16
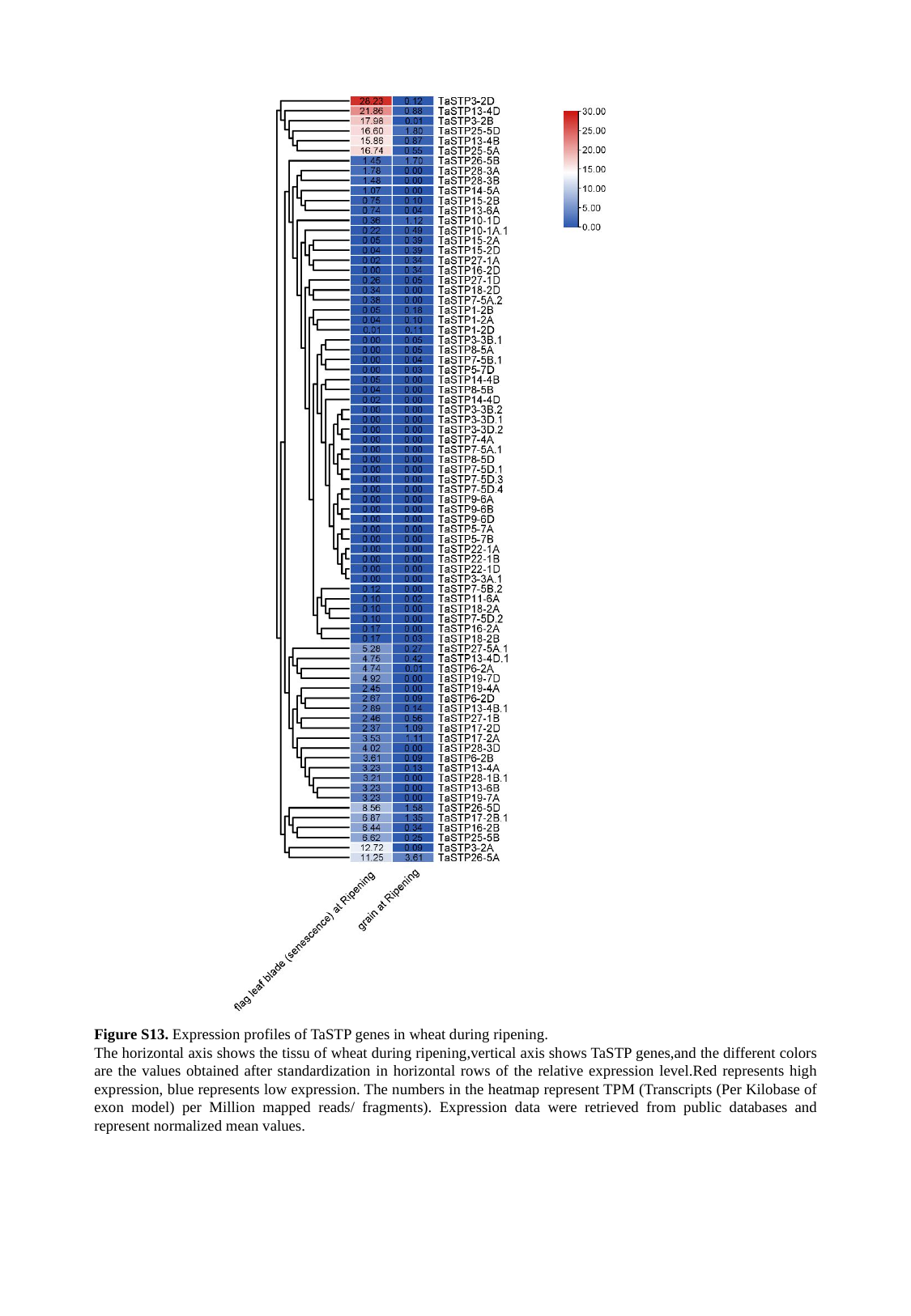

Figure S13. Expression profiles of TaSTP genes in wheat during ripening.
The horizontal axis shows the tissu of wheat during ripening,vertical axis shows TaSTP genes,and the different colors are the values obtained after standardization in horizontal rows of the relative expression level.Red represents high expression, blue represents low expression. The numbers in the heatmap represent TPM (Transcripts (Per Kilobase of exon model) per Million mapped reads/ fragments). Expression data were retrieved from public databases and represent normalized mean values.

## Slide 17
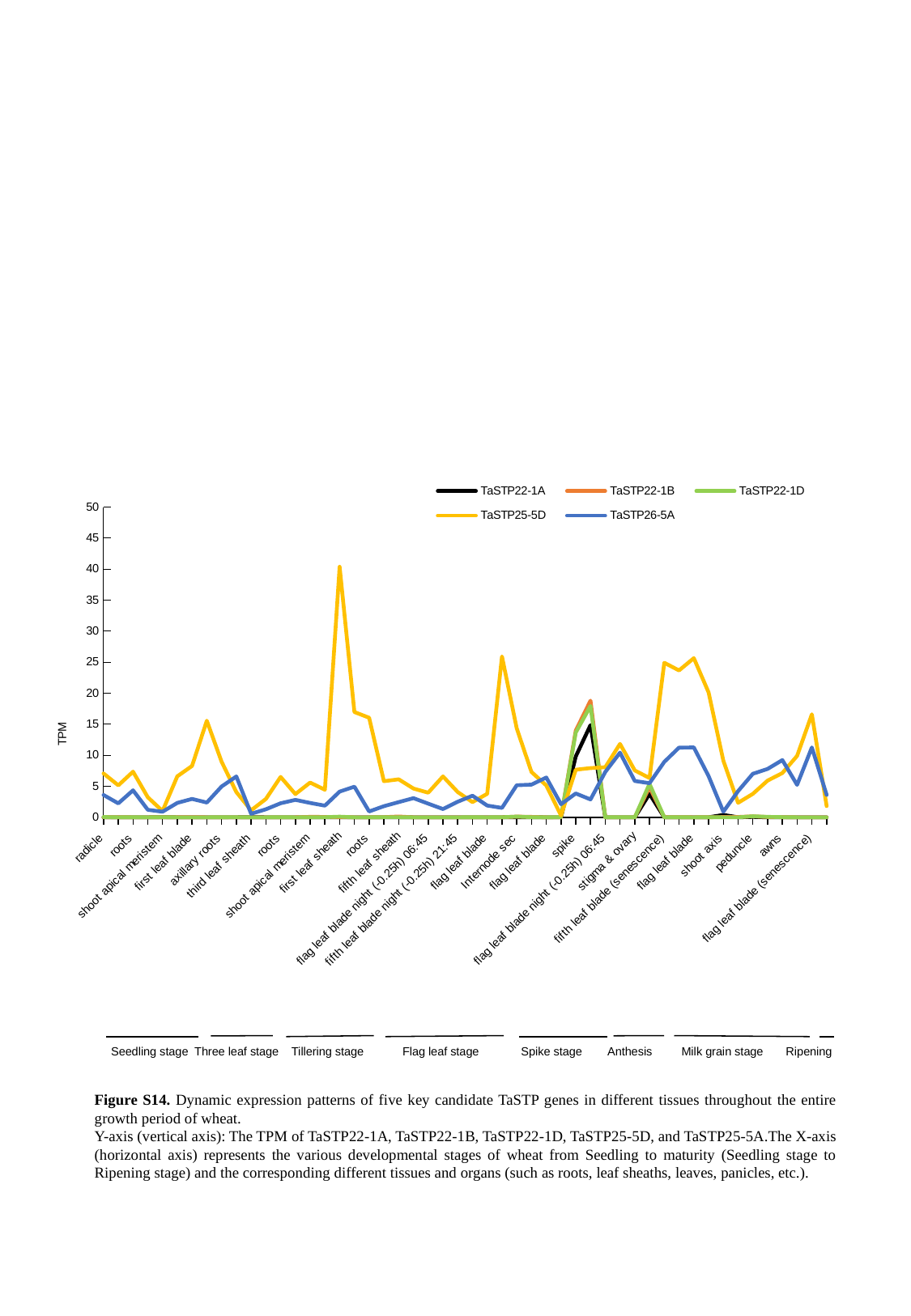

### Chart
| Category | TaSTP22-1A | TaSTP22-1B | TaSTP22-1D | TaSTP25-5D | TaSTP26-5A |
|---|---|---|---|---|---|
| radicle | 0.0 | 0.0 | 0.045632 | 7.06647 | 3.5967 |
| coleoptile | 0.0 | 0.008497 | 0.0 | 5.14911 | 2.23761 |
| roots | 0.0 | 0.0 | 0.013555 | 7.35347 | 4.34907 |
| stem axis | 0.0 | 0.0 | 0.0 | 3.22185 | 1.20339 |
| shoot apical meristem | 0.007766 | 0.014068 | 0.02494 | 1.01457 | 0.899126 |
| first leaf sheath | 0.0 | 0.017295 | 0.0 | 6.60772 | 2.32063 |
| first leaf blade | 0.0 | 0.014849 | 0.0 | 8.27643 | 2.96058 |
| roots | 0.0 | 0.0 | 0.0 | 15.5616 | 2.36976 |
| axillary roots | 0.0 | 0.0 | 0.012632 | 8.96995 | 4.9532 |
| root apical meristem | 0.0 | 0.0 | 0.030134 | 4.06127 | 6.58341 |
| third leaf sheath | 0.007958 | 0.0 | 0.0 | 1.12202 | 0.567694 |
| third leaf blade | 0.0 | 0.0 | 0.0 | 2.95294 | 1.29046 |
| roots | 0.0 | 0.0 | 0.010728 | 6.50821 | 2.25275 |
| root apical meristem | 0.0 | 0.0 | 0.0 | 3.74058 | 2.8102 |
| shoot apical meristem | 0.0 | 0.075637 | 0.005305 | 5.59326 | 2.3107 |
| shoot axis | 0.006628 | 0.056786 | 0.02046 | 4.44075 | 1.87504 |
| first leaf sheath | 0.0 | 0.041557 | 0.103229 | 40.3804 | 4.1422 |
| first leaf blade | 0.0 | 0.0 | 0.0 | 16.9688 | 4.93386 |
| roots | 0.0 | 0.0 | 0.0 | 16.0344 | 0.936985 |
| shoot axis | 0.0 | 0.01572 | 0.027955 | 5.81671 | 1.78858 |
| fifth leaf sheath | 0.022891 | 0.129528 | 0.020725 | 6.11614 | 2.4384 |
| fifth leaf blade | 0.0 | 0.0 | 0.0 | 4.63323 | 3.08771 |
| flag leaf blade night (-0.25h) 06:45 | 0.0 | 0.0 | 0.0 | 3.9606 | 2.19701 |
| flag leaf blade night (+0.25h) 07:15 | 0.0 | 0.0 | 0.0 | 6.58736 | 1.32633 |
| fifth leaf blade night (-0.25h) 21:45 | 0.0 | 0.0 | 0.0 | 4.0727 | 2.5015 |
| fifth leaf blade night (+0.25h) 22:15 | 0.0 | 0.0 | 0.006361 | 2.43855 | 3.47431 |
| flag leaf blade | 0.0 | 0.0 | 0.0 | 3.7844 | 1.88491 |
| roots | 0.0 | 0.0 | 0.013661 | 25.9238 | 1.50776 |
| Internode sec | 0.016576 | 0.112998 | 0.113765 | 14.3402 | 5.16175 |
| flag leaf sheath | 0.006837 | 0.062744 | 0.021778 | 7.29942 | 5.26372 |
| flag leaf blade | 0.0 | 0.0 | 0.0 | 5.12783 | 6.41103 |
| peduncle | 0.0 | 0.0 | 0.041005 | 0.288675 | 2.10455 |
| spike | 9.8127 | 13.983 | 13.6615 | 7.66357 | 3.84076 |
| spikelets | 14.8603 | 18.7934 | 17.9368 | 7.92637 | 2.87835 |
| flag leaf blade night (-0.25h) 06:45 | 0.0 | 0.0 | 0.0 | 8.06518 | 7.30888 |
| fifth leaf blade night (-0.25h) 21:45 | 0.0 | 0.0 | 0.008289 | 11.8224 | 10.418 |
| stigma & ovary | 0.0 | 0.0 | 0.0 | 7.55109 | 5.84921 |
| anther | 3.76903 | 4.85637 | 5.38423 | 6.32891 | 5.49497 |
| fifth leaf blade (senescence) | 0.0 | 0.0 | 0.0 | 24.9088 | 8.92776 |
| flag leaf sheath | 0.0 | 0.013669 | 0.006754 | 23.6715 | 11.2244 |
| flag leaf blade | 0.0 | 0.0 | 0.0 | 25.6478 | 11.2538 |
| Internode sec | 0.0 | 0.011907 | 0.012287 | 20.1321 | 6.64825 |
| shoot axis | 0.385793 | 0.0309 | 0.0 | 9.12127 | 0.895731 |
| glumes | 0.0 | 0.046592 | 0.0 | 2.32431 | 4.21536 |
| peduncle | 0.056306 | 0.14913 | 0.161587 | 3.78006 | 7.0123 |
| lemma | 0.0 | 0.05049 | 0.073001 | 5.90649 | 7.79104 |
| awns | 0.0 | 0.007826 | 0.023581 | 7.12663 | 9.22251 |
| grain | 0.0 | 0.0 | 0.0 | 9.89396 | 5.224 |
| flag leaf blade (senescence) | 0.0 | 0.0 | 0.0 | 16.6028 | 11.2538 |
| grain | 0.0 | 0.0 | 0.0 | 1.80106 | 3.61242 |Seedling stage Three leaf stage Tillering stage Flag leaf stage Spike stage Anthesis Milk grain stage Ripening
Figure S14. Dynamic expression patterns of five key candidate TaSTP genes in different tissues throughout the entire growth period of wheat.
Y-axis (vertical axis): The TPM of TaSTP22-1A, TaSTP22-1B, TaSTP22-1D, TaSTP25-5D, and TaSTP25-5A.The X-axis (horizontal axis) represents the various developmental stages of wheat from Seedling to maturity (Seedling stage to Ripening stage) and the corresponding different tissues and organs (such as roots, leaf sheaths, leaves, panicles, etc.).

## Slide 18
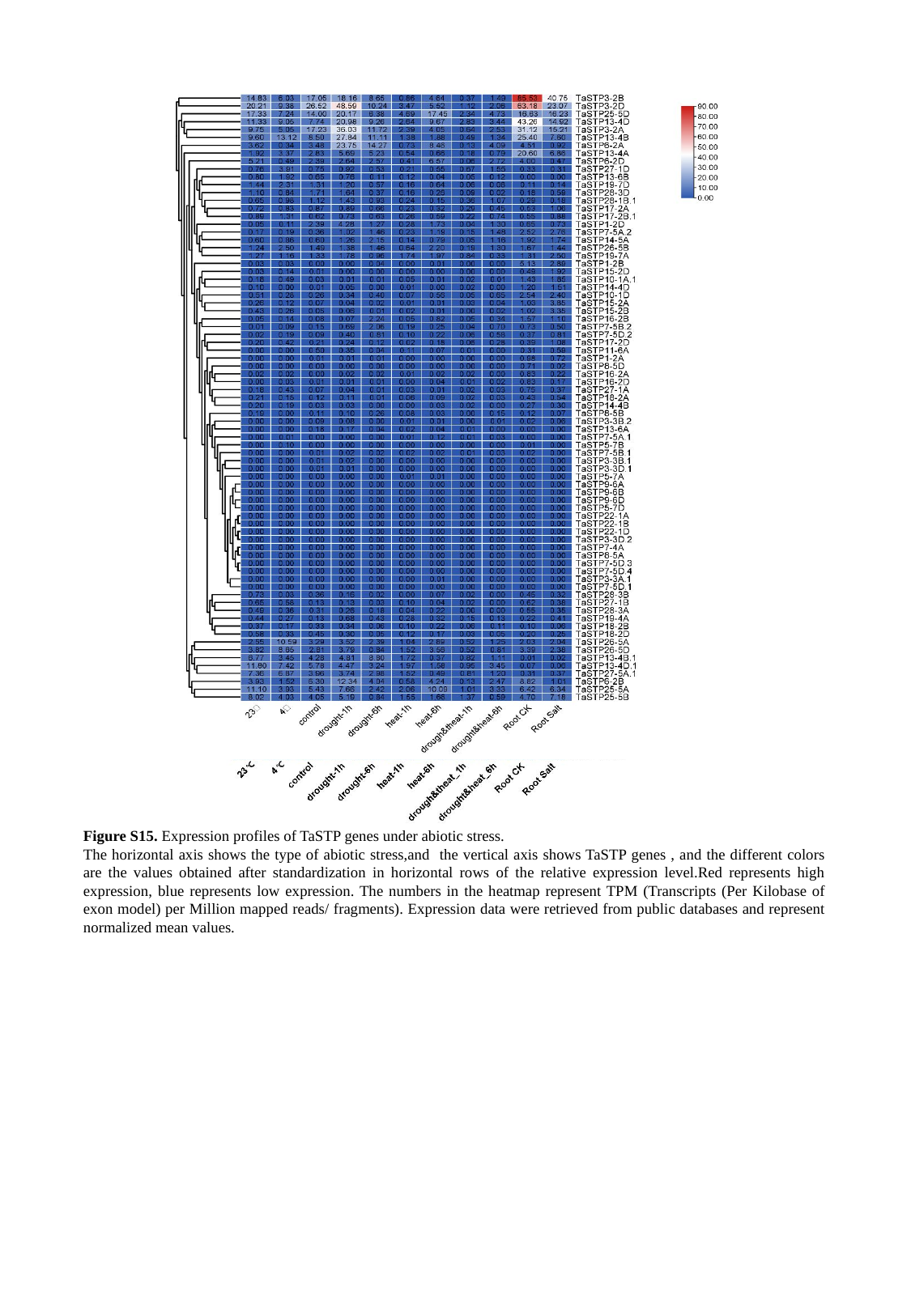

Figure S15. Expression profiles of TaSTP genes under abiotic stress.
The horizontal axis shows the type of abiotic stress,and the vertical axis shows TaSTP genes , and the different colors are the values obtained after standardization in horizontal rows of the relative expression level.Red represents high expression, blue represents low expression. The numbers in the heatmap represent TPM (Transcripts (Per Kilobase of exon model) per Million mapped reads/ fragments). Expression data were retrieved from public databases and represent normalized mean values.

## Slide 19
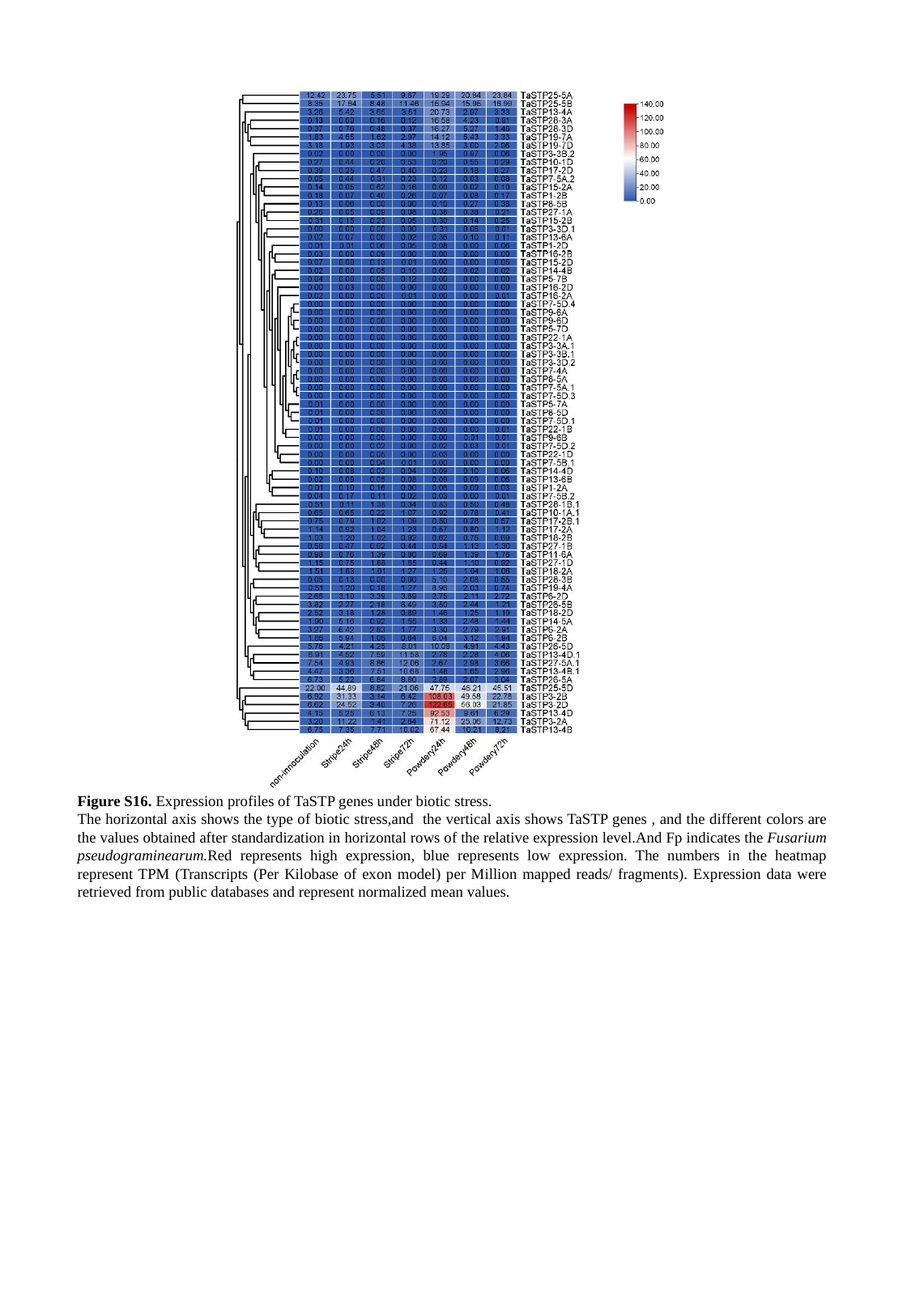

Figure S16. Expression profiles of TaSTP genes under biotic stress.
The horizontal axis shows the type of biotic stress,and the vertical axis shows TaSTP genes , and the different colors are the values obtained after standardization in horizontal rows of the relative expression level.And Fp indicates the Fusarium pseudograminearum.Red represents high expression, blue represents low expression. The numbers in the heatmap represent TPM (Transcripts (Per Kilobase of exon model) per Million mapped reads/ fragments). Expression data were retrieved from public databases and represent normalized mean values.

## Slide 20
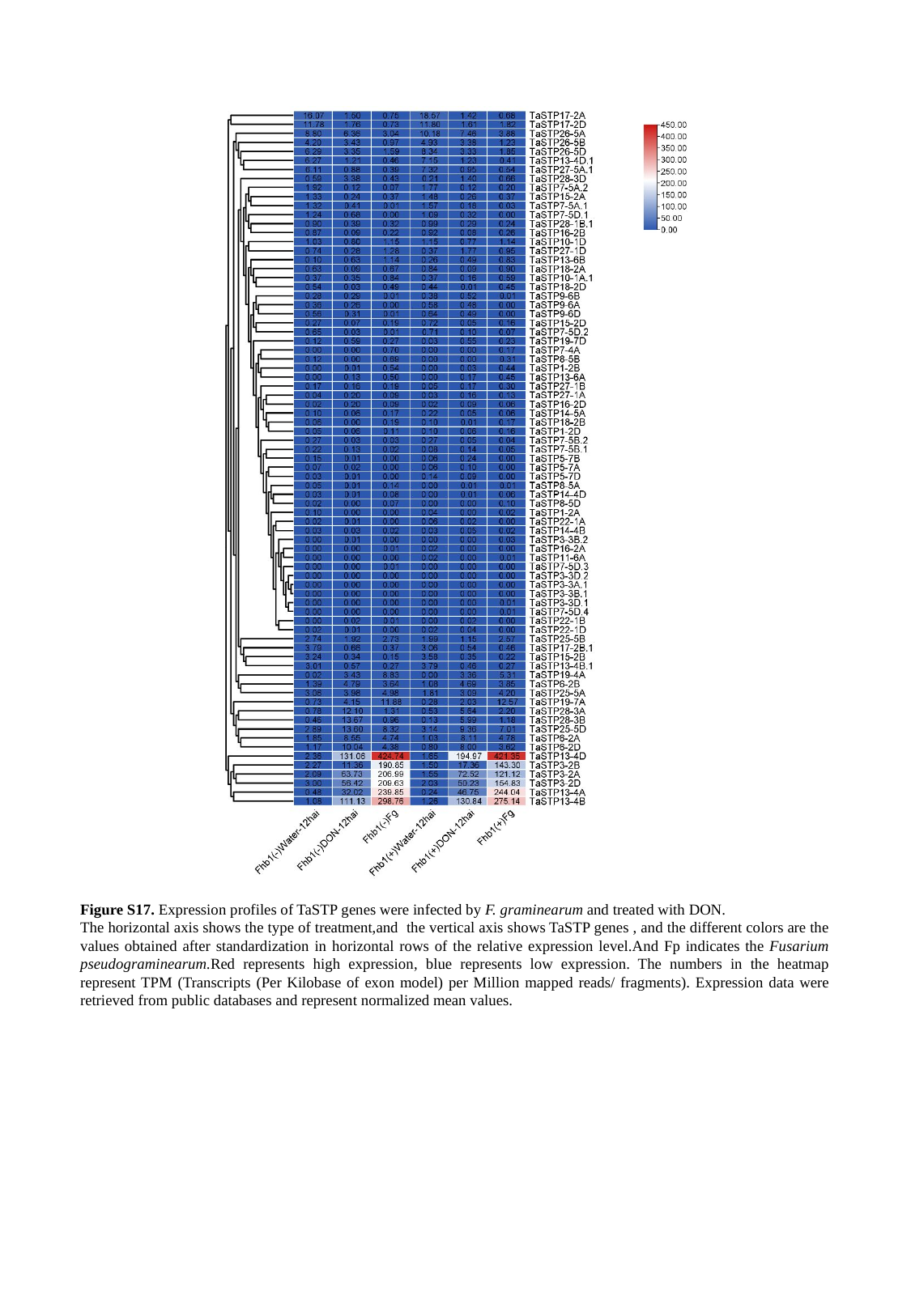

Figure S17. Expression profiles of TaSTP genes were infected by F. graminearum and treated with DON.
The horizontal axis shows the type of treatment,and the vertical axis shows TaSTP genes , and the different colors are the values obtained after standardization in horizontal rows of the relative expression level.And Fp indicates the Fusarium pseudograminearum.Red represents high expression, blue represents low expression. The numbers in the heatmap represent TPM (Transcripts (Per Kilobase of exon model) per Million mapped reads/ fragments). Expression data were retrieved from public databases and represent normalized mean values.

## Slide 21
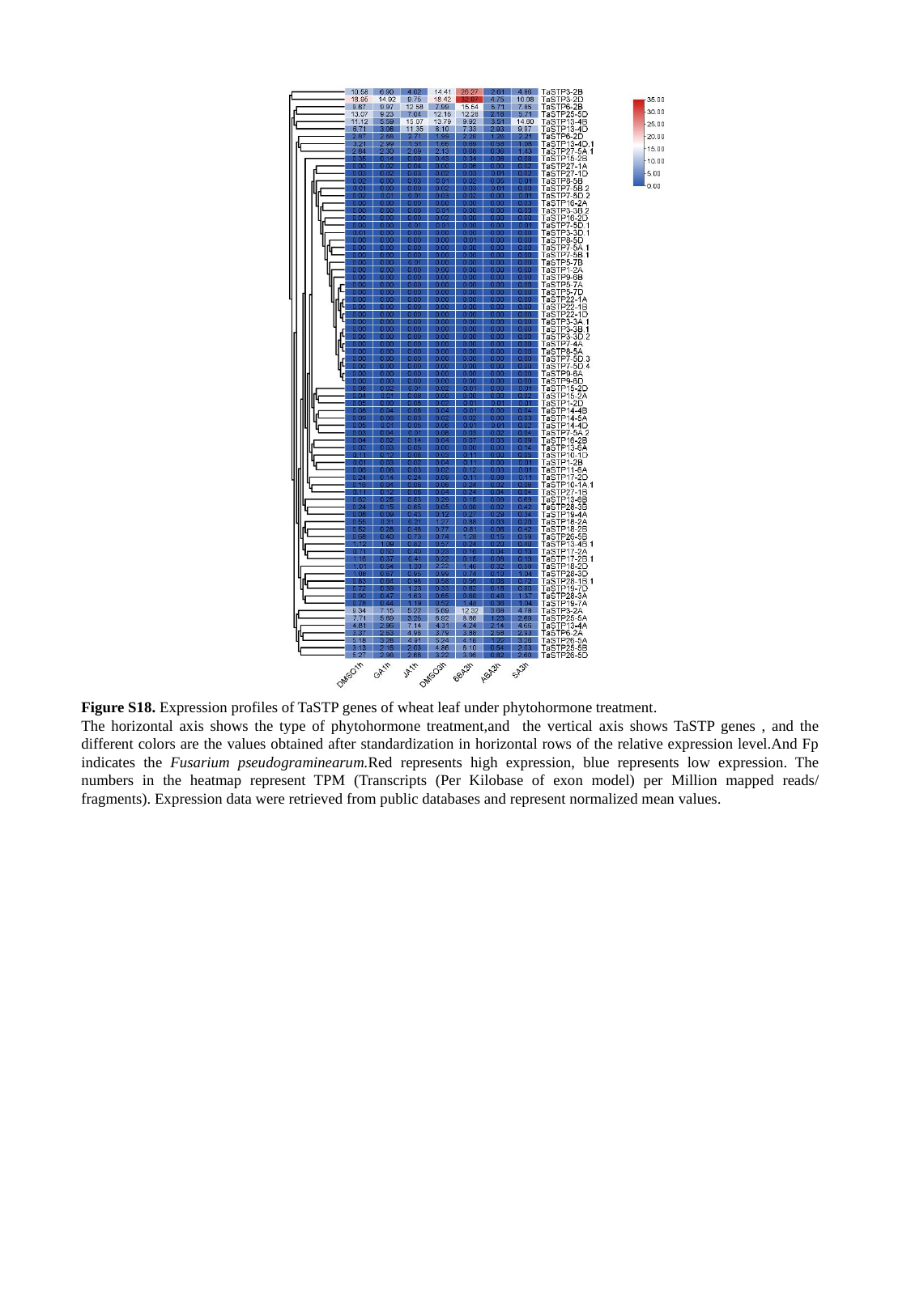

Figure S18. Expression profiles of TaSTP genes of wheat leaf under phytohormone treatment.
The horizontal axis shows the type of phytohormone treatment,and the vertical axis shows TaSTP genes , and the different colors are the values obtained after standardization in horizontal rows of the relative expression level.And Fp indicates the Fusarium pseudograminearum.Red represents high expression, blue represents low expression. The numbers in the heatmap represent TPM (Transcripts (Per Kilobase of exon model) per Million mapped reads/ fragments). Expression data were retrieved from public databases and represent normalized mean values.

## Slide 22
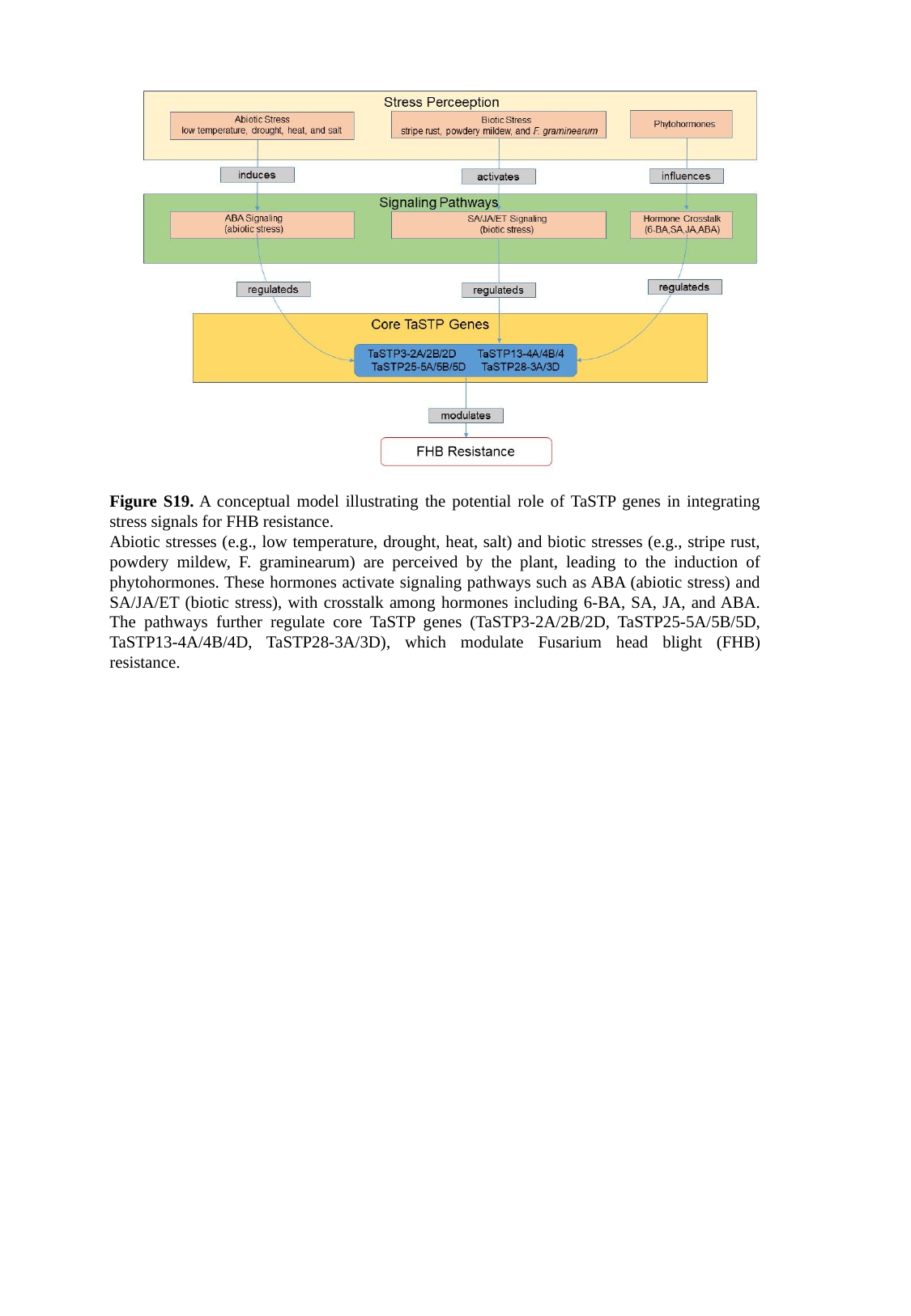

Figure S19. A conceptual model illustrating the potential role of TaSTP genes in integrating stress signals for FHB resistance.
Abiotic stresses (e.g., low temperature, drought, heat, salt) and biotic stresses (e.g., stripe rust, powdery mildew, F. graminearum) are perceived by the plant, leading to the induction of phytohormones. These hormones activate signaling pathways such as ABA (abiotic stress) and SA/JA/ET (biotic stress), with crosstalk among hormones including 6-BA, SA, JA, and ABA. The pathways further regulate core TaSTP genes (TaSTP3-2A/2B/2D, TaSTP25-5A/5B/5D, TaSTP13-4A/4B/4D, TaSTP28-3A/3D), which modulate Fusarium head blight (FHB) resistance.

## Slide 23
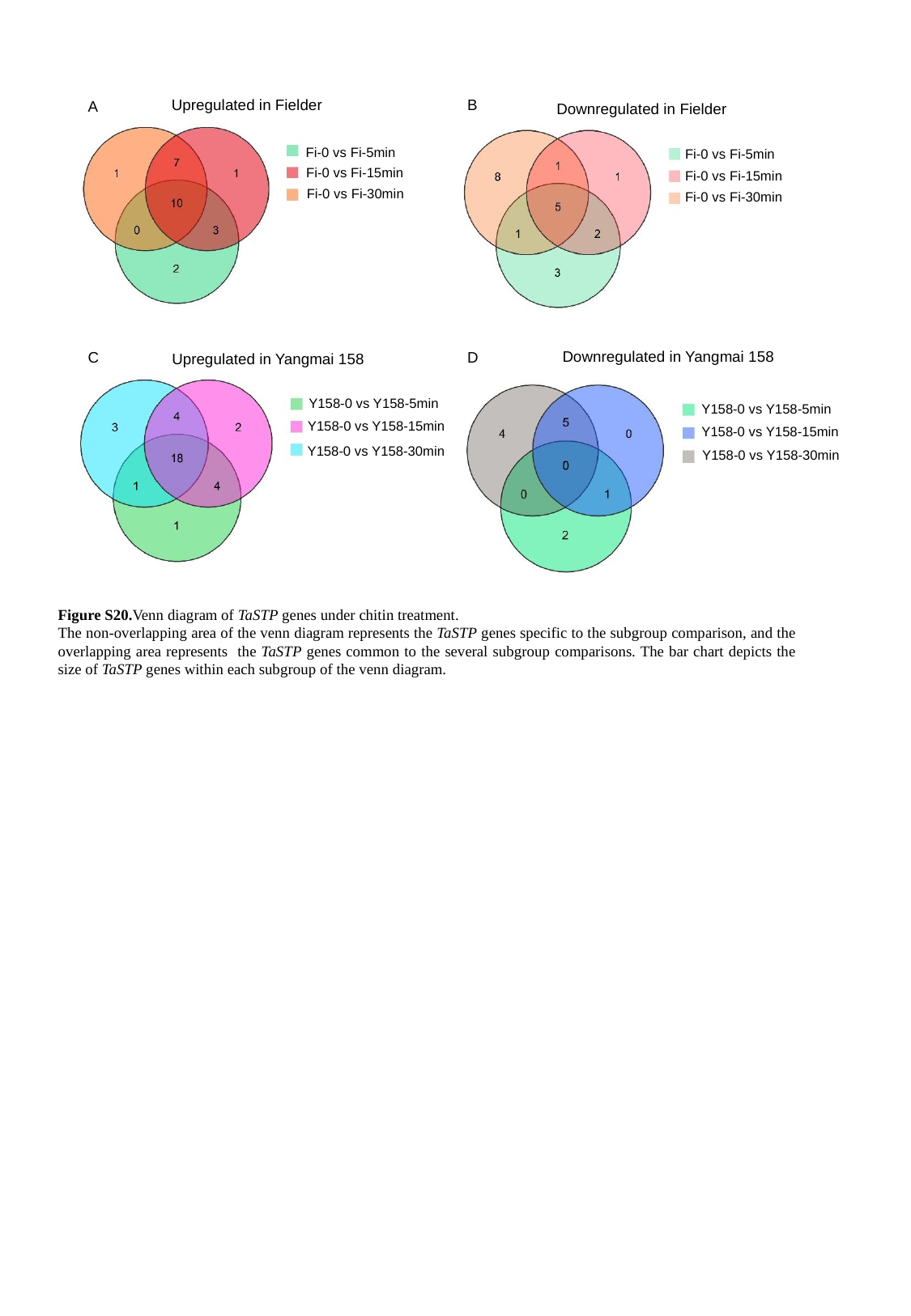

Upregulated in Fielder
B
A
Downregulated in Fielder
Fi-0 vs Fi-5min
Fi-0 vs Fi-5min
Fi-0 vs Fi-15min
Fi-0 vs Fi-15min
Fi-0 vs Fi-30min
Fi-0 vs Fi-30min
Downregulated in Yangmai 158
C
D
Upregulated in Yangmai 158
Y158-0 vs Y158-5min
Y158-0 vs Y158-5min
Y158-0 vs Y158-15min
Y158-0 vs Y158-15min
Y158-0 vs Y158-30min
Y158-0 vs Y158-30min
Figure S20.Venn diagram of TaSTP genes under chitin treatment.
The non-overlapping area of the venn diagram represents the TaSTP genes specific to the subgroup comparison, and the overlapping area represents the TaSTP genes common to the several subgroup comparisons. The bar chart depicts the size of TaSTP genes within each subgroup of the venn diagram.

## Slide 24
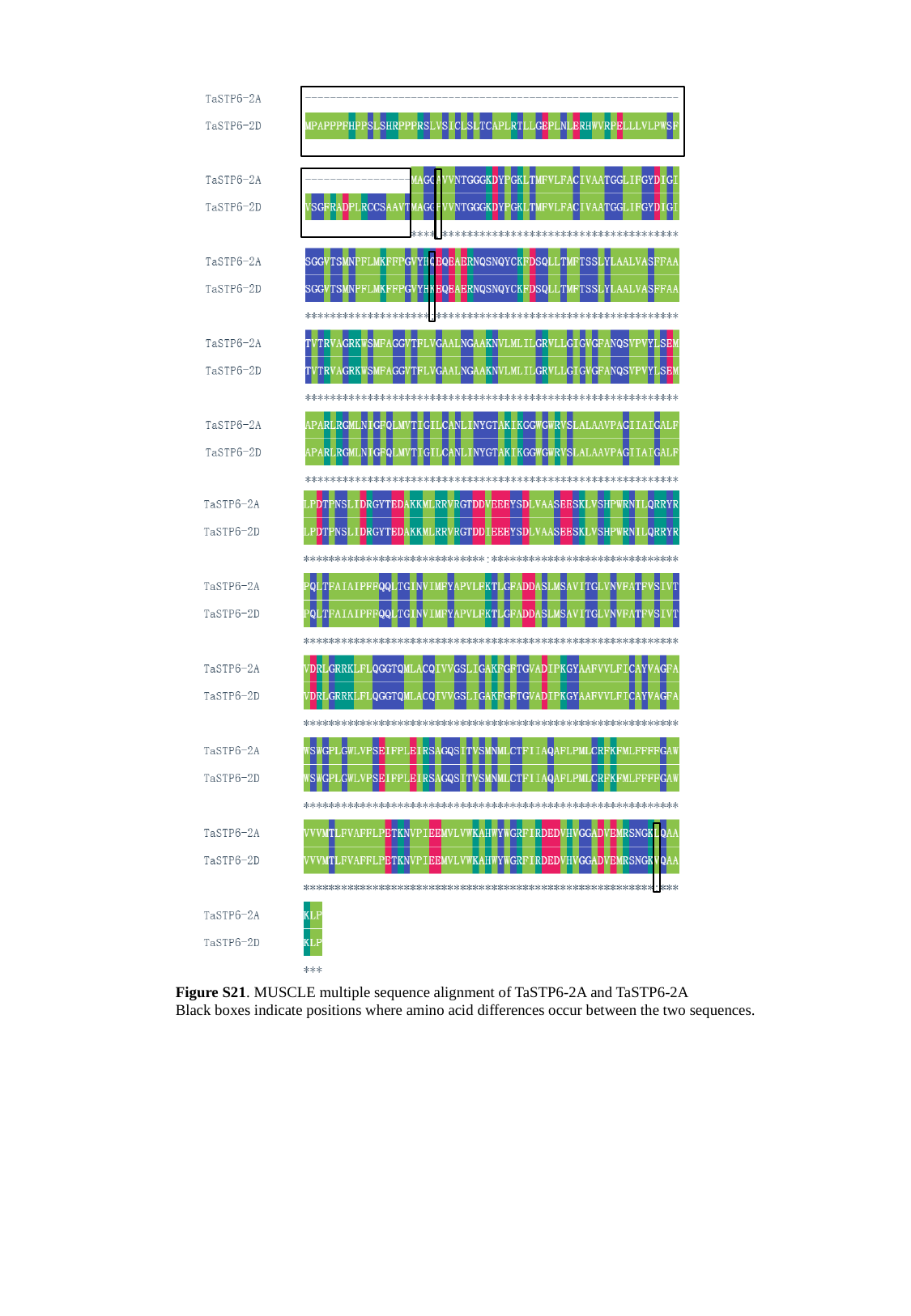

Figure S21. MUSCLE multiple sequence alignment of TaSTP6-2A and TaSTP6-2A
Black boxes indicate positions where amino acid differences occur between the two sequences.
