# Supplementary material for: Genome-Wide Identification and Expression Profiling of Sugar Transport Protein Response to Fusarium Head Blight in Wheat (Triticum aestivum L.)
Source: Plants (Basel). 2025 Sep 25;14(19):2976. doi: 10.3390/plants14192976 (PMC12526077; doi:10.3390/plants14192976)
Supplement: Supplementary file 1 [file plants-14-02976-s001.zip › List of supplementary materials.pdf]

**The list of supplementary materials:**

Figure S1: Phylogenetic tree of the putative TaSTP proteins;

Figure S2: Conserved domain analysis of TaSTP genes family members;

Figure S3: Prediction of transmembrane helices in the TaSTP genes family;

Figure S4: Chromosomal distribution of the sugar transporter gene family in wheat;

Figure S5: Expression profiles of TaSTP genes in wheat tissues;

Figure S6: Expression profiles of TaSTP genes in wheat during the seedling stage;

Figure S7: Expression profiles of TaSTP genes in wheat during the three leaf stage;

Figure S8: Expression profiles of TaSTP genes in wheat during the tillering stage;

Figure S9: Expression profiles of TaSTP genes in wheat during the flag leaf stage;

Figure S10: Expression profiles of TaSTP genes in wheat during the 30% spike stage;

Figure S11: Expression profiles of TaSTP genes in wheat during the anthesis stage;

Figure S12: Expression profiles of TaSTP genes in wheat during the milk grain stage;

Figure S13: Expression profiles of TaSTP genes in wheat during ripening;

Figure S14: Expression profiles of TaSTP genes under abiotic stress;

FigureS15: Dynamic expression patterns of five key candidate TaSTP genes in different tissues throughout the entire growth period of wheat;

Figure S16: Expression profiles of TaSTP genes under biotic stress;

Figure S17: Expression profiles of TaSTP genes were infected by F. graminearum and treated with DON;

Figure S18: Expression profiles of TaSTP genes of wheat leaf under phytohormone treatment;

Figure S19. A conceptual model illustrating the potential role of TaSTP genes in integrating stress signals for FHB resistance;

Figure S20:Venn diagram of TaSTP genes under chitin treatment;

Figure S21. MUSCLE multiple sequence alignment of TaSTP6-2A and TaSTP6-2A;

Table S1 : The results of conserved domain detection using the NCBI Batch CD-Search Tool;

Table S2. Information about the TaSTP genes in wheat;

Table S3: Named of TaSTP genes in wheat;

Table S4:The results of subcellular localization prediction of TaSTP genes;

Table S5: The results of transmembrane helices prediction of TaSTP genes;

Table S6: Chromosomal distribution and physical positions of TaSTP genes in wheat;

Table S7. The PSSs (%) for the Fielder and Yangmai 158 tested in 2024 and 2025 field experiments;

Table S8: List of TaSTP genes by chitin treatment using RT-qPCR;

Table S9: The relative expression levels analysis of TaSTP genes under chitin treatment;

Table S10: The relative expression levels analysis of TaSTP genes under F. graminearum infection;

Table S11: The relative expression levels analysis of TaSTP genes under DON treatment;

Table S12: The amino acid sequences of the TaSTP6-2A and TaSTP6-2D;

Table S13: Summary table of TaSTP genes validation results using RT-qPCR;

Table S14: The list of primer for RT-qPCR.
