# Supplementary material for: Genome-Wide Identification and Expression Profiling of Sugar Transport Protein Response to Fusarium Head Blight in Wheat (Triticum aestivum L.)
Source: Plants (Basel). 2025 Sep 25;14(19):2976. doi: 10.3390/plants14192976 (PMC12526077; doi:10.3390/plants14192976)
Supplement: Supplementary file 1 [file plants-14-02976-s001.zip › Table S12.pdf]

Table S12. The amino acid sequences of the TaSTP6-2A and TaSTP6-2D.

Gene Name	Amino acid sequence
>TaSTP6-2A	MAGGAVVNTGGGKDYPGKLTMFVLFACIVAATGGLIFGYDIGISGGVTSMNPFLMKFFPGV YHQEQEAERNQSNQYCKFDSQLLTMFTSSLYLAALVASFFAATVTRVAGRKWSMFAGGVT FLVGAALNGAAKNVLMLILGRVLLGIGVGFANQSVPVYLSEMAPARLRGMLNIGFQLMVT IGILCANLINYGTAKIKGGWGWRVSLALAAVPAGIIAIGALFLPDTPNSLIDRGYTEDAKKM LRRVRGTDDVEEEYSDLVAASEESKLVSHPWRNILQRRYRPQLTFAIAIPFFQQLTGINVIMF YAPVLFKTLGFADDASLMSAVITGLVNVFATFVSIVTVDRLGRRKLFLQGGTQMLACQIVV GSLIGAKFGFTGVADIPKGYAAFVVLFICAYVAGFAWSWGPLGWLVPSEIFPLEIRSAGQSIT VSMNMLCTFIIAQAFLPMLCRFKFMLFFFFGAWVVVMTLFVAFFLPETKNVPIEEMVLVWK AHWYWGRFIRDEDVHVGGADVEMRSNGKLQAAKLP
>TaSTP6-2D	MPAPPPFHPPSLSHRPPPRSLVSICLSLTCAPLRTLLGEPLNLERHWVRPELLLVLPWSFVSGF RADPLRCCSAAVTMAGGPVVNTGGGKDYPGKLTMFVLFACIVAATGGLIFGYDIGISGGVT SMNPFLMKFFPGVYHKEQEAERNQSNQYCKFDSQLLTMFTSSLYLAALVASFFAATVTRVA GRKWSMFAGGVTFLVGAALNGAAKNVLMLILGRVLLGIGVGFANQSVPVYLSEMAPARL RGMLNIGFQLMVTIGILCANLINYGTAKIKGGWGWRVSLALAAVPAGIIAIGALFLPDTPNS LIDRGYTEDAKKMLRRVRGTDDIEEEYSDLVAASEESKLVSHPWRNILQRRYRPQLTFAIAIP FFQQLTGINVIMFYAPVLFKTLGFADDASLMSAVITGLVNVFATFVSIVTVDRLGRRKLFLQG GTQMLACQIVVGSLIGAKFGFTGVADIPKGYAAFVVLFICAYVAGFAWSWGPLGWLVPSEIF PLEIRSAGQSITVSMNMLCTFIIAQAFLPMLCRFKFMLFFFFGAWVVVMTLFVAFFLPETKN VPIEEMVLVWKAHWYWGRFIRDEDVHVGGADVEMRSNGKVQAAKLP
