# Supplementary material for: Genome-Wide Identification and Expression Profiling of Sugar Transport Protein Response to Fusarium Head Blight in Wheat (Triticum aestivum L.)
Source: Plants (Basel). 2025 Sep 25;14(19):2976. doi: 10.3390/plants14192976 (PMC12526077; doi:10.3390/plants14192976)
Supplement: Supplementary file 1 [file plants-14-02976-s001.zip › Table S2.pdf]

Table S2. Information about the TaSTP genes in wheat.

Gene Name	Length of the coding sequence(bp)	Length of the amino acid sequence(aa)	Molecular weight of the amino acid sequence(kDa)	Isoelectric point
TaSTP1-2A	1533	510	54.6	9.53
TaSTP1-2B	1533	510	54.6	9.51
TaSTP1-2D	1533	510	54.48	9.61
TaSTP3-2A	1554	517	56.29	9.57
TaSTP3-2B	1554	517	56.3	9.51
TaSTP3-2D	1554	517	56.29	9.57
TaSTP3-3A.1	1527	508	55.67	9.59
TaSTP3-3B.1	1527	508	55.63	9.54
TaSTP3-3B.2	1545	514	55.83	9.41
TaSTP3-3D.1	1545	514	55.83	9.41
TaSTP3-3D.2	1527	508	55.36	9.39
TaSTP5-7A	1536	511	55.38	8.44
TaSTP5-7B	1536	511	55.33	8.59
TaSTP5-7D	1536	511	55.37	8.64
TaSTP6-2A	1581	526	57.45	9.2
TaSTP6-2B	1581	526	57.52	9.18
TaSTP6-2D	1812	603	65.98	9.29
TaSTP7-4A	1545	514	55.93	9.28
TaSTP7-5A.1	1563	520	56.3	9.06
TaSTP7-5A.2	1587	528	57.29	8.79
TaSTP7-5B.1	1542	513	55.76	9.05
TaSTP7-5B.2	1587	528	57.27	8.78
TaSTP7-5D.1	1563	520	56.44	9.23
TaSTP7-5D.2	1587	528	57.31	8.9
TaSTP7-5D.3	1545	514	55.95	9.2
TaSTP7-5D.4	1515	504	55.16	9.62
TaSTP8-5A	1551	516	56.28	9.33
TaSTP8-5B	1554	517	56.05	9.16
TaSTP8-5D	1554	517	56.21	9.27
TaSTP9-6A	1572	523	57.27	8.57
TaSTP9-6B	1572	523	57.41	8.43
TaSTP9-6D	1572	523	57.27	8.42
TaSTP10-1A.1	1578	525	56.57	9.36
TaSTP10-1A.2	1428	475	51.95	9.6
TaSTP10-1D	1575	524	56.64	9.01
TaSTP11-6A	1530	509	55.08	8.9
TaSTP13-4A	1548	515	56.9	8.96
TaSTP13-4B	1545	514	56.69	8.95
TaSTP13-4D	1545	514	56.7	8.95
TaSTP13-4B.1	1575	524	57	8.99
TaSTP13-4B.2	1848	615	67.04	8.59
TaSTP13-4D.1	1581	526	57.24	9.06

Table S2. (*continued*)

Gene Name	Length of the coding sequence(bp)	Length of the amino acid sequence(aa)	Molecular weight of the amino acid sequence(kDa)	Isoelectric point
TaSTP13-6A	1080	359	39.55	10.5
TaSTP13-6B	1578	525	58.34	8.81
TaSTP14-4B	1554	517	55.01	9.92
TaSTP14-4D	1548	515	54.87	10.5
TaSTP14-5A	1548	515	54.99	9.98
TaSTP15-2A	1545	514	55.48	9.61
TaSTP15-2B	1545	514	55.52	9.58
TaSTP15-2D	1545	514	55.61	9.58
TaSTP16-2A	1548	515	56.15	9.74
TaSTP16-2B	1374	457	50.18	10
TaSTP16-2D	1548	515	56.33	9.68
TaSTP17-2A	1524	507	54.26	9.51
TaSTP17-2B.1	1626	541	58.11	9.6
TaSTP17-2B.2	1524	507	54.26	9.62
TaSTP17-2D	1524	507	54.31	9.67
TaSTP18-2A	1542	513	54.32	9.7
TaSTP18-2B	1542	513	54.22	9.83
TaSTP18-2D	1542	513	54.23	9.75
TaSTP19-4A	1542	513	54.28	9.84
TaSTP19-7A	1545	514	54.45	9.89
TaSTP19-7D	1539	512	54.12	9.92
TaSTP22-1A	1515	504	56.26	9.18
TaSTP22-1B	1536	511	56.25	9.17
TaSTP22-1D	1536	511	56.24	9.17
TaSTP25-5A	1590	529	57.47	9.07
TaSTP25-5B	1587	528	57.43	8.86
TaSTP25-5D	1590	529	57.45	8.97
TaSTP26-5A	1566	521	56.55	9.26
TaSTP26-5B	1563	520	56.39	9.34
TaSTP26-5D	1563	520	56.47	9.26
TaSTP27-1A	1533	510	55.98	9.09
TaSTP27-1B	1566	521	57.41	9.11
TaSTP27-1D	1566	521	57.26	9.11
TaSTP27-5A.1	1158	385	41.27	9.79
TaSTP27-5A.2	1581	526	57.26	8.98
TaSTP28-1B.1	1515	504	54.49	9.16
TaSTP28-1B.2	1515	504	54.59	9.16
TaSTP28-3A	1533	510	54.62	9.37
TaSTP28-3B	1386	461	49.82	9.36
TaSTP28-3D	1539	512	54.79	9.18
