# Supplementary material for: Genome-Wide Identification and Expression Profiling of Sugar Transport Protein Response to Fusarium Head Blight in Wheat (Triticum aestivum L.)
Source: Plants (Basel). 2025 Sep 25;14(19):2976. doi: 10.3390/plants14192976 (PMC12526077; doi:10.3390/plants14192976)
Supplement: Supplementary file 1 [file plants-14-02976-s001.zip › Table S3.pdf]

Table S3. Named of TaSTP genes in wheat.

Chinese Spring database (IWGSCv2.1) gene-ID	Chinese Spring database (IWGSCv1.1) gene-ID	Gene Name	Rice/Arabidopsis thaliana orthologs	Named by
TraesCS2A03G0795100.1	TraesCS2A02G340600.1	TaSTP1-2A	OsSTP1	This study
TraesCS2B03G0880300.1	TraesCS2B02G338400.1	TaSTP1-2B	OsSTP1	This study
TraesCS2D03G0731000.1	TraesCS2D02G318200.1	TaSTP1-2D	OsSTP1	This study
TraesCS2A03G0638900.1	TraesCS2A02G259500.1	TaSTP3-2A	OsSTP3	Huai et al.,2022
TraesCS2B03G0731200.1	TraesCS2B02G282400.1	TaSTP3-2B	OsSTP3	Huai et al.,2022
TraesCS2D03G0609300.1	TraesCS2D02G264200.1	TaSTP3-2D	OsSTP3	Huai et al.,2022
TraesCS3A03G1110100.1	TraesCS3A02G475200.1	TaSTP3-3A.1	OsSTP3	This study
TraesCS3B03G1287600.1	TraesCS3B02G518800.1	TaSTP3-3B.1	OsSTP3	This study
TraesCS3B03G1287800.1	TraesCS3B02G518900.1	TaSTP3-3B.2	OsSTP3	This study
TraesCS3D03G1037600.1	TraesCS3D02G470200.1	TaSTP3-3D.1	OsSTP3	This study
TraesCS3D03G1037500.1	TraesCS3D02G470300.1	TaSTP3-3D.2	OsSTP3	This study
TraesCS7A03G0691000.1	TraesCS7A02G288800.1	TaSTP5-7A	OsSTP5	This study
TraesCS7B03G0523600.1	TraesCS7B02G185800.1	TaSTP5-7B	OsSTP5	This study
TraesCS7D03G0664000.1	TraesCS7D02G286600.1	TaSTP5-7D	OsSTP5	This study
TraesCS2A03G0431900.1	TraesCS2A02G205500.1	TaSTP6-2A	OsSTP6	Huai et al.,2019
TraesCS2B03G0559500.1	TraesCS2B02G232900.1	TaSTP6-2B	OsSTP6	Huai et al.,2019
TraesCS2D03G0440600LC.1	TraesCS2D02G230100LC.1	TaSTP6-2D	OsSTP6	Huai et al.,2019
TraesCS4A03G0786500.1	TraesCS4A02G314900.1	TaSTP7-4A	AtSTP7	This study
TraesCS5A03G0579700.1	TraesCS5A02G226700.1	TaSTP7-5A.1	AtSTP7	This study
TraesCS5A03G0579800.1	TraesCS5A02G226800.1	TaSTP7-5A.2	AtSTP7	This study
TraesCS5B03G0593400.1	TraesCS5B02G225300.1	TaSTP7-5B.1	AtSTP7	This study
TraesCS5B03G0594400.1	TraesCS5B02G225400.1	TaSTP7-5B.2	AtSTP7	This study
TraesCS5D03G0543700.1	TraesCS5D02G234100.1	TaSTP7-5D.1	AtSTP7	This study
TraesCS5D03G0544100.1	TraesCS5D02G234200.1	TaSTP7-5D.2	AtSTP7	This study
TraesCS5D03G1199400.1	TraesCS5D02G549900.1	TaSTP7-5D.3	AtSTP7	This study
TraesCS5D03G1227500.1	TraesCS5D02G558100.1	TaSTP7-5D.4	AtSTP7	This study

Table S3. (Continued)

Chinese Spring database (IWGSCv2.1) gene-ID	Chinese Spring database (IWGSCv1.1) gene-ID	Gene Name	Rice/Arabidopsis thaliana orthologs	Named by
TraesCS5A03G0001700.1	TraesCS5A02G001100.1	TaSTP8-5A	OsSTP8	This study
TraesCS5B03G0001700.1	TraesCS5B02G001000.1	TaSTP8-5B	OsSTP8	This study
TraesCS5D03G0002900.1	TraesCS5D02G001600.1	TaSTP8-5D	OsSTP8	This study
TraesCS6A03G0318600.1	TraesCS6A02G13400.10	TaSTP9-6A	OsSTP9	This study
TraesCS6B03G0411200.1	TraesCS6B02G162200.1	TaSTP9-6B	OsSTP9	This study
TraesCS6D03G0274100.1	TraesCS6D02G123300.1	TaSTP9-6D	OsSTP9	This study
TraesCS1A03G0158800.1	TraesCS1A02G064900.1	TaSTP10-1A.1	OsSTP10	This study
TraesCS1A03G0158800.2	TraesCS1A02G064900.2	TaSTP10-1A.2	OsSTP10	This study
TraesCS1D03G0147200.1	TraesCS1D02G065700.1	TaSTP10-1D	OsSTP10	This study
TraesCS6A03G0638400.1	TraesCS6A02G231700.1	TaSTP11-6A	OsSTP11/OsSTP11	This study
TraesCS4A03G0132000.1	TraesCS4A02G066200.1	TaSTP13-4A	AtSTP13	Huai et al.,2020
TraesCS4B03G0663100.1	TraesCS4B02G243500.1	TaSTP13-4B	AtSTP13	Huai et al.,2020
TraesCS4B03G0955900.1	TraesCS4B02G371700.1	TaSTP13-4B.1	OsSTP13	This study
TraesCS4B03G0955900.2	TraesCS4B02G371700.2	TaSTP13-4B.2	OsSTP13	This study
TraesCS4D03G0585200.1	TraesCS4D02G243100.1	TaSTP13-4D	AtSTP13	Huai et al.,2020
TraesCS4D03G0837600.1	TraesCS4D02G365800.1	TaSTP13-4D.1	OsSTP13	This study
TraesCS6A03G0905100.1	TraesCS5A02G542600.1	TaSTP13-5A.1	OsSTP13	This study
TraesCS6B03G1088100.1	TraesCS5A02G542600.2	TaSTP13-5A.2	OsSTP13	This study
TraesCS4B03G0901000.1	TraesCS6A02G351600.1	TaSTP13-6A	AtSTP13	This study
TraesCS4D03G0793400.1	TraesCS6B02G384700.1	TaSTP13-6B	AtSTP13	This study
TraesCS5A03G1206800.1	TraesCS4B02G346400.1	TaSTP14-4B	OsSTP14	This study
TraesCS2A03G0795600.1	TraesCS4D02G341400.1	TaSTP14-4D	OsSTP14	This study
TraesCS2B03G0880400.1	TraesCS5A02G515200.1	TaSTP14-5A	OsSTP14	This study
TraesCS2D03G0731300.1	TraesCS2A02G340700.1	TaSTP15-2A	OsSTP15	This study
TraesCS2A03G0795900.1	TraesCS2B02G338500.1	TaSTP15-2B	OsSTP15	This study
TraesCS2B03G0880800.1	TraesCS2D02G318300.1	TaSTP15-2D	OsSTP15	This study

Table S3. (Continued)

Chinese Spring database (IWGSCv2.1) gene-ID	Chinese Spring database (IWGSCv1.1) gene-ID	Gene Name	Rice/Arabidopsis thaliana orthologs	Named by
TraesCS2D03G0731500.1	TraesCS2A02G340800.1	TaSTP16-2A	OsSTP16	This study
TraesCS2A03G0796100.1	TraesCS2B02G338600.1	TaSTP16-2B	OsSTP16	This study
TraesCS2B03G0880900.1	TraesCS2D02G318400.1	TaSTP16-2D	OsSTP16	This study
TraesCS2B03G0880900.2	TraesCS2A02G340900.1	TaSTP17-2A	OsSTP17	This study
TraesCS2D03G0731600.1	TraesCS2B02G338700.1	TaSTP17-2B.1	OsSTP17	This study
TraesCS2A03G0797900.1	TraesCS2B02G338700.2	TaSTP17-2B.2	OsSTP17	This study
TraesCS2B03G0882300.1	TraesCS2D02G318500.1	TaSTP17-2D	OsSTP17	This study
TraesCS2D03G0734500.1	TraesCS2A02G341400.1	TaSTP18-2A	OsSTP18	This study
TraesCS4A03G0958600.1	TraesCS2B02G339100.1	TaSTP18-2B	OsSTP18	This study
TraesCS7A03G0204800.1	TraesCS2D02G319100.1	TaSTP18-2D	OsSTP18	This study
TraesCS7D03G0183500.1	TraesCS4A02G384900.1	TaSTP19-4A	OsSTP19	This study
TraesCS1A03G0842000.1	TraesCS7A02G085900.1	TaSTP19-7A	OsSTP19	This study
TraesCS1B03G0967600.1	TraesCS7D02G080200.1	TaSTP19-7D	OsSTP19	This study
TraesCS1D03G0810500.1	TraesCS1A02G341100.1	TaSTP22-1A	OsSTP22	This study
TraesCS5A03G0260200.1	TraesCS1B02G353800.1	TaSTP22-1B	OsSTP22	This study
TraesCS5B03G0481800.1	TraesCS1D02G343200.1	TaSTP22-1D	OsSTP22	This study
TraesCS5D03G0446300.1	TraesCS5A02G182600.1	TaSTP25-5A	OsSTP25	Huai et al.,2022
TraesCS5A03G0579300.1	TraesCS5B02G180800.1	TaSTP25-5B	OsSTP25	Huai et al.,2022
TraesCS5B03G0592600.1	TraesCS5D02G187100.1	TaSTP25-5D	OsSTP25	Huai et al.,2022
TraesCS5D03G0543500.1	TraesCS5A02G226500.1	TaSTP26-5A	OsSTP26	Huai et al.,2022
TraesCS1A03G0576300.1	TraesCS5B02G225100.1	TaSTP26-5B	OsSTP26	Huai et al.,2022
TraesCS1B03G0668700.1	TraesCS5D02G233900.1	TaSTP26-5D	OsSTP26	Huai et al.,2022
TraesCS1D03G0549500.1	TraesCS1A02G219500.1	TaSTP27-1A	OsSTP27	This study
TraesCS5A03G1267900.1	TraesCS1B02G232900.1	TaSTP27-1B	OsSTP27	This study
TraesCS5A03G1267900.2	TraesCS1D02G221100.1	TaSTP27-1D	OsSTP27	This study
TraesCS1B03G1217700.1	TraesCS1B02G454800.1	TaSTP28-1B.1	OsSTP28	This study

Table S3. (Continued)

Chinese Spring database (IWGSCv2.1) gene-ID	Chinese Spring database (IWGSCv1.1) gene-ID	Gene Name	Rice/Arabidopsis thaliana orthologs	Named by
TraesCS1B03G1217700.2	TraesCS1B02G454800.2	TaSTP28-1B.2	OsSTP28	This study
TraesCS3A03G0225200.1	TraesCS3A02G102800.1	TaSTP28-3A	OsSTP28	This study
TraesCS3B03G0277400.1	TraesCS3B02G120300.1	TaSTP28-3B	OsSTP28	This study
TraesCS3D03G0216400.1	TraesCS3D02G105000.1	TaSTP28-3D	OsSTP28	This study
